# Coralsnake Venomics: Analyses of Venom Gland Transcriptomes and Proteomes of Six Brazilian Taxa

**DOI:** 10.3390/toxins9060187

**Published:** 2017-06-08

**Authors:** Steven D. Aird, Nelson Jorge da Silva, Lijun Qiu, Alejandro Villar-Briones, Vera Aparecida Saddi, Mariana Pires de Campos Telles, Miguel L. Grau, Alexander S. Mikheyev

**Affiliations:** 1 Division of Faculty Affairs, Okinawa Institute of Science and Technology Graduate University, 1919-1 Tancha, Onna-son, Kunigami-gun, Okinawa-ken 904-0495, Japan; 2 Ecology and Evolution Unit, Okinawa Institute of Science and Technology Graduate University, 1919-1 Tancha, Onna-son, Kunigami-gun, Okinawa-ken 904-0495, Japan; lijun.qiu@oist.jp (L.Q.); miguel.graulopez@oist.jp (M.L.G.); alexander.mikheyev@oist.jp (A.S.M.); 3 Programa de Pós-Graduação em Ciências Ambientais e Saúde, Pontifícia Universidade Católica de Goiás, Goiânia, Goiás 74605-140, Brazil; nelson.jorge.silvajr@gmail.com (N.J.d.S.J.); verasaddi@gmail.com (V.A.S.); tellesmpc@gmail.com (M.P.d.C.T.); 4 Research Support Division, Okinawa Institute of Science and Technology Graduate University, 1919-1 Tancha, Onna-son, Kunigami-gun, Okinawa-ken 904-0495, Japan; avillar@oist.jp; 5 Laboratório de Oncogenética e Radiobiologia da Associação de Combate ao Câncer em Goiás, Universidade Federal de Goiás, Rua 239 no. 52—Setor Universitário, Goiânia, Goiás 74065-070, Brazil; 6 Laboratório de Genética & Biodiversidade, Universidade Federal de Goiás, Goiânia, Goiás 74690-900, Brazil

**Keywords:** coralsnakes, *Micrurus*, venom gland transcriptomes, proteomes, 3FTx, phospholipase A_2_, molecular models, novel toxins

## Abstract

Venom gland transcriptomes and proteomes of six *Micrurus* taxa (*M. corallinus*, *M. lemniscatus carvalhoi*, *M. lemniscatus lemniscatus*, *M. paraensis*, *M. spixii spixii*, and *M. surinamensis*) were investigated, providing the most comprehensive, quantitative data on *Micrurus* venom composition to date, and more than tripling the number of *Micrurus* venom protein sequences previously available. The six venomes differ dramatically. All are dominated by 2–6 toxin classes that account for 91–99% of the toxin transcripts. The *M. s. spixii* venome is compositionally the simplest. In it, three-finger toxins (3FTxs) and phospholipases A_2_ (PLA_2_s) comprise >99% of the toxin transcripts, which include only four additional toxin families at levels ≥0.1%. *Micrurus l. lemniscatus* venom is the most complex, with at least 17 toxin families. However, in each venome, multiple structural subclasses of 3FTXs and PLA_2_s are present. These almost certainly differ in pharmacology as well. All venoms also contain phospholipase B and vascular endothelial growth factors. Minor components (0.1–2.0%) are found in all venoms except that of *M. s. spixii*. Other toxin families are present in all six venoms at trace levels (<0.005%). Minor and trace venom components differ in each venom. Numerous novel toxin chemistries include 3FTxs with previously unknown 8- and 10-cysteine arrangements, resulting in new 3D structures and target specificities. 9-cysteine toxins raise the possibility of covalent, homodimeric 3FTxs or heterodimeric toxins with unknown pharmacologies. Probable muscarinic sequences may be reptile-specific homologs that promote hypotension via vascular mAChRs. The first complete sequences are presented for 3FTxs putatively responsible for liberating glutamate from rat brain synaptosomes. *Micrurus* C-type lectin-like proteins may have 6–9 cysteine residues and may be monomers, or homo- or heterodimers of unknown pharmacology. Novel KSPIs, 3× longer than any seen previously, appear to have arisen in three species by gene duplication and fusion. Four species have transcripts homologous to the nociceptive toxin, (MitTx) α-subunit, but all six species had homologs to the β-subunit. The first non-neurotoxic, non-catalytic elapid phospholipase A_2_s are reported. All are probably myonecrotic. Phylogenetic analysis indicates that the six taxa diverged 15–35 million years ago and that they split from their last common ancestor with Old World elapines nearly 55 million years ago. Given their early diversification, many cryptic micrurine taxa are anticipated.

## 1. Introduction

The New World coralsnakes constitute a taxonomic complex of more than 70 species, traditionally divided into three genera (*Micruroides*, *Leptomicrurus*, and *Micrurus*) pertaining to the Family Elapidae. Because of their fossorial, semi-fossorial, or in a few cases, aquatic habits, coralsnakes are less often encountered by humans than are pit vipers occupying the same habitats. Coralsnakes produce smaller quantities of venom than pit vipers and other elapids of comparable length and they are more difficult to handle and to extract. Add to that the extreme difficulty of maintaining them in captivity for venom production, and it is not difficult to understand why venom chemistry of coralsnakes has lagged well behind that of viperids and larger elapids. Despite nearly 80 years of *Micrurus* venom research, fewer than 150 papers characterize *Micrurus* venoms or specific toxin constituents. Venoms of less than one-fourth of the recognized taxa have ever been examined in even the most superficial way. We have a modest understanding of the biochemical composition of only about five species, and pharmacologically, we know even less. To date, the coralsnake venom literature contains only two transcriptomic studies (*Micrurus fulvius* and *M. tener*) [1,2].

Accordingly, we characterized the venoms of six Brazilian *Micrurus* species (*M. corallinus*, *M. l. carvalhoi*, *M. l. lemniscatus*, *M. paraensis*, *M. s. spixii*, and *M. surinamensis*) that display great morphological and ecological diversity. Partial characterizations exist for *M. corallinus*, *M. surinamensis*, and *M. l. lemniscatus* venoms, but little has been reported for *M. s. spixii* and *M. l. carvalhoi*, and there have been no reports regarding *M. paraensis*. For each species, a venom gland transcriptome was sequenced using Illumina technology and venom peptides were identified with LC-MS.

## 2. Results and Discussion

### 2.1. Specimen Collection

Of the roughly 30 species of *Micrurus* that occur in the Amazon Basin, five are restricted to tropical Amazonian forests, while others, like *Micrurus paraensis* and *M. s. spixii*, can exist even in areas of contact with Amazonian cerrado (short-tree forest) [3,4,5,6]. The *M. lemniscatus* complex has two semi-aquatic taxa that are found in the Amazon Basin (*M. l. lemniscatus* and *M. l. helleri*) and a third (*M. l. carvalhoi*) that has the largest distribution of any coralsnake, from Rio Grande do Sul to the Northeast and the eastern Amazon, where it occupies primary and secondary cerrado, parts of the caatinga (a shrubby desert region in northeastern Brazil) and Atlantic forest [5]. *Micrurus surinamensis* exhibits similar adaptability, occurring not only in Amazonian rivers and their tributaries, but also at more northern latitudes, in the cerrado, and in areas of gallery forest along the Rio Araguaia [7]. *Micrurus corallinus* is associated with Atlantic forest, including zones of contact with cerrado [5].

Habitat utilization is very poorly known for most coralsnake species, and even in regions that appear more or less uniform (Amazonian forest or cerrado), coralsnakes may be present or absent, apparently depending upon less obvious habitat characteristics such as leaf litter. Ultimately, mineral content or soil pH, which govern plant species composition, may cause subtle differences in coralsnake habitats, thereby influencing distributions. Even semi-aquatic and aquatic species may be affected by such factors [5].

### 2.2. Transcriptomics and Proteomics

#### 2.2.1. Characterization of Transcriptomes

The six transcriptomes yielded 1,051,787 contigs and the percentages of all reads assembled varied from 87.8 to 96.1%. Mean contig length ranged from 494 bp to 569 bp. Various other statistics are also available in Table S1.

**Figure 1:**
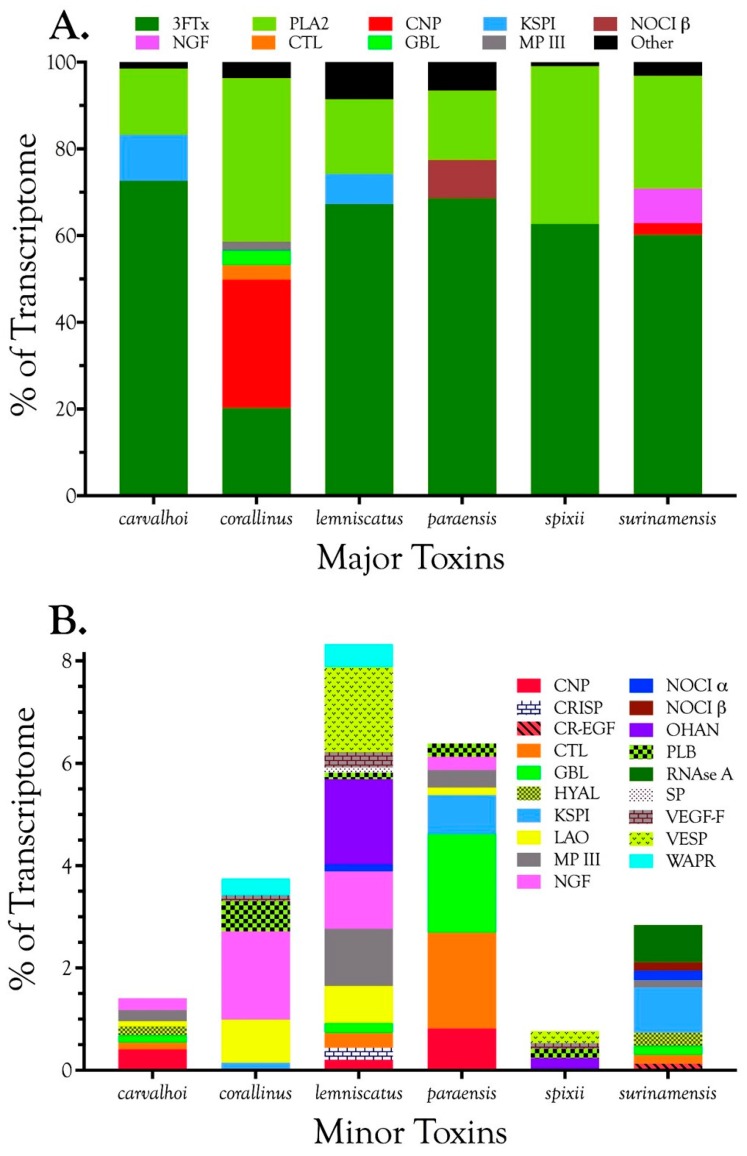
These Brazilian *Micrurus* venoms all contain three-finger toxins (3FTxs) and phospholipases A_2_ (PLA_2_s), but but they vary greatly in the relative proportions and subclasses thereof, and in the types and amounts of minor toxins as well. (**A**) Major toxins comprising ≥2% of the toxin portion a given transcriptome. The “other” portion of each venom (black) was comprised of minor components; (**B**) Minor toxins representing between 0.1% and 2.0% of the toxin transcriptome. Each venom contained still other toxins at trace levels, each amounting to less than 0.1% of the transcriptome. *Micrurus s. spixii* possesses the simplest venom, with 3FTxs and PLA_2_s accounting for just over 99% of the transcriptome, and comprising only six major and minor toxin classes.

Venom gland transcriptomes of the six *Micrurus* species varied dramatically in composition. All transcriptomes were dominated by 2–6 toxin classes that accounted for 91.4–99.0% of the transcripts (Figure 1A; Table S2). The *M. s. spixii* transcriptome was compositionally the simplest. Three-finger toxins (3FTx) and phospholipases A_2_ (PLA_2_) amounted to just over 99.0% of the transcriptome, which comprised only four additional toxin families at levels of ≥0.1% (Figure 1A; Table S2). Other toxin families are present in all six venoms at trace levels (<0.005%). Venom of *M. l. lemniscatus* was the most complex, with at least 17 toxin families (Figure 1; Table S2). However, in each venome, multiple structural subclasses of 3FTXs and PLA_2_s are present. These variants have very different 3D structures and almost certainly differ in pharmacology as well.

In addition to 3FTxs and PLA_2_s, all venoms also contained a putative nociceptive toxin (NOCI) subunit β (but not necessarily α), phospholipase B (PLB), and short vascular endothelial growth factors (VEGF-Fs) [8]. Minor components (0.1–2.0%) were found in venoms of all species except that of *M. s. spixii* (Table S2). The most abundant venom components after 3FTxs and PLA_2_s differed from venom to venom. In both *M. l. carvalhoi* and *M. l. lemniscatus* venoms, the next most abundant components were Kunitz serine protease inhibitors (KSPIs), a highly diversified venom protein family (Figure 1A; Table S2). In *M. corallinus* venom, C-type natriuretic peptide (CNP) was the next most abundant component, representing more than 29.5% of the transcriptome, followed by a C-type lectin-like protein (CTL) (3.4%), galactose-binding lectin (GBL) (3.4%), and a metalloprotease (MP) (2.1%). GBLs are normally extremely minor venom constituents (~0.1%) [9,10,11]. *Micrurus paraensis* venom had only one other major component beside 3FTxs and PLA_2_s. That was the nociceptive toxin β subunit (8.9%), homologous to MitTxβ from the venom of *M. tener*; however, interestingly, *M. paraensis* does not appear to produce the α subunit. At least from the western Amazon basin, *Micrurus surinamensis* venom is generally devoid of enzymatic components [12], but our transcriptome from this species contained 7.9% nerve growth factor (NGF), to which arginine esterase activity has been ascribed [13,14]. This is remarkable because this protease generally comprises no more than 0.2–0.7% of most venoms [10,11,15]. However, in the venom sample used for proteomics, this transcript was not even identified. Different specimens were used for the transcriptome and proteome, but both were collected in the vicinity of Altamira, Pará at localities separated by only 50 km. In a specimen from Estreito, Maranhão, about 800 km distant, NGF was present at a level of 0.4%. This is but one example of geographic and individual variation in this species. CNP was the remaining major component of the *M. surinamensis* transcriptome, at 2.7% (Figure 1; Table S2).

#### 2.2.2. Characterization of Proteomes

The proteomes showed reasonable qualitative agreement with the transcriptomes, in that all but the most minor components of the transcriptomes were generally represented in the proteomes (Table S2). However, in contrast to our recent studies of *Protobothrops* venoms [10,11], the quantitative correspondence between transcriptomes and proteomes was extremely poor (Table S2). In large part, this was because the transcriptomes and venom samples came from different snakes, but this does not provide an entirely satisfactory justification.

3FTxs proved especially problematic in that their estimated abundances tended to be drastically lower in the proteomes than the transcriptomes (Table S2). We can offer no adequate explanation for this discrepancy. The lower representation of 3FTxs inflated values for all other venom proteins. Given the disparities between transcriptome and proteome, we ran a 4–12% SDS-DTT Bis-Tris polyacrylamide gel in MES buffer (pH 8.3), using *M. l. carvalhoi* venom (62.5, 125, 187.5 µg/well) to determine whether the transcriptomic or the proteomic result was accurate (Figure S1). Mass spectrometry was used to confirm what protein families were present in each band on the gel. 3FTxs were present in all of the lower bands on the gel (Fractions 10–16) and in some of the upper bands as well (3–6). Clearly, the venom is amply supplied with 3FTxs, as the transcriptome implies. While two of the digestions were enzymatic, the formic acid cleavage essentially cannot fail, so it seems unlikely that generation of peptides suitable for mass spectrometry was the source of the problem. A more likely explanation has to do with identification of the peptides sequenced by mass spectrometry. Since protein identification relies on interpretation of the spectra, the tremendous diversity of micrurine 3FTxs and the use of different specimens for transcriptomes and proteomes may have partially confounded peptide identification.

In 1995, using traditional Edman degradation, Aird and Silva, Jr. began sequencing toxins from *M. surinamensis* collected in the vicinity of Letícia, Colombia. Funding ran out before the sequences did and the partial sequences were never published, except for the first 37 residues of a short neurotoxin [16]. In 2008, Olamendi-Portugal et al. [17], using a combination of Edman degradation and mass spectrometry, completed the sequences of six toxins, from venom of specimens captured at Iquitos, Loreto, Peru. Both sets of sequences were identical, to the extent of our sequences. However, in this study, our two venom samples came from Pará and Maranhão, Brazil, at the eastern end of the *M. surinamensis* range. We found good matches for three of the sequences (MS5, MS4, and MS2) of Olamendi-Portugal et al. [17], with percent identities ranging from 89.1–97.7%. However, the best matches to their sequences MS1, MS3, and MS11 were very poor, displaying percent identities of only 44.9–50.1%. No BLAST hit for our Letícia, Colombia short neurotoxin sequence was found in the *M. surinamensis* transcriptome from Pará, Brazil.

#### 2.2.3. 3-Finger Toxins (3FTx)

**Figure 2:**
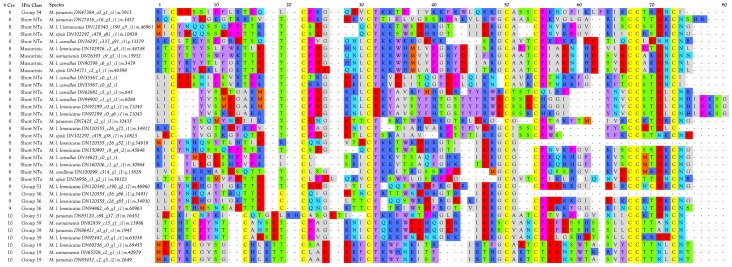
*Micrurus* venoms are rich in 3FTxs displaying an astonishing variety of primary structures. Micrurine 3FTxs may possess 8, 9, or 10 Cys residues with different disulfide patterns in each group. Pharmacologies are almost entirely unknown. Some probably target nicotinic acetylcholine receptors of reptilian neuromuscular junctions, but their potential targets in mammals are unknown. Most 3FTxs have 21-residue signal peptides. In the interest of creating a figure of manageable size for the journal format, all signal peptides have been deleted here, and only sequences originating in this study have been included. Full sequences of all 184 micrurine 3FTxs can be found in Figure S2.

**Figure 3:**
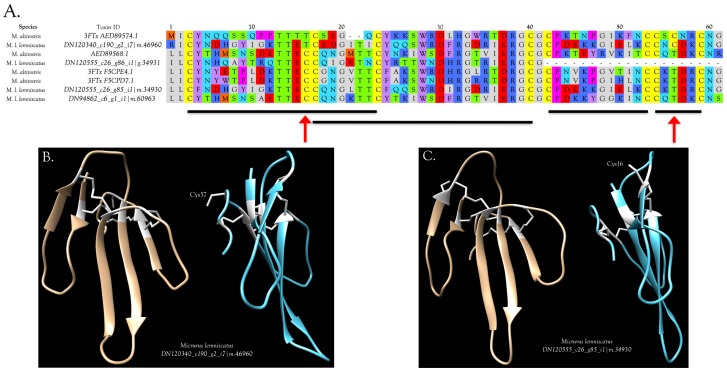
Primary and 3D structures of 9-Cys 3FTxs from *Micrurus* venoms include two structural subclasses. (**A**) To date, micrurine 3FTxs with 9 cysteines have been found only in venoms of *Micrurus l. lemniscatus* and *M. altirostris*; however, among these, the extra cysteine can appear in either of two positions, indicated by red arrows. Putative conserved disulfide bonds are indicated with black bars. Signal peptides, 21 residues in length, were almost invariant (MKTLL LTLVV VTIVC LDFGH T). *M. l. lemniscatus* toxin DN120340 had an L/Q substitution in position 4 and a V/L substitution in position 14; (**B**) Front and side views of the ribbon model of *M. l. lemniscatus* DN120340; (**C**) Front and side views of the ribbon model of *M. l. lemniscatus* DN120555. SWISS-MODEL was used to select the best templates for the *Micrurus* toxins (*Laticauda semifasciata*, erabutoxin, 2era.1.A for *M. l. lemnsicatus* DN120340 and *Naja atra* cobrotoxin (1coe.1.A) for *M. l. lemniscatus* DN120555) and to construct a preliminary model. Then models were refined and energy minimizations were performed with UCSF Chimera [21]. Disulfide bonds are shown in white.

Three-finger toxins are significant components of all six venoms examined in this study, comprising from 20.3 to 72.6% of the transcriptomes (Figure 1, Figure 2 and Figure S2; Table S2). 3FTxs having 8, 9, and 10 cysteines are present (Figure 2 and Figure S2), although interestingly, among New World elapids, 9-Cys toxins have been found only in the venoms of *M. l. lemniscatus* and *M. altirostris* to date (Figure 3 and Figure S2). Two of the 9-Cys toxins, one from each species, have the extra Cys in position 56 (Figure 3A,B and Figure S2). A BLAST search failed to locate any Old World elapid 3FTx with this disulfide bond arrangement in the top 100 hits [18,19]. The remaining six toxins have the extra Cys residue in position 16 (Figure 3A,C), giving the two subclasses of 3FTx very different 3D profiles49. 

This arrangement apparently has arisen independently in only two Old World elapids, *Walterinnesia aegyptia* (C1IC48.1) [20] and *Micropechis ikaheka* (AHZ08817.1, Paiva, O.K. unpublished). The existence of 9-Cys toxins suggests the possibility of covalent homodimeric 3FTxs. Alternatively, they could form heterodimers with some other toxin class that also has a free Cys, or it may be that the Cys is actually free and is involved in target binding.

##### 3FTxs with 10 Cysteine Residues

**Figure 4:**
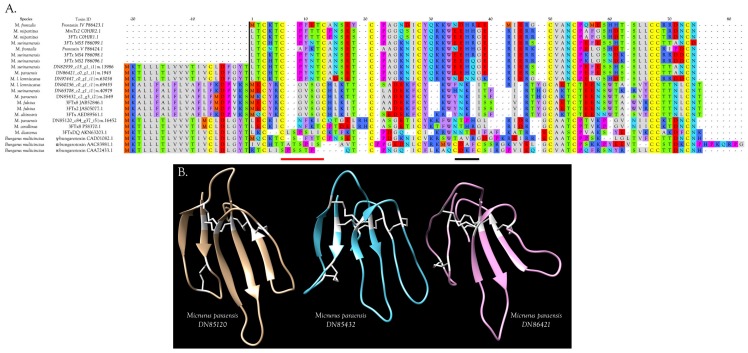
Micrurine 3FTxs with 10 cysteine residues include several subclasses. (**A**) 3FTxs with 10 cysteines show the disulfide bond pattern of γ-bungarotoxin (red bar), but not of α- and κ-bungarotoxins (black bar). Thus, homologs of the latter two 3FTx subclasses probably are not found in New World coralsnake venoms. Residues to the left of the black vertical line constitute signal peptides; (**B**) Ribbon models of three different subclasses of 10-Cys 3FTxs from the venom of *M. paraensis*: DN85120 (tan), DN85432 (blue), and DN86421 (violet), from (**A**). 3D representations were made with UCSF Chimera [21] based on models generated with SWISS-MODEL, using the structure of candoxin (1jgk.1.A) as a template for DN85120, bucandin (1ijc.1.A) as a template for DN85432, both from *Bungarus candidus* venom, and *Bungarus multicinctus* γ-bungarotoxin (1mr6.1.A) for DN86421. Disulfide bonds are shown in white.

Micrurine 3FTx toxins with 10 Cys residues (19 sequences) have the disulfide bond arrangement of γ-bungarotoxin, rather than that of α- or κ-bungarotoxin (Figure 4A and Figure S2); thus, close homologs of these two better-known 3FTx subclasses do not appear to be present in *Micrurus* venoms. γ-bungarotoxin is a neuronal nAChR antagonist with unique specificity [22,23]. 10-Cys 3FTxs fall into a subclass of elapid 3FTxs originally known as “weak neurotoxins”, under the assumption that they target nAChRs ineffectively. Later they were dubbed “non-conventional” toxins [24]. While some of the Old World elapid 3FTxs eventually proved to be neuronal toxins, rather than antagonists of neuromuscular junction nAChRs, it finally became clear that many “non-conventional” toxins do not target nAChRs at all [25,26,27].

Micrurine 10-Cys 3FTxs fall into at least three sub-subclasses, represented by *M. paraensis*: toxins DN85432, DN86421, and DN85120 (Figure 4A,B and Figure S2). Homologs of DN85432 have radically different signal peptides rich in phenylalanine and commence with the N-terminal sequence MECYR, suggesting unusual pharmacology (Figure 4A). Homologs of DN86421 and DN 85120 have similar signal peptides. The former have N-terminal sequences commencing with LTCK/HT, while the latter have LECKI, with many other internal sequence differences also (Figure 4A).

##### 3FTxs and GABA Receptors

Rosso et al. [28] reported that micrurotoxins 1 and 2, from the venom of Costa Rican *Micrurus mipartitus*, bind to GABAA receptors at subnanomolar concentrations. Nicotinic acetylcholine receptors (nAChR) were not affected by these toxins. The two micrurotoxins, which differ by a single Arg/His substitution at position 33, bind to a benzodiazepine-like binding site at the α+/β− subunit interface, allosterically enhancing receptor susceptibility to agonist, which potentiates receptor opening and desensitization [28]. Unfortunately, because no structures were available, it is not possible to know whether any homologs of the micrurotoxins are found in our transcriptomes.

##### 3FTxs with 8 Cysteine Residues

The overwhelming majority of micrurine 3FTxs with eight cysteines follow the classic α-neurotoxin/cardiotoxin disulfide bond pattern (3–24, 17–41, 43–54, 55–60) [29,30,31]. Numbering varies slightly with the toxin or alignment in question. While most micrurine 8-Cys 3FTxs terminate one or two residues beyond the most C-terminal Cys, some of the toxins that we sequenced have C-terminal extensions of 2–14 amino acids (Figure 2 and Figure S2). Many of these probably target nicotinic acetylcholine receptors of reptilian or fish neuromuscular junctions, but in the absence of pharmacological studies, little more can be said.

**Figure 5:**
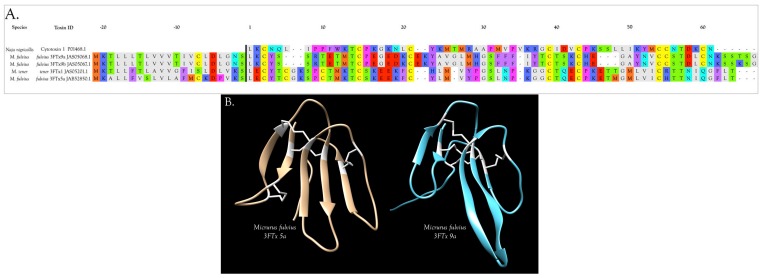
3FTxs bearing 8 cysteine residues show two very different disulfide bond patterns. (**A**) The classic short α-neurotoxin/cardiotoxin pattern, represented above by *Naja nigricollis* Cardiotoxin 1 and by *Micrurus fulvius* 3FTxs 9a & b, is employed by all but two micrurine 3FTxs (*M. tener* 3FTx1 and *M. fulvius* 3FTx5a). The novel disulfide in the latter two toxins is indicated with a red bar. Conserved disulfides are denoted with black bars. Signal peptides are shown to the left of the black vertical line; (**B**) Ribbon models of the two subclasses of 8-Cys 3FTxs from the venom of *M. fulvius*: 3FTx 5a (JAB52850.1) (tan) and 3FTx 9a (JAS05068.1) (blue) from (**A**). Disulfide bonds are shown in white. SWISS-MODEL selected bucandin (1f94.1.A) from *Bungarus candidus* venom as the best template for *M. fulvius* 3FTx 5a, and 3FTx 3b (4rud.1.B) from *M. fulvius* venom as the best template for *M. fulvius* 3FTx 9a. Models were refined and energy minimizations were performed using UCSF Chimera [21].

Interestingly, two toxins from *Micrurus fulvius* and *Micrurus tener* introduce an entirely new structure in which the Cys54-Cys60 disulfide is absent and a new disulfide has apparently formed between Cys6 and Cys11 (Figure 5A). The magnitude of the resulting structural variation is readily apparent when comparing ribbon models of the two subclasses (Figure 5B). In addition, the signal peptides of these two classes show numerous differences (Figure 5A).

##### Muscarinic Toxins (MTx)

Toxins that agonize or antagonize muscarinic acetylcholine receptors with far greater specificity than small, organic ligands [32,33,34,35,36], and that bind with low nanomolar affinity, were first discovered in the venoms of mambas (*Dendroaspis* sp.) [37,38,39]. Muscarinic toxins, 3FTxs with eight cysteines, account for about 2% of crude *Dendroaspis angusticeps* venom, by mass [40]. Paradoxically, mamba venoms contain both muscarinic acetylcholine receptor (mAChR) agonists and antagonists, which, to the best of our knowledge, has never been explained.

mAChRs have been classified into five types (M1–M5) (Table 1), with the M3 type predominating in epithelial and smooth muscle cells [41]. M3-mAChRs mediate endothelium-dependent vasorelaxation in coronary arterial circulation [42], and activation of M3 and M5 mAChRs in the vasculature causes vasodilation [43] (Table 1). Neither m1- nor m4-toxin binds to M2, M3, or M5 receptors [33,35]. In contrast, MT2 acts as a partial agonist at M3-mAChRs [35]. Jolkkonen et al. [44] opined that MTα and MTβ, two muscarinic toxins from *Dendroaspis polylepis* venom, are probably mAChR agonists, based on their capacity to contract guinea pig ileum; thus, these toxins would be expected to induce vasodilation and hypotension. Ryberg et al. [45] have shown that through its actions on muscarinic receptors, acetylcholine causes vasodilation of rat carotid and submandibular arteries and vasoconstriction of jugular and submandibular veins. Acetylcholine, can cause vasodilation or vasoconstriction, depending upon the mAChR class to which it binds. Mamba muscarinic toxins may act as either agonists or antagonists, but it appears that in a well coordinated strategy, they simultaneously agonize vasodilatory muscarinic receptors and antagonize vasoconstrictive receptors, causing vasodilation of all vascular beds, profound hypotension, and circulatory shock [46] (Table 1).

In overall structure, MTs resemble traditional short α-neurotoxins that antagonize nAChRs [63]. Servent et al. [64] outlined structural attributes that characterize *D. angusticeps* muscarinic toxins, based upon assays in mammals. These include four disulfide bonds, an N-terminal LTCV sequence, a C-terminal TDKCNX sequence, the sequence CP(D/A)GQN(L/V)CFK in the region connecting loops I and II, and the sequence GC(A/V)ATCP between loops II and III.

**Figure 6:**
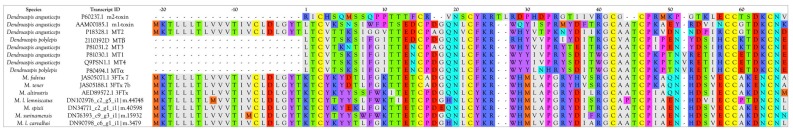
To date, all *Micrurus* venoms contain 3FTxs with significant similarities to muscarinic toxins from *Dendroaspis angusticeps* venom; however, these toxins also display numerous differences. It seems unlikely that these toxins could exhibit the same pharmacology in mammals as the mamba toxins, but they may antagonize mAChRs in reptiles, fishes, and onychophorans, the natural prey of coralsnakes. Aligned sequences of mamba muscarinic antagonists and putative muscarinic toxins from *Micrurus* venoms. Signal peptides are shown to the left of the black vertical line.

The six *Micrurus* transcriptomes were searched for muscarinic toxins 1–4. *Micrurus l. carvalhoi*, *M. corallinus*, *M. l. lemniscatus*, *M. s. spixii*, and *M. surinamensis*, as well as *M. altirostris*, *M. fulvius*, and *M. tener*, possess toxins with substantial similarity to Muscarinic Toxin 1 (MT1) from the venom of *Dendroaspis angusticeps* (Figure 6). However, of the attributes listed by Servent et al. [64], micrurine muscarinic-like toxins share only the disulfide pattern, but so do many toxins with entirely different pharmacologies. Moreover, there are also enough non-synonymous amino acid substitutions in the *Micrurus* sequences to raise doubts as to whether these really are muscarinic antagonists. Their 3D structures and surface hydrophobicities vary considerably. Even if they are muscarinic antagonists, Jolkkonen et al. [36] noted that minor differences in the amino acid sequences of the various muscarinic toxins from mamba venoms confer very different pharmacologies upon the toxins. In particular, micrurine toxins have 3-5 tyrosine and tryptophan residues in the N-terminal 11 residues that are not present in MT1 (Figure 6). On the other hand, the 21-residue MT1 signal peptide is identical to those of four *Micrurus* MTs (Figure 6).

All of the *Micrurus* species investigated here had an MT2-like sequence, except for *M. paraensis* (Figure 6); however, these are the same sequences that resemble MT1. Nonetheless, in order to align the micrurine sequences with MT2, it is not necessary to gap the sequences as when comparing them with MT1 (Figure 6). That of *M. corallinus* is 7 residues shorter than a normal MT2. All eight micrurine toxins share their cysteine positions with MT2, and all except the toxins from *M. fulvius* and *M. tener*, which have a P39A substitution, share P39 and P68 also. None of the micrurine toxins have MT2 P75. All except the *M. corallinus*, *M. fulvius*, and *M. tener* sequences share all of the acidic residues, except for the C-terminal aspartate, which oddly, only the *M. corallinus* toxin has. Furthermore, all *Micrurus* MT-like toxins have R48, and all but the odd toxin from *M. corallinus* also have F46, K47, W49, and H50. All micrurine toxins except for that of *M. corallinus* possess N43 and N72, and all of them have N85. All eight micrurine MT-like toxins (five from this study plus those from *M. altirostris*, *M. fulvius*, and *M. tener*) share the same 21-residue signal peptide with MT2, except that three of our coralsnake toxins have either of two valine to methionine substitutions (Figure 6). Alignments with *Dendroaspis angusticeps* muscarinic toxins MT3 and MT4 present the same sorts of identities seen with MT1 and MT2. 

da Silva et al. [65] isolated a 7048 Da protein from the venom of *M. l. lemniscatus*, which they dubbed MT-Mlα, because it displaced [^3^H]QNB from two types of binding site in rat hippocampus. It also antagonized accumulation of phosphate induced by carbachol, but with ~15-fold less potency than the M1 antagonist, pirenzipine. MT-Mlα [65] had the N-terminal sequence LICFICFSPTAH, which is unlike the sequences of any *Dendroaspis* muscarinic toxins. It also cannot be aligned with any sequence from our *M. l. lemniscatus* transcriptome. While we do not doubt their pharmacology, their sequence seems questionable. Only the 10-Cys 3FTxs have two cysteines separated by three residues (Figure 2 and Figure S2). While some of these have a tyrosine in position 4, none of them has a hydrophobic residue in position 5.

Until appropriate pharmacological assays are done with these toxins, we will not know whether they actually are muscarinic toxins, or whether they have some entirely different target. Given their interesting combination of identities and differences with the mamba toxins, it may be that these toxins are genuine muscarinic toxins, but adapted to incapacitate reptiles or fish, rather than mammals or birds. Mammalian muscarinic receptors are glycoproteins of 460–590 amino acids, depending upon the type. They show considerable sequence similarity from one mammal species to another (within types) [66]; however, BLAST searches of the NCBI *nr* database using the human M1 muscarinic receptor sequence as a query yielded identity scores with reptilian muscarinic receptors (M1–M5) of only 45–72%. Presumably glycosylation patterns may also differ between mammals and reptiles. While glycosylation apparently does not affect receptor function, toxins that target reptilian muscarinic receptors would be expected to differ significantly from those targeting mammalian receptors.

##### 3FTxs that Impact Glutamate Receptors

Montandon [67] investigated the neurotoxicity of *M. l. carvalhoi* venom using rat cerebrocortical synaptosomes. She concluded that it was primarily caused by liberation of l-glutamate by venom phospholipases A_2_, as a result of phospholipid hydrolysis and membrane disruption; however, she also determined that a 3FTx with a mass of 6675 Da was responsible for at least some of the neurotransmitter liberation. The action of this 3FTx appeared at least partially dependent upon the action of voltage-gated calcium channels, since it was inhibited when calcium was replaced with strontium.

Freire Donato investigated possible pharmacological targets responsible for neurotoxicity of crude *Micrurus l. carvalhoi* venom using an MTT viability assay in neonatal rat hippocampal neurons [68]. Neurotoxicity involved NMDA and nicotinic receptors. In addition, the venom, a reverse phase fraction thereof, and two purified 3FTxs liberated l-glutamate (Glu) from adult male rat cerebrocortical synaptosomes. Ca^2+^ is required for Glu liberation. Voltage-sensitive calcium channels (VSCCs) (Types N, P, and L) participated, and Glu release was reduced by 10 μM ω-conotoxin GVIA, 30 nM ω-agatoxin IVA, or 3 μM nifedipine; however, even when applied together, the three antagonists were unable to block all Glu liberation. Both NMDA and AMPA receptors were involved in neurotransmitter release, since it was abolished by combined application of the ionotropic glutamate receptor antagonists, MK-801 and CNQX (10 µM each), but not by either administered alone. Toxin Ml_7294_NTX (1 μM) released approximately 2.3 nmol Glu/mg of synaptosomal protein in 35 min. In comparison, 33 mM KCl liberated approximately 8 nmol/mg in the same period, indicating that the toxin is approximately 10^4^ times more potent than KCl on a molar basis. A possible mechanism of action is as follows. These toxins may activate pre-synaptic AMPA/kainate receptors, when the membrane is at rest. Upon activation, these receptors would depolarize the membrane, activating VSCCs (Types N, P, and L), and NMDA receptors. Subsequent entry of calcium via both calcium channels and NMDA receptors would result in exacerbated neurotransmitter liberation, as observed.

**Figure 7:**
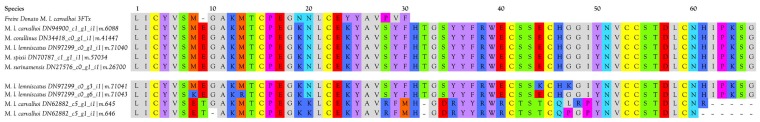
Freire Donato [68] determined the N-terminal amino acid sequence of a toxin from the venom of *M. l. carvalhoi* that provokes the release of glutamate from rat cortical synaptosomes. While that sequence contained two cysteine residues that appear spurious, when those are deleted, her sequence aligns well with sequences from five of the six *Micrurus* transcriptomes. These appear to represent the first complete sequences of snake toxins that provoke glutamate release.

Freire Donato determined that the N-terminal sequence of the 3FTx from *M. l. carvalhoi* venom responsible for glutamate release was: LICYVSM**C**GAKMTCP**C**EGNNLCEYYAVPVF. This sequence aligns modestly well with three of our sequences from the same taxon, except that it appears to contain two spurious cysteine residues (underlined) that have no equivalent in any other 3FTx sequences. However, if these are deleted, the alignment with our sequences is excellent (Figure 7). The 3FTx responsible for glutamate liberation from rat cerebrocortical synaptosomes appears to be a 66-residue, 8-Cys toxin. Identical sequences are found in all of our transcriptomes except for that of *M. paraensis*. These probably represent the first complete sequences of glutamate-liberating 3FTxs from any snake venom. Venoms of *M. l. carvalhoi* and *M. l. lemniscatus* each have two other sequences with very high similarity (Figure 7).

##### Cardiotoxins/Cytotoxins/β-Cardiotoxin

Based on electrophysiological evidence, Vital Brazil [69] concluded that cardiotoxins are present in *M. fulvius* venom. In a chromatographic survey of 11 *Micrurus* venoms, Silva Jr. et al. [70] found that molecules with the chromatographic behavior of *Naja kaouthia* cardiotoxin 1 were present only in the venoms of *M. albicinctus*, *M. Braziliensis*, and *M. frontalis*; however, they could not positively identify the toxins, which were most likely other 3FTxs. To date, no transcriptomic studies have provided any evidence for the presence of *Naja*-like cardiotoxins in any *Micrurus* venom, including even the deep transcriptomic study of *M. fulvius* venom glands [1]. BLAST searches of our transcriptomes produced no cardiotoxin/cytotoxin hits, and BLAST searches of the NCBI *nr* and *tsa_nr* databases, restricted to *Micrurus*, located only neurotoxin sequences. These “hits” resulted from the near identity of the signal peptide sequences. It seems most likely that the toxins responsible for the activity described by Vital Brazil are probably PLA_2_s, and we do not anticipate finding typical cardiotoxins/cytotoxins in *Micrurus* venoms.

β-cardiotoxin was discovered in the venom of *Ophiophagus hannah* a decade ago [71]. This 63-residue 3FTx binds to β1 and β2 adrenergic receptors to promote dose-dependent bradycardia, rather than the tachycardia that typifies the activity of conventional cardiotoxins. BLAST searches of our six transcriptomes and all *Micrurus* sequences from the NCBI *nr* database yielded no venom proteins with similarity greater than 50%. *Micrurus paraensis* yielded no hit at all. As of this writing, it seems probable that *Micrurus* venoms do not contain β-cardiotoxin-like proteins either.

##### Fasciculins

Kumar et al. [72] reported that *M. fulvius* venom possesses anti-cholinesterase activity comparable to that of various *Dendroaspis* and *Naja* venoms. This is difficult to interpret since *Dendroaspis* venoms possess fasciculins, which no *Naja* venoms are known to have and because the responsible component was retained by a 100 kDa-cutoff dialysis membrane, whereas a 7 kDa protein such as a fasciculin should have been lost. To date, no fasciculins have been reported in any *Micrurus* venom, and BLAST searches of our transcriptomes failed to produce even low-similarity hits. Possibly some *Micrurus* venoms contain an unrelated anti-cholinesterase protein, but this seems unlikely.

##### Calcicludine- or Calciseptine-like Toxins

Mamba venoms also contain potent antagonists of mammalian L-type calcium channels (calciseptine, calcicludine, FS2, etc.) [73,74]. However, BLAST searches of our transcriptomes and previously published toxin sequences from *Micrurus* venoms venoms failed to yield any candidates. We conclude that *Micrurus* venoms probably do not contain mamba-like, L-type calcium channel antagonists.

#### 2.2.4. 5′-Nucleotidase (5NUC)

5NUCs dephosphorylate purine mononucleotides to release free nucleosides (principally adenosine), which contribute to prey hypotension [46]. All eight *Micrurus* species examined to date have 5′-nucleotidases in their venoms (Figure S3). These are present at levels ranging from ~0–0.05% (Table S2). At 573–588 residues, with no signal peptides, micrurine venom 5NUCs are slightly shorter than their crotalid counterparts. Micrurine 5′-nucleotidases bear two different C-termini, one 14 residues longer than the other. Interestingly, the longer variant also appears in 5NUCs of the pitvipers, *Protobothrops elegans* and *Protobothrops flavoviridis* [11], and *Gloydius brevicaudus* (Ogawa and Yanoshita, unpublished, BAG82602.1. Micrurine 5NUCS are structurally almost invariant, in addition to their close identity with the crotalid enzymes (Figure S3).

#### 2.2.5. Acetylcholinesterase (AChE)

Micrurine acetylcholinesterases (AChEs) are modestly large proteins, containing approximately 585 amino acids. However, they are not expressed at biologically significant levels as they are in some Old World elapines. Based upon the hypertensive action of acetylcholine at M2 and M4 muscarinic receptors (Table 1), Aird [46] concluded that AChE might have a secondary role of promoting hypotension. That hypothesis now appears more unlikely, in light of subsequent research. Given that MT2 toxin, an M3 receptor agonist, comprises ~50% of the muscarinic toxins in *Dendroaspis angusticeps* venom, mambas seem to be targeting primarily vascular M3 muscarinic receptors, not only with MT2, but also with venom acetylcholine, to produce profound hypotension [47,75,76], an effect intensified by dendrotoxins, which liberate neurotransmitter [77], and fasciculins, which inhibit prey AChE so as to prevent transmitter degradation [78,79,80]. All of the foregoing implies that the primary function of AChE in the venoms of those elapids that produce it, is to starve neuromuscular junctions of transmitter, in concert with postsynaptic antagonists of nicotinic AChRs.

Among our *Micrurus* transcriptomes, AChE is most abundant in *M. l. lemniscatus* venom, where AChE transcripts total only 0.05% of the transcriptome (Table S2). Micrurine AChEs are highly variable in primary structure (Figure S4). Some, such as *M. s. spixii* transcript DN97541_c0_g1_i1|m.57626, are predicted to have 29-residue signal peptides, while others, such as *M. tener* AChE (JAS05178.1), are expected to have signal peptides of only 23 residues, with shorter N-termini.

#### 2.2.6. Aminopeptidases A and N (APA and APN)

Both APA and APN convert kallidin to bradykinin [81,82]. Cerebral microvascular aminopeptidase A inactivates the vasoconstrictive peptides, angiotensins I and II, by cleaving the amino-terminal Asp-Arg bond [83]. APN does not cleave mediators of hypotension and plasma extravasation, such as bradykinin and Substance P [84,85,86]. Thus, it is highly likely that the role of micrurine aminopeptidases A and N (M) is to promote hypotension, in concert with other venom constituents [46].

All of the transcriptomes except that of *M. surinamensis* had an APA sequence (Figure S5). The *Micrurus* sequences comprise 958 residues and are largely identical to the APA from *Gloydius brevicaudus* venom [87]. Because they are membrane-anchored proteins, they are predicted to lack signal peptides. *Micrurus s. spixii* had the highest APA transcript abundance, but that constituted only 0.09% of the transcriptome (Table S2). 

Aminopeptidase N (EC 3.4.11.2), also known as Aminopeptidase M [84] presented a similar picture. All of the transcriptomes except for that of *M. paraensis* contained an APN transcript, as did that of *M. fulvius* (Figure S6). All of our transcripts were incomplete, but they were nearly identical to the *M. fulvius* sequence, and all *Micrurus* sequences were very similar to the *Gloydius brevicaudus* sequence (BAG82599.1; Yuko Ogawa and Ryohei Yanoshita, 2007, unpublished). Among micrurine APNs, that of *M. surinamensis* was the most abundant at 0.01% of its transcriptome (Table S2).

#### 2.2.7. C-Type Lectin-like Proteins (CTLs)

Fry and Wüster [88] proposed that C-type lectin-like proteins were recruited into venoms twice. Galactose-binding lectins are widespread in venoms and appear to have been basal to the Colubroidea tree. However, heterotetrameric toxins that lack mannose-binding capacity [89,90], seem to have been a strictly viperid invention. Viperid CTLs are all multimeric proteins composed of heterodimers that act upon platelets [91]. Some of them inhibit platelet aggregation, while others agglutinate, or aggregate and activate platelets. Viperid CTLs often bind to more than one type of platelet receptor [91]. However, with the discovery of bungarine, micrurine, and acanthophiine lectins [92,93], it is clear that recruitment of C-type lectin-like proteins is more complicated than Fry and Wüster suggested.

CTLs have been reported previously in *M. corallinus, M. fulvius*, and *M. tener* venoms [1,2,94], although there have been no pharmacological characterizations to date, nor any assessment of structural variability within this toxin family. Micrurine CTL transcripts encode proteins of 149–150 amino acids, with putative signal peptides of 20 residues (Figure S7). These New World elapid CTLs exhibit tremendous sequence variation, and they may comprise 8–10 structural subclasses. CTLs from *Micrurus* venoms may have 6, 7, 8, or 9 Cys residues, while viperid CTLs normally have 8. This situation raises the possibility of heterodimeric toxins, similar to those of viperid venoms, but these toxins could also be complexed with a non-CTL moiety, or the extra Cys could simply be free. Regardless, the Cys patterns suggest a minimum of 3–4 structural subclasses, though whether they differ pharmacologically cannot be surmised at present. Nine toxins also bear C-terminal extensions of 2–6 amino acids (Figure S7). However, of the 40 sequences presented here, 31 bear an EPN sequence (residues 113–115) that is characteristic of mannose binding lectins [95] (Figure S7). In addition, three micrurine CTLs bear a KPS sequence in these positions. Five possess an ATS sequence, and one *M. l. lemniscatus* toxin has a QPD sequence, indicating that the latter may be a galactose-binding lectin (GBL) [95]. Nonetheless, its Cys pattern is different from those of putative micrurine GBLs, discussed below. Among our samples, CTLs ranged in abundance from trace quantities in *M. s. spixii* to 1.87% in *M. paraensis* and 3.35% in *M. corallinus*. These significant quantities suggest a CTL role of some importance, at least in the latter two species.

#### 2.2.8. Cysteine-Rich Secretory Proteins (CRISPs)

CRISPs are apparently not abundant components of any snake venoms, but they are widely distributed taxonomically [96]. Interestingly, they have also apparently been recruited into all varanid lizard venoms, in which they constitute the second most abundant venom constituent (Ivan Koludarov, personal communication). The CRISPs, ablomin (*Gloydius blomhoffii*), triflin (*Protobothrops flavoviridis*), and latisemin (*Laticauda semifasciata*) are L-type Ca^2+^ channel antagonists of depolarization-induced arterial smooth muscle contraction [97]; thus, they promote vasodilation and hypotension. Patagonin, a CRISP isolated from the venom of the colubrid, *Philodryas patagoniensis*, damaged murine skeletal muscle [98].

All six *Micrurus* transcriptomes contained CRISP sequences of 238–239 residues (Figure S8). The sequences from *M. l. carvalhoi*, *M. l. lemniscatus*, and *M. surinamensis* are identical over the first 201 residues, at which point they terminate. We do not know whether they are complete or truncated. The sequences of *M. corallinus* and *M. s. spixii* differ somewhat from the three foregoing, but that of *M. paraensis* is radically different, being more similar to the CRISP from *Ovophis okinavensis* venom [10]. Micrurine CRISPs range from trace levels in most species to 0.24% of the *M. l. lemniscatus* transcriptome (Table S2).

#### 2.2.9. Cysteine-Rich Secretory Proteins with EGF Domains (CRISP-EGF)

All of the *Micrurus* transcriptomes possessed from two to five CRISP-EGF sequences. These are very minor venom components, ranging from trace quantities to a maximum of 0.13% in *M. surinamensis* venom. Snake venom CRISP-EGF transcripts apparently encode proteins of 362 amino acids with 28-residue signal peptides. Structurally they appear to comprise three subclasses, one of which includes CRISP-EGF transcripts from *Micrurus fulvius* and from crotalid venoms and colubrid tissues [1,10,99] (Figure S9).

The second and third subclasses consist only of micrurine sequences from the present study. The second subclass is most closely related to an uncharacterized protein from the *Protobothrops mucrosquamatus* genome (XP_015675368) and a CRISP with an EGF domain from the *Thamnophis sirtalis* genome (XP_013930246.1) (Figure S9). Both of these latter proteins exceed 960 amino acids, whereas the micrurine sequences appear to contain fewer than 400 residues, although we cannot be absolutely certain of this since we did not isolate a stop codon. However, all of the subgroup 2 sequences terminate with the same EIGCV sequence, which is very unlikely if they are all incomplete.

Unfortunately, the third subclass is represented only by two pairs of incomplete sequences. The first pair (*M. surinamensis* DN51727_c0_g2_i1_m.16805 and *M. paraensis* DN13321_c0_g1_i1|m.25776), is most closely related to *Protobothrops flavoviridis* fibulin-1-like protein (XP_015686848.1; 97% identity over the 90-residue *M. surinamensis* sequence). The second pair of sequences (*M. s. spixii* DN99876_c0_g3_i1|m.54981 and *M. surinamensis* DN81846_c0_g1_i1|m.38354) is most closely related to *Protobothrops mucrosquamatus* fibulin-1 (XP_015670949.1; 92% identity over the 94-residue *M. surinamensis* sequence) (Figure S9). All four of these are expressed at essentially trace levels (<0.0002%), so they may be contaminating tissue proteins. Human fibulins include Cys-rich, Ca^2+^-binding, extracellular matrix and plasma glycoproteins of 566-683 amino acids [100]. Epidermal growth factor [101] and extracellular matrix proteins containing EGF domains [102] possess a six-Cys motif that is repeated nine times in fibulins [101]. According to Tran et al. [103], fibulin-1 is the major fibulin in human blood, occurring at a concentration of 30–40 μg/mL, 1000× the concentration of fibulin-2. Tran et al. found that this 340-kDa polypeptide consistently co-purified with fibrinogen. Fibulin-1 is incorporated into clots in vitro and has been detected in thrombi in vivo. In the presence of fibrinogen, platelets bind to human fibulin-1 [104]. Accordingly, there is a conceivable role for fibulin-like proteins in envenomation.

The aggregating proteoglycans (aggrecan, versican, neurocan, and brevican) are important components of many extracellular matrices [105]. Their N-terminal globular domains bind hyaluronan and their C-termini contain C-type lectin domains. In the presence of Ca^2+^, the lectin domains of aggrecan and versican bind to the Ca^2+^-binding EGF repeats of fibulin-1, with K_D_s in the low nM range [105]. Versican is highly expressed in arteries, veins, and capillaries [106]. Perhaps, given the capacity of fibulin-1 to bind fibrinogen and platelets, *Micrurus* venom fibulin homologs promote clot formation, their smaller size notwithstanding. Possibly, fibulin-1-like proteins complement the actions of prey serine proteases. Eagle [106] found that a mixture of unspecified *Micrurus* venoms was capable of weakly clotting fresh, citrated horse plasma, due to apparent prothrombin activation, and not to direct cleavage of fibrinogen. Crotalid thrombin-like enzymes promote formation of small clots that stimulate prey anticoagulation cascades to destroy the clots, with the result that prey blood is quickly rendered incoagulable. However, serine proteases are minor components of micrurine venoms, and to date, there is no evidence that they are thrombin-like. It also remains to be seen whether micrurine fibulin-1-like proteins bind and activate platelets. Perhaps, given that they are ~40% smaller than human fibulin-1, in concert with prey serine proteases, they promote defective clot formation so as to render the prey’s blood incoagulable, but given their expression levels and their probable non-enzymatic character, they probably contribute little to envenomation.

#### 2.2.10. Cystatin (CYST)

Cystatins have not been reported previously as snake venom constituents and we make no claim that they are. Cystatins participate in cell survival, proliferation, and differentiation, cell signaling, and immunomodulation [107], roles that do not seem as pertinent to envenomation as their well-known capacity to inhibit cysteine proteases. However, they are present in the *Micrurus* venoms examined here in only trace quantities (0–0.02% of the transcriptomes). These transcripts encode 136-residue proteins with 24-residue signal peptides and three cysteine residues; thus, the mature proteins are slightly smaller than phospholipases A_2_. Mammalian cystatin families 1 and 2 contain proteins of 100–120 amino acid residues. Those in family 1 are synthesized without signal peptides or disulfide bonds, and are normally intracellular. Those in family 2 have signal peptides and disulfide bonds, and are secreted proteins [108].

We isolated cystatin transcripts from all six species (Figure S10); however, no cystatin sequences have been reported from any other *Micrurus* species to date. The six cystatin sequences presented here are largely invariant, owing to their low expression levels. There are only 7 variable positions in the 112-residue proteins. Their cysteine residues occur in the same positions as do mammalian Family 2 inhibitors, with which they share only 45–55% sequence similarity. However, it seems probable that these are secreted proteins, as venom proteins have to be, unless they are secreted in microsomes [109].

#### 2.2.11. Dipeptidyl Peptidase IV (DPP IV)

DPP IV was first discovered in snake venoms by Silva Jr. and Aird [110] in a study of coralsnake venom lethality. High levels of DPP IV are found in blood capillaries [111,112], suggesting that its function is to control hypertension, because of its ability to catabolize Substance P [113], neuropeptide Y, and peptide YY [114,115]. Aird [46] offered a hypothetical explanation for the presence of DPP IV in venoms, suggesting that its role was to counteract a hypertensive response on the part of envenomated prey by destroying hypertensive peptidyl hormones released to offset venom-induced hypotension.

Silva Jr. and Aird [110] found DPP-IV activity in all venoms assayed (16 *Micrurus*, *Bungarus multicinctus*, *Naja naja*, and *Bothrops moojeni*), except for those of *M. albicinctus* and *M. l. carvalhoi*. It was subsequently found in a broad taxonomic array of venoms [116] and DPP IV transcripts have now been reported in various crotalid, viperid, and elapid venoms [10,11,117,118]. All six transcriptomes contained DPP IV transcripts, and ironically, given its generally low enzyme titers, *M. surinamensis* had two. One of these contained a 26-residue insert not seen in any other DPP IV transcript reported to date (Figure S11). This variant is remarkable given the generally low variability seen in snake venom DPP IVs. Even crotalid DPP IVs vary relatively little from micrurine sequences; however, DPP IV is a trace level component in all six venoms examined here.

#### 2.2.12. Galactose-Binding Lectins (GBL)

Galactose-binding lectins, a subset of C-type lectins, were first reported by Gartner et al. [119] from the venom of *Bothrops jararaca*. These Ca^2+^-dependent lectins induce platelet aggregation [9,120] and are potent mitogens [121,122]. In all previous studies, GBLs have been found at very low levels [120], and their known sequences display very little sequence variation [10]. Accordingly, Aird et al. [10] postulated that given their mitogenic properties, the primary function of GBLs may be to upregulate venom synthesis in the venom gland when venom supplies become depleted. However, with the present findings, that view may have to be revised somewhat. While several species in the present study also have very low GBL titers (0–0.19%) (Table S2), *M. paraensis* (1.93%) and *M. corallinus* (3.35%) have by far the highest levels reported to date, suggesting that in these two species at least, GBL hemagglutinating and edematogenic properties [122] may play a significant role in envenomation.

Moreover, the collection of GBL sequences yielded by this study presents a picture of greater sequence diversity than hitherto seen (Figure S12). *Micrurus* GBLs comprise two groups, each having 158 residues, including a predicted 23-residue signal peptide. One group commences with an unusual CCC sequence shared with various Australian elapids, while the other group has a YTC sequence. The former group has 10 Cys residues while the latter group has 8, presumably arranged in disulfide bonds. While the two groups are generally similar, displaying the same invariant and variable positions, several other positions appear to differ consistently. At position 33 (Figure S12), all but one of the CCC toxins have proline, while all of the YTC toxins have serine. At position 58, the CCC toxins have phenylalanine, histidine or leucine, in order of abundance, whereas the YTC toxins all have histidine. Lastly, at position 81, all the CCC toxins have serine or threonine, whereas all the YTC toxins have serine or phenylalanine (Figure S12). The significance of these differences, if any, is unknown.

**Figure 8:**
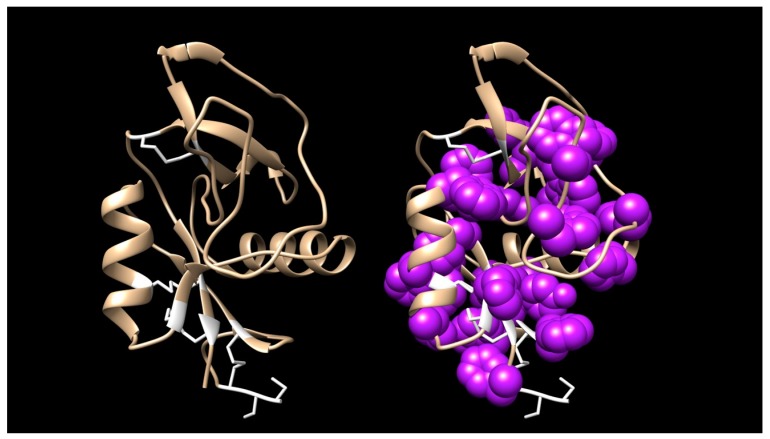
The galactose-binding lectin from *Micrurus corallinus* venom (DN79899_c0_g1_i1|m30755) showing the molecule’s secondary structure (left) and the same model with the aromatic residues shown in a space-filling representation. Aromatic residues tend to be clustered in the core, but most tyrosines have their hydroxyl groups exposed on the surface and several of the tryptophans are also partially exposed. The functional reason for this extremely high aromatic content is unknown. The model was made with SWISS-MODEL using a GBL from *Protobothrops mucrosquamatus* venom as a template. The model was then further refined and energy minimization was performed using Chimera [21].

Of particular interest is the large number of invariant aromatic residues, including W7, Y15, W24, Y55, Y59, W67, W79, W81, F/Y89, W92, Y115, W118, and F/Y129 (numbering as per Figure S12). In addition, 3 other positions appear overwhelming aromatic with only occasional non-aromatic substitutions at F13, F18, F/Y30, and F/Y135. Many GBLs also have additional aromatic residues that do not appear in all homologs, e.g., F52(I,L), Y57/S, F/Y77(R/G), Y/F100(L/S), W101(Q/N/K/R), W110(V/L), F112(L/V/A/N/S), and F127(R/N) (Figure S12). While phenylalanine and tyrosine are interchangeable at several of these apparently obligatory aromatic positions, tryptophan is never substituted, suggesting structural, as well as chemical roles. We are unaware of any other toxin family with such a high aromatic amino acid content, an attribute that fairly well begs for a structural/functional investigation (Figure 8).

#### 2.2.13. Guanylyl Cyclase (QC)

Guanylyl cyclase is believed to serve only the function of cyclizing N-terminal glutamine residues of specific venom proteins to prevent their rapid degradation in host tissues. The acidic subunit of crotoxin, from the venom of *Crotalus durissus terrificus*, consists of three chains derived from an acidic phospholipase A_2_ [123]. The B and C chains are blocked with pyroglutamate [123,124]. Thus, GC is not a snake venom constituent in the usual sense, although it is usually present in trace quantities. All six coralsnake transcriptomes possess identical 368-residue transcripts with putative 23-residue signal peptides that comprise from 0–0.02% of their respective transcriptomes. Even the GC transcript of the habu, *Protobothrops flavoviridis*, differs from the micrurine GCs at only 5 residues (Figure S13).

#### 2.2.14. Hyaluronidase (HYAL)

Hyaluronidase is generally a minor enzymatic component of snake venoms. Venom hyaluronidase has been deemed a “spreading factor” because its degradation of extracellular matrix enables venom hydrolases and non-enzymatic constituents to attack additional targets [125,126]. As such, hyaluronidase probably serves primarily to digest the prey. HYALs have been recorded to date in 10 *Micrurus* venoms (Figure S14), including all six of the species we studied. Micrurine hyaluronidases comprise 465 amino acids and lack signal peptides. In our transcriptomes, hyaluronidases accounted for between ~0% (*M. s. spixii* and *M. l. lemniscatus*) and 0.27% (*M. surinamensis*) of the transcriptomes. Consistent with the finding of Aird et al. [11] that the most abundant venom proteins evolve most rapidly, hyaluronidases show strikingly little sequence variation. Most *Micrurus* species appear to have only one hyaluronidase transcript, but *M. l. carvalhoi* has three (Figure S14).

#### 2.2.15. Kunitz Serine Protease Inhibitors (KSPI)

Kunitz serine protease inhibitors have been recognized for decades as a family of short, structurally similar toxins (59–61 residues with three disulfides) with diverse functions ranging from non-neurotoxic inhibitors of trypsin or chymotrypsin to neurotoxic ligands of potassium and calcium channels, such as the dendrotoxins [74,127,128,129,130,131]. Dufton [132] noted that relatively few amino acid substitutions are necessary to turn a protease inhibitor into a dendrotoxin. The present study confirms an earlier sequence from *M. tener* and shows that KSPIs also include a handful of homologous, 232-residue toxins, in several *Micrurus* venoms (Figure S15). The latter should not be confused with SERPINs, which are nearly twice the size of the long KSPIs.

These long KSPIs have atypical, 20-residue signal peptides commencing with MRREKS (Figures S15 and S16), instead of the usual 24-residue signal peptide commencing with MSSGGL [127]. Long KSPIs have an invariant, acidic N-terminus, ESPPD (Figures S15 and S16). Their N-termini show considerable homology to typical, short KSPIs, with a strongly aromatic (WWY) sequence (residues 48–50) and six cysteines in their usual locations. Four hydrophilic residues (NQNN, NENN, NKNN, or NANN) are found at positions 68–71 (Figures S15 and S16). Typically, after the last cysteine, KSPIs terminate with two or three residues, one of which is aliphatic.

**Figure 9:**
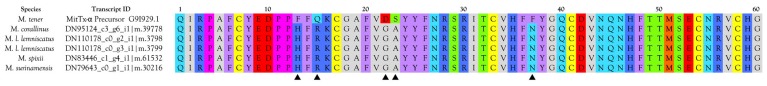
Four of the species examined here have sequences that are strikingly similar to the α subunit of the nociceptive toxin (MitTx) from *Micrurus tener* venom, which even the closely related *M. fulvius* lacks. Nonetheless, the South American *Micrurus* toxins have five substitutions (indicated by triangles), four of which are strongly non-synonymous. Signal peptides 24 residues long were essentially invariant (MSSGG LLLLL GLLTL CAELT PVSS). Only *M. l. lemniscatus* toxin DN110178 had a G/R substitution in position 5.

In contrast, the long KSPIs continue with an 11-residue span that is largely aliphatic, mimicking a signal peptide (Figure S16). The next 40 residues contain 6 prolines and two cysteines. The two cysteines are nine residues apart, as is the first pair of cysteines in the short KSPIs. Positions 139–141 show another aromatic triplet (WYF), like that mentioned above. The next two pairs of cysteines are separated by 8 and 5 residues, exactly as their counterparts in the short toxins. The long KSPIs also have a block of hydrophilic residues (positions 159–162: NKNN), mimicking a similar block in their own N-termini and in the short toxins (positions 68–71) (N*NN, in which the asterisk may be Q, R, K, D, E, or A) (Figure S16). Because such a variety of sequence segments align perfectly with the N-termini of the short toxins, it appears that the long KSPIs were formed partly due to a gene duplication, in which a short toxin was duplicated and grafted onto its own C-terminus (Figure 9). There is even some suggestion of a second duplication, with what appears to be another pseudo-signal peptide (residues 109–125); however, in the second putative duplication, sequence homology degrades very significantly (Figure S16).

Because the N-termini of these toxins show great similarity to the short toxins, it seems probable that these long toxins are functional KSPIs; however, their specificity is unknown. It is likely that the long KSPIs do not target them same prey proteins as the short toxins. Their presence in eight *Micrurus* species may offer a clue as to they prey specificity. On the other hand, it is difficult to argue strongly for any great importance in envenomation in view of their expression levels: *M. surinamensis*, 0.006% of the transcriptome; *M. s. spixii*, 0.007%; *M. paraensis*, 0.0003%. As a percentage of the total KSPI level in these venoms, these toxins represent: *M. surinamensis*, 0.65% of the transcriptome: *M. s. spixii*, 1.1%; *M. paraensis*, 0.04%. If long KSPIs are non-enzymatic, that means they have stoichiometric relationships with their targets. If so, their contribution to envenomation is presumably minor, but that still begs the question of their function, a question that pertains to most snake venom KSPIs. Beyond the six cysteines, they share very few residues with α-dendrotoxin; thus, they probably do not block K^+^ channels, unless the differences reflect differences in mammalian and reptilian K^+^ channels.

#### 2.2.16. L-Amino Acid Oxidase (LAO)

The flavin-enzyme, L-amino acid oxidase (LAO), is responsible for the bright yellow color of many solenoglyph venoms, in which LAO content can approach 10% [10]. It is a more minor constituent of *Micrurus* venoms, ranging from essentially 0% (*M. s. spixii* venom) to 0.84% (*M. corallinus*). Snake venom L-amino acid oxidase (EC 1.4.3.2) generates H_2_O_2_ from the conversion of L-amino acids to keto acids. While anticoagulant and apoptotic roles have been demonstrated for LAO, Aird [46] argued that LAO’s nearly ubiquitous distribution in snake venoms implies an important role in envenomation and suggested that LAO’s most important function is to promote hypotension by augmenting nitric oxide production via stimulation of soluble guanylate cyclase. Micrurine LAOs are large proteins, with 499 residues and predicted 18-residue signal peptides (Figure S17). They exhibit relatively little sequence variation, except at specific residues, where the same sorts of amino acid substitutions are encountered across species.

#### 2.2.17. Metalloproteases, SVMP Type III (P-III)

Snake venom metalloproteases (SVMPs) are classified according to their domain organization [133,134,135]. P-I SVMPs are the simplest class of enzymes, containing only a metalloproteinase catalytic domain. P-II SVMPs contain a metalloproteinase domain linked to a disintegrin domain. P-III SVMPs are the most structurally complex, containing catalytic, disintegrin-like, and cysteine-rich domains [133]. Many venoms contain multiple metalloprotease forms; however, elapid venoms tend to be depauperate in SVMPs relative to viperid venoms (~70% less than viperid venoms) [136,137]. All P-III SVMPs are assumed to be catalytically active. The majority of these cause hemorrhage, but some specifically inhibit platelet aggregation, cleave von Willebrand factor, or activate prothrombin or Factor X [135].

*Micrurus* venoms appear to contain predominantly one Type III metalloprotease, comprising 614 residues, including a 20-residue signal peptide (Figure S18). One *M. l. lemniscatus* transcript (lemniscatus DN101949_c32_g1_i4|m.13925) yields a mature protein with a mass of 66,657 D and a predicted pI of 8.37. Except for other micrurine MPs, this protein is most similar to atragin, from the venom of *Naja atra* [138]. The metalloprotease content of the venoms we examined varies from essentially 0% in *M. s. spixii* to 2.1% in *M. corallinus*. The latter suggests a significant function in envenomation. Nonetheless, the pharmacological and physiological effects provoked by micrurine metalloproteases are unknown.

#### 2.2.18. Natriuretic Peptides (NP)

Atrial and brain natriuretic peptides had been known for several years when C-type natriuretic peptide was discovered in porcine brain by Sudoh et al. [138]. Both atrial and brain natriuretic peptides resemble ribbons, folded across themselves to form a loop, pinned with a single disulfide bond and having two tails [138]. C-type natriuretic peptides terminate with the second cysteine so that they have only a single tail. Nonetheless, their pharmacological spectrum is similar to those of atrial and brain natriuretic peptides [138].

The first snake venom natriuretic peptide was discovered two years later in the venom of the eastern green mamba (*Dendroaspis angusticeps*) (DNP) [139]. Natriuretic peptides have subsequently been discovered in a variety of elapid and crotalid venoms [95,140,141,142,143].

Natriuretic peptide transcripts were found in all six of the *Micrurus* species we investigated. They have also been reported from *M. fulvius* and *M. altirostris*. Like ANP and BNP, micrurine NPs have 25-residue signal peptides (Figure S19). After removal of the signal peptide, another 61 residues must also be cleaved from the N-terminus, assuming an N-terminal tail length equivalent to that of DNP, bearing an invariant glutamate residue. Since human CNP lacks a C-terminal tail, it is unclear whether these micrurine peptides have tails, and if so, how long they are. One of the *M. surinamensis* NPs bears what appears to be a premature stop codon in position 134 (Figure S19). That transcript may be a misassembly or a pseudogene, since it also has an extra cysteine not seen in any other transcript. On the other hand, even truncated at that point, it still has 8 residues beyond the C-terminus of DNP.

No other NP transcript bears a stop codon, even though 2 *M. l. carvalhoi* transcripts are 195 and 267 residues in length. These two transcripts encode one and two additional NPs, respectively, in a manner resembling that of bradykinin-potentiating peptides in crotalid venoms [140,144] (Figure S19). Thus, it is impossible to say with absolute certainty how long these transcripts should be, or the natriuretic peptides they encode. Nonetheless, in *M. corallinus* and *M. s. spixii* venoms we repeatedly isolated a peptide -DRIGNVSGMGCNHVRT-, which corresponds to residues 73–89 (Figure S19). Interestingly, this peptide spans the first and second NP sequence repeats.

Immediately upstream from the N-terminal glutamate residue, most NPs display an -GPAK- or -GLAK- sequence. One group of three NPs from venoms of *M. l. carvalhoi*, *M. corallinus*, and *M. l. lemniscatus*, deviates sharply from this pattern, having a -GLDT- sequence. The mature NP sequences are likewise very unusual. Between the cysteines, two of the hydrophilic residues have been replaced with hydrophobic aliphatic residues (Q95L, D98V). The nearly invariant M106 has also been replaced with L. Functionally the effect of these changes is unknown, but these toxins should be much less hydrophilic than most NPs.

#### 2.2.19. Nerve Growth Factor (NGF)

Micrurine NGF transcripts encode 244 amino acids plus 18-residue signal peptides (Figure S20). They are highly similar sequences, displaying numerous blocks of invariant residues. Most of the variable positions show specific sets of substitutions, synonymous and non-synonymous, that occur without regard to species. The only strongly divergent sequences include a set of six transcripts from *M. l. lemniscatus*, which vary at numerous positions. Another group of four transcripts from *M. l. lemniscatus* and *M. corallinus* are also significantly different from all others; however, the significance of these variations, if any, is unknown (Figure S20). NGFs function as arginine esterases, so they probably contribute to venom hypotensive activity via nitric oxide liberation and histamine release [14,15,46,145,146]. Mouse salivary NGFs activate plasminogen, their only known action upon a biologically important, non-neural substrate [147,148], but it is not clear whether snake venom NGFs can also do this. If so, they would hinder blood clotting, consistent with envenomation strategies [46].

#### 2.2.20. Nociceptive Toxins (NOCI)

Bohlen et al. [149] reported a non-covalent, heterodimeric toxin (MitTx) from the venom of *M. tener* that specifically targets ASIC1 acid-sensing ion channels to induce intense, persistent pain. The toxin consists of a Kunitz serine protease inhibitor homolog and a non-catalytic phospholipase. The latter lacks the essential active site histidine and aspartate residues. Interestingly, *M. tener* has a series of PLA_2_ toxins with this same modification.

Four of the six Brazilian coralsnakes we examined (except *M. l. carvalhoi* and *M. paraensis*) have sequences that are very similar to MitTxα, the KSPI subunit, but there are no other sequences in the NCBI *nr* database that provide a match of better than ~52%. Even *M. fulvius* does not have an equivalent toxin (Figure 9). MitTxα homologs have predicted 24-residue signal peptides, leaving 60-residue mature toxins. The Brazilian sequences differ from the MitTxα sequence at five positions, but only one of the substitutions (Q15R) is reasonably conservative (Figure 9). The others include F13H, D22G, S23A, and F37N. Accordingly, it is impossible to say whether these toxins are actually functional MitTxα homologs.

**Figure 10:**
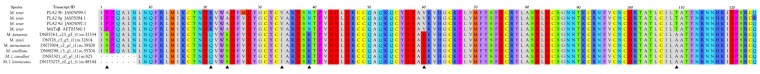
All six *Micrurus* species examined produced a close homolog of MitTxβ, the nociceptive toxin β subunit from *Micrurus tener*, including *M. paraensis* and *M. l. carvalhoi*, which apparently do not produce the α-subunit. The South American *Micrurus* sequences are basically identical, and differ from that of MitTxβ at only six positions (indicated by triangles). The three M. tener PLA_2_ sequences had 21-residue signal peptides while MitTxβ and putative homologs had 24-residue signal peptides. 21-residue signal peptides had the sequence MNPAH LLVLA AVCVS LLGASS. MitTxβ and putative homologs had a three-residue N-terminal extension, MDK.

For MitTxβ, SignalP also predicts a 24-residue signal peptide, resulting in a 125-residue mature protein. All six Brazilian species also possess toxins that differ at only 7 positions from *M. tener* MitTxβ (Figure 10). These include P2S, R21Q, A24S, V34I, N39K, V60E, and T109A, numbering from the start of the mature protein; however, only the substitutions at positions 21, 34 and 39 are reasonably conservative. Nonetheless, all of the Brazilian *Micrurus* proteins are also non-catalytic. Like MitTxβ they possess an unusual QAQKQ sequence between the fifth and sixth cysteine residues that is not shared by any other *Micrurus* PLA_2_s. *Micrurus tener* has at least two other PLA_2_s that are nearly identical to MitTxβ, with conservative substitutions at V27M and Q125R. Without pharmacological studies, it is impossible to say whether all of these toxins are actually nociceptive, like MitTx; however, their common structural similarities and their distinctness from all related toxins, particularly micrurine PLA_2_s, suggest that they probably are.

In summary, it is impossible to say at present whether the South American species examined here possess functional equivalents to MitTx, and if so, which subunit arose first. It seems probable that *M. l. carvalhoi* and *M. paraensis* do not have them, given the apparent lack of an MitTxα sequence, but perhaps these species employ another KSPI in this role. Or perhaps these South American MitTx homologs have other functions, as yet undiscovered.

#### 2.2.21. Phosphodiesterase (PDE)

Snake venom phosphodiesterase contributes to prey hypotension by liberating purine nucleotides (especially ATP) from prey nucleic acids. The nucleotides can be subsequently dephosphorylated by venom and prey 5′-nucleotidases to release free nucleosides. Among nucleosides, adenosine plays an especially significant role in envenomation [46]. All of the *Micrurus* venoms investigated here apparently have a single phosphodiesterase gene (850–852 amino acids), except for *M. surinamensis*, which, barring a transcript misassembly, may have two (Figure S21). The transcript in question shows an inserted glycine at position 700, and a glutamate residue at position 698, where all other sequences have leucine. Signal P predicts signal peptides of 18 residues, but they are probably actually 22 residues long, given the unbroken 14-residue run of aliphatic amino acids. PDE sequences are highly conserved, given the similarity of *Micrurus* sequences to *Protobothrops* sequences (Old World crotalids) (Figure S21). PDEs are expressed at trace levels in five of the *Micrurus* species and at 0.02% in *M. corallinus*. Their low variability and low expression levels apparently provide additional support for the principle that the most abundant venom proteins evolve most rapidly [11].

#### 2.2.22. Phospholipases A_2_ (PLA_2_)

##### Overview and Diversity of PLA_2_s

Kocholaty et al. [150] found that *Micrurus fulvius* venom exhibited higher PLA_2_ activity than venoms of several species of *Bungarus* and *Naja*, and *Ophiophagus hannah*. Ramsey et al. [151] fractionated *M. fulvius* venom on CM-Cellulose and found PLA_2_ activity in every fraction. Possani et al. [152] fractionated the venom of *M. fulvius microgalbineus* in the process of purifying a PLA_2_, the first *Micrurus* toxin to be isolated and characterized from any species. Their gel filtration profile of the crude venom on Sephadex G-50 and subsequent subfractionation of the main peak by cation exchange chromatography also revealed PLA_2_ activity in every fraction, suggesting that *M. fulvius* venom is preponderantly PLA_2_. This was confirmed when Margres et al. [71] produced the first coralsnake venom gland transcriptome and found that PLA_2_ transcripts comprised 63.4% of the transcriptome. By proteomic means, Vergara et al. [153] obtained similar results. Not only is *M. fulvius* venom composed largely of PLA_2_, but 54 of the 63 complete *Micrurus* PLA_2_ sequences in the public domain come from that species. Likewise, the *M. nigrocinctus* proteome appears to be largely PLA_2_s (48.0%) [154].

However, venoms of other coralsnakes are not so dominated by PLA_2_s. Both *M. surinamensis* (Letícia, Colombia) [12,155] and *M. l. carvalhoi* venoms [155] show relatively little PLA_2_ activity. However, activity does not necessarily reflect the amount of PLA_2_ present. Silva Jr. and Aird [70] reported that in *M. f. fulvius* venom, the enzymatic activity level was much lower than expected, relative to the amount of PLA_2_ present. At that time, non-neurotoxic, non-catalytic, myotoxic PLA_2_s were known [156,157,158], but only later did it become clear just how much variation in catalytic activity exists among catalytic phospholipases [159]. For instance, Kopper et al. [160] reported an 8.9-fold variation in PLA_2_ activity/mg of venom protein among 13 specimens of *M. tener*, suggesting that interspecific comparisons based on pooled samples may be more reliable than those based on single specimens.

*Micrurus fulvius* venom caused direct and irreversible cardiac depression in cats, in addition to respiratory insufficiency, but exerted little effect on conductivity of rat phrenic nerve [161]. Myoglobin was not released despite large amounts of free hemoglobin; hence the authors concluded that *M. fulvius* PLA_2_s act like cardiotoxins. Myotoxic, catalytic PLA_2_s had been known from crotalines for at least 9 years [162,163]; however, myotoxic elapid PLA_2_s were not reported until 1975 [164]. The first non-catalytic, myotoxic PLA_2_ would not be discovered until 1984 [156,157,158] in the venom of the pit viper, *Agkistrodon piscivorus*.

Assays for phospholipase activity depend heavily upon the types of phospholipases present, the phospholipid substrate used, the assay temperature, the organizational state of the phospholipids, the presence or absence of detergents, and other factors [165,166,167]. Aird and Silva Jr. [12] detected PLA_2_ activity against phosphatidylcholine in venoms of 11 coralsnake taxa. Echoing the earlier finding of Kocholaty [150], all but *M. surinamensis* venom exhibited higher PLA_2_ activity than the two outgroup species, *Bothrops moojeni* and *Naja kaouthia*; however, such data provide little indication of what types of PLA_2_ are present. Moreover, as Silva Jr. et al. [70] discovered, in some cases PLA_2_ activity correlated well with PLA_2_ content as revealed by chromatographic profiles (*M. surinamensis* and *M. l. lemniscatus*, the least and most active samples, respectively), but in other cases it did not. Chromatographic profiles suggested very high PLA_2_ content in both *M. fulvius* and *Naja kaouthia* venoms, but both were relatively inactive in the phosphatidylcholine assay system. Undoubtedly, the activity differences reflect the diversity of pharmacological functions of different PLA_2_s. de Roodt et al. [168] reported that myotoxicity was present only in *Micrurus* venoms with the highest PLA_2_ activity (*M. fulvius*, *M. nigrocinctus*, *M. pyrrhocryptus*), but that all six venoms examined, including additionally *M. altirostris*, *M. baliocoryphus*, and *M. surinamensis* venoms, manifested PLA_2_ activity. Olamendi-Portugal et al. [17] detected peptides corresponding to PLA_2_s in *M. surinamensis* venom, but the pharmacological types could not be determined from the short sequences available.

Because they are not confounded by assay conditions and variations in catalytic rate, etc., transcriptomic studies promise to resolve at least the questions regarding compositional differences between venoms. High-throughput techniques (e.g., Illumina technology) [71] yield much more quantitative data than was possible with earlier molecular biological techniques [169]. Technological advances are occurring at such a rapid rate that current Illumina technology produces roughly 5 − 10*x* the number of reads as in the Margres study [1], and very soon, the current technology will likely be supplanted by technologies offering vastly longer reads.

**Figure 11:**
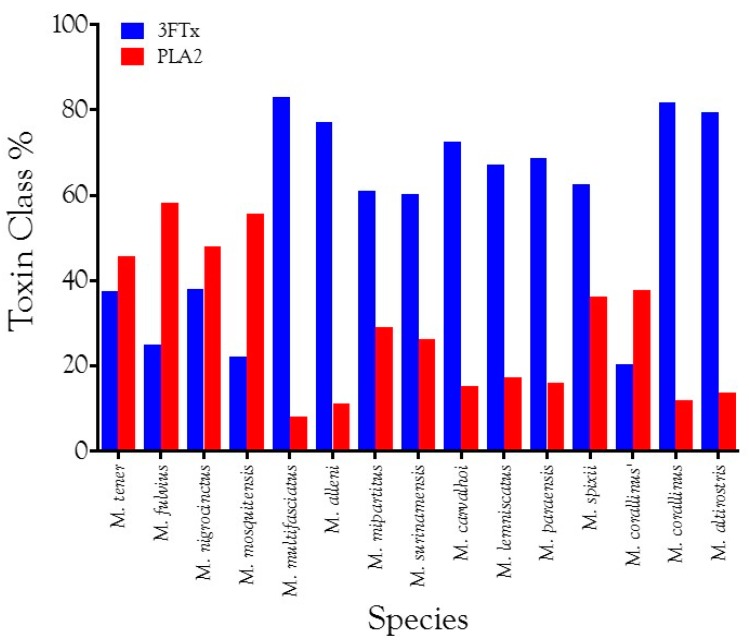
When *Micrurus* species for which transcriptomic data are available, are arranged in an approximately northwestern to southeastern sequence, the gradient of high PLA_2_ and low 3FTx concentrations in the North to high 3FTx and low PLA_2_ concentration in the South previously suggested by Fernández et al. [170], no longer appears as obvious.

Fernández et al. [170] compared *Micrurus* species for which venom gland transcriptomes are available and reported generalized north-south clines in PLA_2_ and 3FTx content. They opined that North American species are generally richer in PLA_2_ and poorer in 3FTxs, while South American species show a reverse trend. However, with the addition of the present data, the suggested clines break down, at least in part because the geographic rule does not consider the influences of either phylogeny or dietary ecology (Figure 11). The venoms examined in this study had PLA_2_ levels ranging from 15.3–37.7% (Table S2), with three of them below 17.2%. Populations of *M. corallinus* from southeastern Brazil do not necessarily follow the geographic trend trend suggested by Fernández et al. [170] (Figure 11).

For the sake of simplicity, the discussion below approaches micrurine phospholipases A_2_ on the basis of biological activity, but the result is more simplistic than simple, because nature disdains such classifications. Many micrurine PLA_2_s possess more than one biological activity (myotoxicity, cytotoxicity, neurotoxicity, anticoagulation, pro-inflammation, etc.), as has been shown for crotalid neurotoxic PLA_2_s [171].

**Figure 12:**
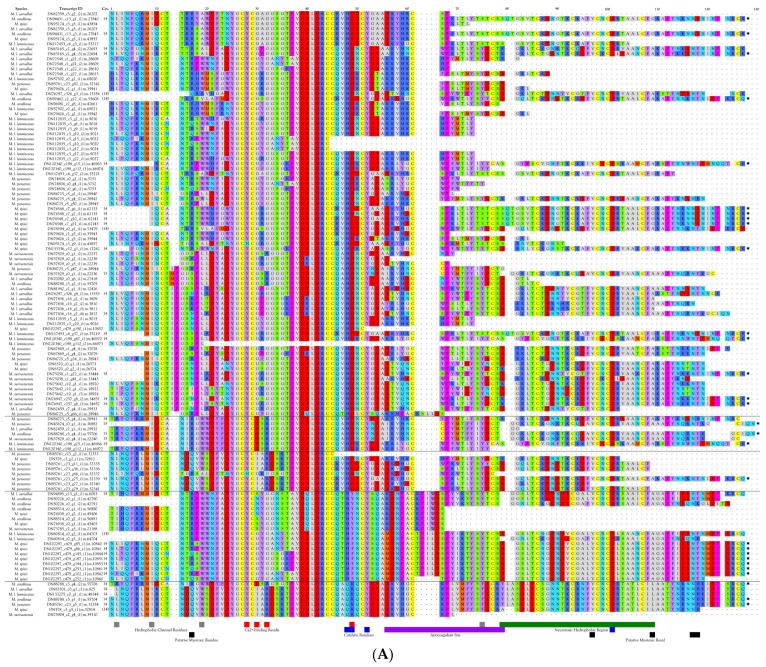

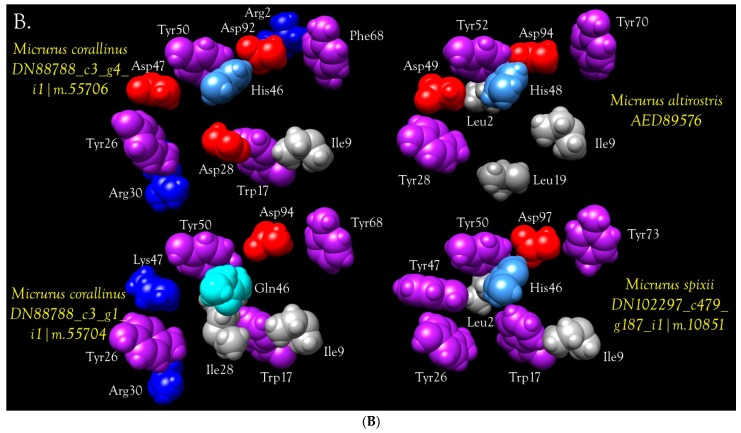
Structures of 121 new micrurine phospholipases A_2_, illustrating their great structural diversity. For a comparison of all known micrurine PLA2s, see Figure S22. (**A**) Of 121 PLA_2_s with partial or complete structures, 202 are apparently catalytic, having the requisite H48, D49, Y52, and D101 in their active sites. The remaining 42 are apparently non-catalytic. Positions indicated in red across the bottom designate residues involved in Ca^2+^-binding [172]. Positions indicated in blue are catalytic residues. The anticoagulant site [173] and the neurotoxic hydrophobic region [172,174] are indicated by purple and green bars, respectively. Residues proposed by Alape-Girón et al. [175] to be involved in myotoxicity of elapid PLA_2_s are indicated below with black cells. Residues participating in the hydrophobic channel, via which substrate enters the catalytic site are indicated in gray [176]. PLA_2_s from the two North American species, *M. fulvius* and *M. tener*, form a structural cluster that is relatively distinctive from the much more variable South American sequences, which probably also represent much greater pharmacological diversity. Numbers of cysteine residues are given for sequences that are complete. Numbers of cysteines in parentheses are for sequences truncated at the N-terminal end, for which the cysteines can be reliably predicted, owing to the relative invariability of the N-termini. Asterisks indicate stop codons.Micrurine venoms include 42 presumably non-catalytic PLA_2_s that appear to be myotoxic. These comprise four loose subclasses, shown here separated by horizontal lines. The first two groups possess all four catalytic residues, but apparently have a disrupted Ca^2+^-binding site (D or I in lieu of G30, and R in place of G32). Toxins in the third group have H48, but D49 has been replaced by Y49 or F49, and G30 with N or S. The fourth subclass, comprising 9 PLA_2_s from all six Brazilian species and *M. tener*, has replaced H48 with Q and D49 with K. G30 has been replaced with D, I, or V. Thus, it seems improbable that any of these can bind Ca^2+^; (**B**) Space-filling models of catalytic residues, Ca^2+^-binding residues, and residues forming the hydrophobic channels of four micrurine PLA_2_s. See Part A of this figure for the roles and locations of individual residues. The PLA_2_ from *M. altirostris* (upper right) is a catalytic toxin, possessing all of the essential residues. The other three are presumably non-catalytic. In the *M. s. spixii* toxin (lower right), the catalytic D47 has been replaced with F47. While the usual G28 and G30 are present in the hydrophobic channel (not visible in this projection), a bulky W17 has replaced L17, presumably disrupting the channel. In the *M. corallinus* toxin in the lower left, the catalytic H46 has been replaced by Q46. K47 has been substituted for D47, replacing a negative charge involved in Ca^2+^-binding with a positive charge. R30 has replaced G30 in the Ca^2+^-binding loop and W17 has replaced L17 in the hydrophobic channel. Lastly, in the *M. corallinus* PLA_2_ in the upper left, R2 and W17 occlude the hydrophobic channel, while R30 is present in what should have been the Ca^2+^-binding loop. SWISS-MODEL employed a cardiotoxic PLA_2_ with a D-loop from *Ophiophagus hannah* (1gp7.1.A) as a template for *M. spixii* DN102297. *M. altirostris* AED89576 was modelled after the subunits of a homotetrameric PLA_2_ from *Micropechis ikaheka* (1pow.1.ABCD). The model of *M. corallinus* DN88788-55704 was based upon the 3D structure of *M. tener* MitTxβ (4ntw.1.C), while that for *M. corallinus* DN88788-55706 was modeled after a PLA_2_ isoform from *Naja naja sagittifera* (1xxw.1.A). The latter is one subunit of a noncovalent heterodimer.

Micrurine PLA_2_s have transcripts that encode 115–120 residues plus 27-residue signal peptides. These PLA_2_ signal peptides terminate in paired hydroxylated amino acids, usually -SS-, but occasionally -ST- (Figure 12A and Figure S22). All of them possess a Cys residue in position -15, but they exhibit several N-terminal sequence patterns. Most of the signal peptides commence with MNPAH- or MILAH-, although a small number have MLIFLW-. The latter usually have one or two extra cysteines in position -19 or positions -18 and -19. There appears to be little relationship between signal peptide structure and expressed protein structure.

The present study yielded 121 new micrurine PLA_2_ sequences, partial and complete (Figure 12A). More significantly, all six *Micrurus* transcriptomes in this study possessed non-catalytic, presumably monomeric PLA_2_s, the first reported for elapid venoms. These toxins fall into four structural subgroups, depending upon which of the catalytic and Ca^2+^-binding residues have been substituted non-synonymously (Figure 12A,B). There were 42 such sequences, including two from *M. tener* venom.

Most micrurine PLA_2_s have the expected 14 cysteine residues (Figure 12A); however, because of mutations some have only 13. However, more than a dozen PLA_2_s have 15 Cys residues. Seven of these have an extra Cys at position 67, a variant structure not reported previously (Figure 12A). In the case of the nigroxins, the extra Cys occurs at position 59 [175]. PLA_2_ #27 from *M. fulvius* has a 15th Cys residue at position 102 (Figure 12A), but since no other PLA_2_ displays this variant, this is assumed just to be an unusual mutation. Whether this extra cysteine has any functional significance is unknown. Three toxins have an extra Cys at position 121. One of these, *M. surinamensis* DN37829_c0_g1_i1|m.22236, has extra Cys residues at both positions 67 and 121. Since it also has an incomplete C-terminus, we cannot rule out the possibility of a 16th cysteine at position 126. Nonetheless, it is probable that most of the 15-Cys toxins form an intermolecular disulfide bond with either another phospholipase, or with a non-homologous protein. A covalent homodimer seems most likely since the micrurine 15-Cys toxins do not align with the PLA_2_ subunit of β-bungarotoxin, which has only 13 cysteines. Nonetheless, non-PLA_2_ toxins with odd numbers of Cys residues, and which therefore could potentially bind covalently to 15-Cys PLA_2_s, include the long KSPIs (13 Cys)(Figure S16), serine proteases (most have 15)(Figure S25), serpins (33 Cys)(Figure S26), NGFs (7 Cys, some have 8)(Figure S20), MPs (39 Cys)(Figure S18), and CTLs (6, 7, 8, 9 Cys)(Figure S7). Of these, the long KSPIs seem unlikely, both because of their low abundance and their distributions. SPs, serpins, MPs, and NGFs seem unlikely by reason of their large sizes. The most likely candidates would be CTLs with either 7 or 9 cysteines, although such toxins would be a compositional novelty; however, such a toxin class might target and activate blood platelets very effectively.

In the vast majority of PLA_2_s, catalytic or non-catalytic, C11 is followed by T12 and then 7–8 other hydrophilic and/or basic residues (Figure 12A and Figure S22). However, 34 PLA_2_s follow T12 with an insert of IPG or MPG. The function of this structural novelty is unknown. Whether it serves to render these PLA_2_s resistant to degradation by prey proteases, after the manner of crotaline bradykinin-potentiating peptides, or whether it presents key residues to be recognized by molecular targets in prey tissues is unknown. PLA_2_s of this type are present in all six transcriptomes that we examined, and in the transcriptomes of *M. fulvius*, *M. tener*, and *M. altirostris* (Figure S22; Table S2). Those with the MPG sequence are extremely minor, ranging from 0.005–0.02% of the PLA_2_ content in their respective transcriptomes. The IPG forms are more abundant, representing from 0.6% of all PLA_2_s in the *M. paraensis* transcriptome to 86.2% in *M. surinamensis*. Our *M. corallinus* transcriptome did not have an IPG PLA_2_ (Figure 12A).

##### Myotoxic PLA2s

Gutiérrez et al. [177] found that six of seven *Micrurus* venoms they investigated (*M. alleni, M. frontalis, M. n. mosquitensis, M. n. nigrocinctus, M. dumerilii carinicauda, M. surinamensis*) induced rapid and qualitatively similar myonecrosis. Myonecrosis provoked by *M. nigrocinctus* venom was especially profound, while *M. surinamensis* exhibited much less activity. Only *M. mipartitus* (probably *M. multifasciatus*) venom was non-myotoxic. Gutiérrez et al. [178] later demonstrated that *M. nigrocinctus* venom released massive amounts of myoglobin, creatine, and creatine kinase, but caused relatively little hemolysis, unless calcium were present in the media. Whether phospholipolytic or cytotoxic in nature, the observed hypercontraction of myofilaments suggested that at least some micrurine PLA_2_s are myotoxic and that they destroy muscle largely via massive calcium influx [179], which also poisons mitochondria. The former authors also suggested that cardiotoxin-like molecules are probably also present in *M. nigrocinctus* venom, a conclusion also reached by Vital Brazil for *M. fulvius* venom [69]; however, this has yet to be confirmed in any *Micrurus* venom to date.

Arroyo et al. [180] purified a PLA_2_ from *M. nigrocinctus* venom and reported that after intramuscular (*i.m.*) injection, serum creatine kinase levels spiked within 1.5 h. They concluded that the toxin’s primary target is the plasma membrane. Thereafter, myofilaments were clumped, sarcoplasmic reticulum was disrupted, and some mitochondria were damaged. Goularte et al. [181] proposed that the *M. nigrocinctus* myotoxin initially binds presynaptically to the neuromuscular junction of mouse phrenic nerve-diaphragm preparations, followed by sub- and postsynaptic effects. They concluded that the latter were the most important cause of the neuromuscular blockade. A catalytic phospholipase A_2_ from *M. s. spixii* venom appears to present a similar picture, although there is insufficient information to draw any firm conclusions [182]. Carvalho et al. [183] demonstrated that in cultured rat primary hippocampal cells, two PLA_2_s isolated from *Micrurus l. lemniscatus* venom induced cell death, exhibiting elements of apoptosis, autophagy, and necrosis.

Lind and Eaker [184] noted that the Lys-Lys-Lys sequence (positions 100–102) in notexin is also encountered in porcine pancreatic PLA_2_ and in the myotoxic notechis II-5. They suggested that these residues might be essential for neurotoxicity, myotoxicity, or both. Alape-Girón et al. [175] systematically compared the sequences of the myotoxic nigroxins A and B from *M. nigrocinctus* venom with elapid PLA_2_s known to cause myonecrosis upon *i.m.* injection. These included *Naja mossambica* CM-I, CM-II, and CM-III [185], *Oxyuranus scutellatus* OS1, which binds in calcium-independent fashion to the muscle (M-type) PLA_2_ receptor [186], *Notechis scutatus* notexin and II-5, [187,188], and compared them with the β-bungarotoxin A chain and two pancreatic PLA_2_s known not to be myotoxic. They identified a series of amino acid residues that were highly conserved in the myotoxic PLA_2_s (Arg15, Val97, Ala109, Asn117, and an aromatic residue in position 118, Figure 12A) (residue numbers vary with the sequences compared and the resulting insertion of sequence gaps).

Gutiérrez and Ownby [189] noted that some myotoxic PLA_2_s exhibit myotoxicity even if injected intravenously, whereas others require *i.m.* injection to exert this effect. They proposed that the systemic myotoxins are sufficiently specific for muscle that they will find their way to cell membranes of skeletal muscle cells regardless of the route of injection. Lemnitoxin appears to illustrate this principle [190]. Other PLA_2_s, that are less specific for muscle than for some other target, e.g., neurotoxic PLA_2_s, bind to the more specific targets first, inducing myotoxicity secondarily, or if injected directly into muscle [189]. However, it should be emphasized that given the myriad structural variations of PLA_2_s, all of our attempts at classification are overly simplistic and are bound to prove unsatisfactory. Gutiérrez et al. [191] have discussed the complexities of such an undertaking in regard to myotoxicity, and Sampaio et al. [192] have more recently shown that secondary pharmacological effects continue to be discovered even for toxins as well studied as crotoxin. Moreover, in some cases, different PLA_2_s achieve the same pharmacological effect by different mechanisms [173].

Nuñez et al. [193] showed that a C-terminal 13-amino acid peptide from the K49 myotoxin of *Agkistrodon p. piscivorus* was able to lyse cultured skeletal muscle cells, indicating that it contained the structural elements necessary for myotoxicity; however, crotalid PLA_2_s have longer C-termini than micrurine PLA_2_s [159], so clearly, crotalids and micrurines accomplish myotoxicity by different means [190]. Of the 42 non-catalytic PLA_2_s, 32 retain H48, but 18 have replaced D49 with Y or F (Figure 12A). The remaining 9 PLA_2_s have replaced residues 48–50 with QKQ and are found in venoms of all six of the Brazilian species and that of *M. tener* (Figure 12A). Surprisingly, no non-catalytic sequence has been reported from *M. fulvius* to date, so far as we are aware. Two other groups of apparently non-catalytic PLA_2_s have the four catalytic residues, but apparently have a disrupted Ca^2+^-binding site (residues 30–32) (Figure 12A). At present, it does not appear possible to draw many conclusions regarding structure and function of micrurine PLA_2_s, owing to the lack of pharmacological studies; however, most micrurine PLA_2_s display all of the residues identified by Alape-Girón et al. [175] as being essential for myotoxicity (Figure 12A).

##### Hemorrhagic PLA2s

**Figure 13:**
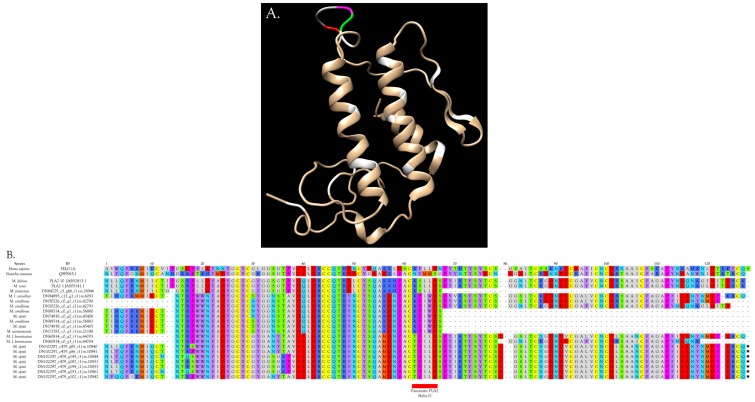
This study identified 18 putative micrurine hemorrhagic phospholipases (HPLA_2_s) in all six Brazilian *Micrurus* species examined. All have an extra D loop (colored residues) that sits atop the two main helices, like human pancreatic PLA_2_ and *Notechis scutatus* HT. These D loops all have one of three sequences: KSLLD, KPIWD, or TPILD. (**A**) A TPILD toxin from *M. s. spixii* (DN102297_c479_g187_i1|m.10851) modeled after a cardiotoxic PLA_2_ with a D-loop, from *Ophiophagus hannah* (1gp7.1.A); (**B**) All of the HPLA_2_s are non-catalytic, except for the KSLLD forms from *M. l. carvalhoi*, *M. fulvius*, and *M. tener*, which do have the requisite catalytic and Ca^2+^-binding residues in the active site, and key residues that form the hydrophobic channel.

Francis et al. discovered a novel, toxic Type I phospholipase in the venom of the Australian tiger snake (*Notechis scutatus*) [194,195]. These toxins cause transient hypotension, but acute organ damage due to hemorrhage; hence, they were denominated “hemorrhagic toxins.” Structurally what makes them unusual is that they retain Helix D, a loop of amino acids characteristic of pancreatic PLA_2_s, that sits atop the two large parallel α-helices (Figure 13A). This extra loop causes them to migrate on SDS-PAGE with apparent molecular weights of 18–23 kDa. From *M. frontalis* venom, Francis et al. [196] isolated basic, 21–23 kDa PLA_2_s that cross-reacted strongly with rabbit polyclonal antibodies raised against the hemorrhagic PLA_2_ from *Notechis s. scutatus* venom, and concluded that most or all of the PLA_2_s in *M. frontalis* venom are of this type. However, to date, no sequence has been published for any of the *M. frontalis* hemorrhagic PLA_2_s. Based on the presence of a Helix D-like structure, a probable hemorrhagic phospholipase sequence from venom of *M. fulvius* was published in 2013, although the authors did not identify it as such [1]. The present study identified 18 new micrurine hemorrhagic phospholipases in all six Brazilian species. These (and two sequences from *M. fulvius* and *M. tener*) show great sequence similarity to *Notechis* HT_e_ and to human pancreatic PLA_2_ (Figure 13B). Francis et al. [196] reported that the hemorrhagic phospholipases from *M. frontalis* venom lacked phospholipase activity, and now we can explain why. Of the 20 hemorrhagic PLA_2_s reported here, 17 have Tyr50 in the active site instead of Asp50; thus, they cannot bind the Ca^2+^ ion required for catalysis. The sequences from *M. fulvius*, *M. tener*, and *M. l. carvalhoi* do have Asp50 and probably are catalytic (Figure 13B).

The structural requirements for Helix D are not entirely clear, beyond the fact that it comprises five residues. *Micrurus* hemorrhagic phospholipases (HP) display one of three sequences: KSLLD, KPIWD, or TPILD. The third and fourth positions of HPs appear to always require a hydrophobic amino acid, either aliphatic, aromatic, or methionine, although the latter has only been found in the hemorrhagic toxin (HT) from *Notechis* [195], to date (Figure 13). Like the human pancreatic PLA_2_, all *Micrurus* HPs have aspartate in the fifth position, although *Notechis* HT has serine. The Brazilian *Micrurus* HPs all have proline in the second position, except for *M. l. carvalhoi*, which along with *M. fulvius* and *M. tener*, has serine, a strongly non-synonymous substitution. In the first position, most *Micrurus* HPs have lysine, as in pancreatic PLA_2_; however, a nearly equal number of toxins have threonine, and HT has asparagine. Thus, it appears that the first position simply requires a hydrophilic residue (Figure 13).

Intravascular hemolysis, manifested by hemoglobinuria and resulting in anemia, has been reported in dogs bitten by *M. fulvius* [197,198,199]. Arce-Bejarano et al. [200] confirmed that catalytically active PLA_2_s are responsible for the hemolysis and identified one of the three active fractions as being identical to PLA_2_ 2b reported by Margres et al. [1]. In vitro, dog and mouse erythrocytes were found to be highly susceptible to the PLA_2_s, while those of rabbits and humans were unaffected. The difference appeared related to a high ratio of phosphatidylcholine/sphingomyelin in erythrocyte plasma membranes. Mechanical stress, after incubation with venom, significantly increased hemolysis, suggesting that in vivo shear stresses associated with circulation probably contribute to cell lysis [200].

##### Neurotoxic PLA_2_s

Lambeau et al. [201] suggested that the diversity of pathophysiological effects evoked by snake venom PLA_2_s probably reflects the occurrence of specific receptors for them. They found that neuronal PLA_2_ receptors in brain display high affinity for toxic snake venom PLA_2_s, but not for non-toxic PLA_2_s. A second type of receptor, the muscle type, binds the hemorrhagic snake venom PLA_2_s with high affinity, as well as pancreatic and human inflammatory PLA_2_s, but not the neurotoxic snake venom PLA_2_s. It is likely that relatively small structural differences may account for these differing affinities.

A possible illustration of this principle is seen in MiDCA1, a presynaptic, monomeric phospholipase A_2_ from *M. dumerillii carinicauda* venom that produces a neuromuscular blockade in vertebrate nerve-muscle preparations [202]. MiDCA1 causes a spontaneous release of transmitter at neuromuscular junctions, followed by a depression of transmitter release, a pattern also observed in *M. laticollaris* venom [203]. The initial increase results from a direct effect of MiDCA1 on presynaptic potassium channels, and neurotoxicity is Ca^2+^-dependent, indicating that catalytic activity is essential for neurotoxicity [202].

MiDCA1 possesses an intact Ca^2+^-binding loop and an intact active site (Figure 12 and Figure S22). However, the authors noted that while MiDCA1 possesses a number of highly conserved amino acid residues that are generally considered necessary for myotoxicity (R17, V97, A109, N117, and Y118; numbering after Figure 12 and Figure S22) [175], it shows little myotoxicity. They surmised that its lack of myotoxicity might be due to its relatively low enzymatic activity [202]. However, MiDCA1 has E17 in place of the essential R17. In addition, MiDCA1 has V20 instead of W20 in the hydrophobic channel that is essential to admit the substrate, although L2, I9, and Y75 are present (Figure 12 and Figure S22). It is possible that active site inaccessibility could also contribute to low catalytic activity.

Neurotoxic PLA_2_s are significant venom components in the venoms of some species. Alapé-Giron et al. [204,205] reported that not only are PLA_2_s significant components of *M. nigrocinctus* venom, but that a number of these are antigenically related to notexin, the monomeric, neurotoxic PLA_2_ from *Notechis scutatus* venom. Concurrently, Goularte et al. [181,206] reported significant neurotoxicity in *M. nigrocinctus* venom. Oliveira et al. [207] reported on the neurotoxic effects (behavioral, EEG and histopathologic) of intrahippocampal injection of four neurotoxic PLA_2_s from *M. l. lemniscatus* venom. These toxins were convulsive, but not epileptogenic at low doses (2.1 µg/rat). Higher doses (1–2.9 µg/rat) caused massive neurodegeneration of the dorsal hippocampus within 7 days. Vergara et al. [153] reported that PLA_2_-containing fractions (14 different PLA_2_ variants) accounted for 58.3% of *M. fulvius* venom protein. Other species, such as *M. pyrrhocryptus*, manifest low PLA_2_ activity, with no myotoxicity or presynaptic neurotoxicity [208]. Bénard-Valle et al. [209] reported a PLA_2_ with very high catalytic activity, but no discernible toxicity. They suggested that its primary role might be digestion of prey. Unfortunately, these studies presented no sequences that can be linked to the pharmacology they reported.

Kini and Iwanaga [172] studied presynaptic neurotoxic venom PLA_2_s, which display a bewildering structural diversity, from monomers to heteropentamers. They argued that neurotoxic PLA_2_s have to be able to interact with membranes, and that a hydrophobic site must be essential. They proposed a region of about 30 amino acids from residues 79–109 (Figure 12 and Figure S22) as the likely binding site. Guanidination or carbamylation of basic residues in *Naja nigricollis* PLA_2_ had earlier been shown to reduce toxicity by nearly 90% without affecting catalytic activity [210,211,212]. This suggested that basic residues are probably involved in targeting the PLA_2_ to the presynaptic terminus.

To explain variable susceptibilities of different tissues to different PLA_2_s, Kini and Evans [213] proposed the existence of specific target sites on plasma membranes of target cell types. They suggested that these are recognized by specific pharmacological sites on PLA_2_s that are complementary to one another in terms of charges, hydrophobicity and van der Waal’s forces. The higher the affinity between the enzyme’s pharmacological site and its target, the more specific is the pharmacological activity of the PLA_2_. Target sites are usually proteins, and various target proteins have now been identified for different presynaptic PLA_2_ toxins. Binding constants for PLA_2_-target protein interactions are 4–6 orders of magnitude stronger than those for PLA_2_-lipid interactions [214].

Comparisons with notexin show that levels of acidic, basic, and hydrophobic residues in this region are generally similar among micrurine PLA_2_s, although many of the latter have one or two additional basic residues at the C-terminus, and their exact positioning differs somewhat from those of notexin (Figure 12 and Figure S22). This may reflect the preference of most *Micrurus* for reptilian prey, but these toxins may also be active against other vertebrate motor nerve terminals as well.

##### Anticoagulant PLA_2_s

Salazar et al. [215] reported that both *M. isozonus* and *M. tener* venoms inhibit ADP-induced platelet aggregation and concluded that inhibition could be due to the presence of disintegrins, ADPases, or to proteolytic digestion of ADP receptors. However, a more likely explanation is that inhibition was caused by Type I PLA_2_s, which are well known from elapid venoms [216,217,218,219,220,221], and that are able to inhibit aggregation induced by ADP, collagen, and epinephrine. Depending upon the species and the PLA_2_ in question, this inhibition may involve catalytic or non-catalytic mechanisms.

**Figure 14:**
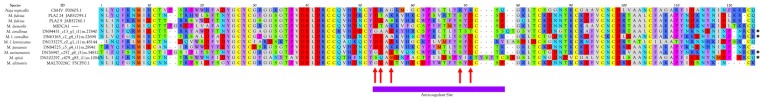
Weakly anticoagulant PLA_2_s differ from strongly anticoagulant enzymes, such as CM-IV, at five amino acid residues, when tested against mammalian blood. *Micrurus* PLA_2_s lack the residues in CM-IV that mimic coagulation Factor Va light chain and tissue factor [176]. The vast majority of micrurine PLA_2_s have 4–6 basic residues in the anticoagulant site, whereas *Naja nigricollis* CM-IV has only three. In positions 55–60, where CM-IV has only a solitary Lys, most *Micrurus* PLA_2_s have four His, Lys, or Arg residues (positions 57–61 in Figure 12 and Figure S22). It is impossible to predict what effect this might have on specific binding to vertebrate coagulation factors, but this may be a modification for reptiles or fish. Many *Micrurus* PLA_2_s are probably at least weakly anticoagulant.

While antiplatelet activity is anticoagulant in a broad sense, antiplatelet activities should not be confused with PLA_2_-mediated “anticoagulant” activity, which refers to inhibition of the external tenase and/or prothrombinase complexes [173]. According to Kini, the anticoagulant structural region embraces residues 56–79 in Type I PLA_2_s (Figure 12, Figure 14 and Figure S22). Strongly anticoagulant enzymes possess more basic residues in this region, while weakly anticoagulant enzymes possess more neutral or acidic residues. *Naja nigricollis* CM-IV, a strong anticoagulant, binds coagulation factor FXa and prevents formation of the prothrombinase complex [173]. While there have not yet been comparable studies of anticoagulant pharmacologies of micrurine PLA_2_s, it seems likely that like CM-IV, some of the catalytic PLA_2_s will also inhibit the prothrombinase complex by a mixture of enzymatic and non-enzymatic mechanisms.

Kini and Evans [222] concluded that weakly anticoagulant PLA_2_s differ from strongly anticoagulant enzymes, such as CM-IV, at five amino acid residues. Micrurine position numbers, in parentheses, correspond to those in Figure 14.

1. negatively charged Glu-54 (53) is replaced by neutral residues;
2. positively charged Lys-55 (54) is replaced by negatively charged Glu;
3. uncharged Gly-57 (56) is replaced by negatively charged Glu;
4. positively charged Lys-75 (69) is replaced by Ser or Thr;
5. positively charged Lys-77 (71) is replaced by negatively charged Glu or Asp

The *Micrurus* PLA_2_s do not offer simple comparisons.

1. Half of micrurine PLA_2_s (49.7%) have Asp in position 54 (position 53 in Figure 14), like CM-IV. Gly or Ala occupy this position in 37.8%, while 9.8% have a hydroxylated amino acid, and 2.6% have Asn. See Figure 12, Figure 14, and Figure S22 for all PLA_2_s;
2. No micrurine PLA_2_s have Lys in position 53 (position 54 in Figure 14). All have Glu (41.8%), Gln (8.9%), Asp (6.8%), or Ala (13.1%), Thr (29.5%), like the weak anticoagulants;
3. Most *Micrurus* PLA_2_s (53.2%) have Glu in position 57 instead of Gly (Figure 14). 30% have Lys; 4.2% have Ser, and 0.4% have Tyr. An additional 11% have an aliphatic/hydrophobic residue (Ile, Ala, Met, or Val);
4. No micrurine PLA_2_s have Lys in position 75 (position 69 in Figure 14). Overwhelmingly they have Ser (80.4%). An additional 14.2% have Thr and 5.4% have Ile;
5. Only 5% of micrurine PLA_2_s have Lys in position 77 (71 in Figure 14), whereas 50% have Asp. Other substitutions include Thr (27.7%), Tyr (4.0%), Glu (10.4%), and Asn (3.0%).

*Micrurus* PLA_2_s appear to lack residues in CM-IV that mimic coagulation Factor Va light chain and tissue factor [173]. If this were not sufficiently complicated, the vast majority of micrurine PLA_2_s have 4–6 basic residues in the anticoagulant site, whereas *Naja nigricollis* CM-IV has only three (Figure 12 and Figure 14). In fact, in positions 54–60, where CM-IV has only a solitary Lys, most *Micrurus* PLA_2_s have four His, Lys, or Arg residues (Figure 14; positions 57–61 in Figure 12A). It is impossible to predict what effect this might have on specific binding to vertebrate coagulation factors, but perhaps this is a modification for reptilian prey. Many *Micrurus* PLA_2_s may be weakly anticoagulant against mammalian platelets, but they should be tested against fish or reptile platelets before any conclusions are drawn.

##### Pro-Inflammatory PLA2s

Some micrurine PLA_2_s also exhibit pro-inflammatory activity. Intraplantar injection of lemnitoxin (*M. l. lemniscatus*) in rats induced dose- and time-dependent edema of rapid onset [195]. It was detectable after only 5 min and remained undiminished for 6 h, disappearing after 24 h. Doses of 100–500 µg of lemnitoxin provoked substantial degranulation of mast cells, and the release of histamine and serotonin resulted increased vascular permeability [190].

#### 2.2.23. Phospholipase B (PLB)

First discovered in the venom of the Australian elapid, *Pseudechis porphyriacus*, by Doery and Pearson [223], phospholipase B (PLB) apparently contributes to generalized hydrolysis of cell membrane lipids. A single PLB gene is present in venom glands of all six species examined and in those of *M. fulvius* as well (Figure S23). They range from 547–551 amino acids in length and mRNA transcripts lack a signal peptide. Six invariant Cys residues suggest three disulfide bonds; however, in a Trp-rich segment (positions 18–24) the *M. l. lemniscatus* and *M. s. spixii* toxins have an extra Cys, while the *M. l. carvalhoi* and *M. surinamensis* toxins have two more (Figure S23). Beyond this segment, the PLBs show remarkably little structural variation. They are not major toxins, ranging in abundance from 0.01% in the venom of *M. l. carvalhoi* to 0.59% in that of *M. corallinus*.

#### 2.2.24. Ribonuclease (RNAse A)

RNAse is essentially absent in all of the transcriptomes, except for that of *M. surinamensis*, where it comprises 0.7% of the toxin transcriptome (Figure S24; Table S2). Micrurine RNAse transcripts have 141 residues and apparently lack signal peptides. The sequences are largely invariant, showing amino acid substitutions, all non-synonymous, at only 3 positions (V/D60; R/G63; F/S85) (Figure S24). Oddly, 3 of our 6 *M. l. carvalhoi* transcripts and both transcripts from *M. l. lemniscatus* had a stop codon at position 118 rather than the usual position 142. As with nearly everything else in *Micrurus* venoms, the functional significance of this truncation is unknown. The loss of the C-terminal 24 residues suggests that that these have become pseudogenes, since the next 17 residues after the stop codon are the same as in full-length RNAse transcripts (Figure S24). However, we cannot exclude the possibility that these apparently dysfunctional enzymes have adopted another function instead.

#### 2.2.25. Serine Proteases (SP)

A single serine protease transcript of 265 residues was found in each of the six *Micrurus* venoms examined in the present study (Figure S25). The signal peptide accounts for the first 18 residues. These align well with the transcript published from *M. tener* venom. Two additional serine protease transcripts from the transcriptome of *M. fulvius* do not appear to be closely related to the other seven. Given the great similarity of the micrurine serine proteases presented here, the *M. fulvius* sequences may represent contaminating blood or tissue serine proteases. When the *M. paraensis* sequence was used as a query string, the most similar sequence was a trypsin-like serine protease from *Hydrophis hardwickii* venom [224]. Harobin is anti-coagulant, and has both fibrinolytic and fibrinogenolytic activities. It exhibits a preference for the Bβ-chain of fibrinogen, but eventually cleaves the Aα-chain and the γ-chain after prolonged incubation. In vitro, harobin is hypotensive, cleaving high molecular weight kininogen so as to release bradykinin, and also angiotensin-2 [224]. While the presence of this serine protease is consistent with snake envenomation strategies elaborated by [46], it is a very minor component in all venoms examined (≤0.1%), and it probably contributes relatively little to envenomation sequelae.

#### 2.2.26. Serine Protease Inhibitors (Serpins)

Serpins constitute a protein superfamily, comprising 16 clades [225]. While named for their ability to inhibit serine proteases of the chymotrypsin family, some are also capable of inhibiting members of other protease families, while others lack inhibitory activity altogether [225]. Micrurine serpins consist of 501-504 residues plus a 19-residue signal peptide; thus, they are roughly twice the size of the proteases they inhibit. They are approximately 6x longer than KSPIs (Figure S26).

In *Micrurus* venom glands, these transcripts are almost invariant, showing amino acid substitutions at only about 24 positions. Interestingly, while protease inhibitors generally have 1:1 stoichiometry with their targets, serpins are able to irreversibly disrupt protease structure [226], detach, and move to another target molecule to repeat the inhibition [227]. If serpins truly are venom constituents, they are extremely minor, ranging from 0–0.04% of the transcriptomes examined in this study. In various *Micrurus* venoms, KSPIs are much more abundant than serpins, but if they can function in a pseudo-enzymatic manner, absolute concentration would be less significant.

To the best of our knowledge, no studies of snake venom serpins have been undertaken, but serpins are constituents of parasitoid wasp venoms and the saliva of ticks and mosquitos [228]. Their employment by hematophagous arthropods probably holds the key to their presence in snake venoms. Serpins isolated from saliva of *Aedes* mosquitos inhibit Factor Xa by an atypical mechanism involving reversible inhibition with a 1:1 stoichiometry [229,230]. Ticks rely primarily upon serpins to render host blood incoagulable. A serpin from the saliva of *Ixodes ricinus*, called Iris, acts as an anticoagulant, by interfering with platelet aggregation and by delaying fibrinolysis [231]. A second serpin, Irs-2, is capable of inhibiting thrombin and Cathepsin G [232]. Other tick salivary serpins have similar activity profiles [233,234,235]. We suggest, therefore, that the most probable role for snake venom serpins is anticoagulation.

#### 2.2.27. Vascular Endothelial Growth Factors (VEGF)

VEGFs are widely distributed in snake venoms, and comprise long (VEGF-C) and short VEGF (VEGF-F) subclasses. VEGF-Cs consistently have mRNA transcripts comprising 421 residues, with a signal peptide of 16 residues. Seven micrurine VEGF-C sequences have been determined to date, including six from this study. Interestingly, they are essentially structurally invariant, with the only deviation being a conservative I to F substitution at position 97 (Figure S27). No VEGF-C sequence has been reported from *Micrurus tener*, which does have a VEGF-F. VEGF-Cs, known primarily for their role in lymphangiogenesis [236], have not been reported previously as venom components, and the low expression levels seen here (0–0.02% of the venom gland transcriptomes) do not argue that they are; however, their capacity to induce increased vascular permeability [237] could predispose them to co-option for that purpose.

VEGF-Fs (211–235 residues) are structurally more complex, with at least three major structural variants, although at individual amino acid positions, like the VEGF-Cs, they are extremely conservative. Variable positions include either E or Q at position 5, G or D at position 12, S, P, or A at position 110, and T or I at position 113 (Figure S28). Following an invariant E116, strange things begin to happen. Position 117 may be N or K, and in transcripts from *M. corallinus*, *M. fulvius*, and *M. surinamensis*, it is missing altogether (Figure S28). Toxins with K at this position then have an extremely basic, 24-residue insert that is absent in the N117 or -117 toxins (Figure S28). These toxins have 14 K or R residues from positions 118–137. All sequences are invariant from residues 142–185. Position 185 is R in all known sequences, except for the *M. corallinus* transcript mentioned above, in which K is present. The majority of VEGF-Fs have a hydrophilic 25-residue C-terminal sequence, commencing with a short hydrophobic segment, YLHLL; however, three toxins terminate with a short, basic sequence, CEKPRR.

#### 2.2.28. Vespryns (VESP)

Pung et al. [238] isolated a novel 12-kDa toxin from the venom of the king cobra that acts centrally to induce hypolocomotion and pain in mammalian prey. Named ohanin, it became the first snake venom vespryn. Additional vespryns have subsequently been sequenced from various elapids [239] and crotalids [2,10,240,241], and from the viperid, *Echis coloratus* [242]. Crotalid vespryns are 28–32 residues longer at their N-termini than the elapid toxins.

All six Brazilian *Micrurus* venoms possess a single vespryn sequence, except for the venoms of *M. paraensis* and *M. l. carvalhoi*, in which there were two (Figure S29). Their sequences are nearly identical to that of ohanin. Micrurine vespryn mRNA transcripts comprise 190 residues and lack signal peptides. No vespryns have been reported from *M. fulvius* venom, although a search of the NCBI nr database produced two sequences in *M. fulvius* that share several 3–4-residue sequence segments with the *Ophiophagus* toxin (NCBI GAEP01000222.1 and GAEP01001746.1). Nonetheless, their sequence similarities to other micrurine vespryns are so limited that functional identity seems unlikely. Interestingly, the closely related *M. tener* does have a legitimate vespryn sequence. Vespryn transcripts have also been reported in the venom gland transcriptomes of *M. altirostris*, where they comprised 0.2% of the transcriptome [95]. Moreover, vespryn peptides have been sequenced from *M. nigrocinctus* venom, comprising 3.8% of the proteome [154]. Vespryns appear at only trace levels in most of the venoms examined here, although they comprised 0.22% of the *M. s. spixii* transcriptome and 1.66% in *M. corallinus*.

#### 2.2.29. Waprins (WAPR)

Waprins have been reported to have anti-bacterial [243] and protease inhibitory activity [244]. It is unclear whether either of these activities is germane to envenomation, although a waprin-like sequence fused to a KSPI domain was identified in the venom gland transcriptome of *Sistrurus catenatus edwardsi* [245]. This suggests that protease-inhibitory activity may be the key. Waprin-like sequences are present in all six *Micrurus* venoms examined in this study, but they have not been reported in venoms of *M. fulvius* [1], *M. tschudii* [246], *M. clarki* [247], *M. alleni*, or *M. mosquitensis* [170]. Micrurine waprin-like sequences are more similar to the 135-residue colubrid opisthoglyph and crotalid waprin-like sequences than to those of Old World elapids (78–80 residues) (Figure S30). Colubrid and crotalid waprin-like proteins have 23-residue signal peptides; however, none of our sequences have complete signal peptides and the sequence from *M. tener* is so different at the N-terminus that no definite conclusions can be drawn regarding the lengths of other micrurine toxins. However, it appears that all micrurine waprin-like sequences have an N-terminal Glu, as in the colubrid and crotalid sequences (Figure S30). Waprin-like toxins are minor components of micrurine venoms, ranging from essentially 0 to 0.44% in *M. l. lemniscatus* venom.

#### 2.2.30. Phylogenetic Conclusions

**Figure 15:**
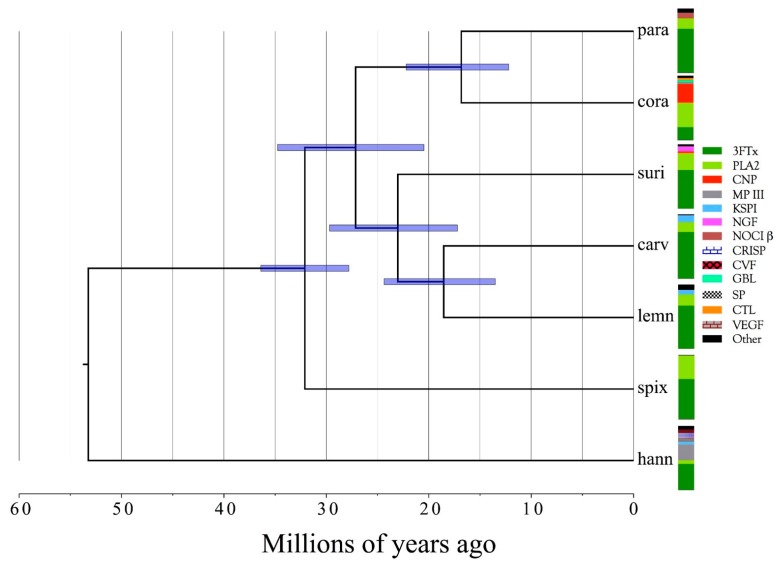
A phylogenetic tree based upon all venom and tissue proteins (4650 protein families) in the six transcriptomes suggests that these *Micrurus* species diverged 15–35 million years ago. The New World elapids split from the last common ancestor with Old World elapines nearly 55 million years ago. The tree indicates close relationships between two monad-banded species, *M. corallinus* and *M. paraensis*, and between *M. l. lemniscatus* and *M. l. carvalhoi*. *Micrurus surinamensis* is closely allied to *M. l. lemniscatus* and *M. l. carvalhoi*. *Micrurus s. spixii* is not particularly close to any of the other species, supporting the assertion of Slowinski [249] that it is more closely related to the triad-banded species of the *M. frontalis* complex in the Brazilian cerrado. The *Ophiophagus hannah* genome [250] provided data for the outgroup to root the tree. Blue bars indicate the 95% confidence intervals for the nodes. Taxonomic abbreviations: para (*M. paraensis*), cora (*M. corallinus*), suri (*M. surinamensis*), carv (*M. l. carvalhoi*), lemn (*M. l. lemniscatus*), spix (*M. s. spixii*), and hann (*O. hannah*).

We used all venom and tissue protein transcripts (4650 protein families) with the *Ophiophagus hannah* genome as an outgroup to estimate relationships among the *Micrurus* species examined here (Figure 15). The data suggest that these six taxa diverged from one another 15–35 million years ago, and from the last common ancestor with Old World elapines about 55 million years ago. Given this early divergence, we expect to find numerous cryptic species among the micrurines. It is apparent that *M. corallinus* and *M. paraensis* are closely related (Figure 15). This is unsurprising, given that both are monad-banded species. Nor is the close relationship between *M. l. lemniscatus* and *M. l. carvalhoi* unexpected, given their former subspecific status [248,249]. *Micrurus surinamensis* is more closely related to *M. l. lemniscatus* and *M. l. carvalhoi* than to the monad-banded species and *M. s. spixii*. Most interesting is the distant relationship of *M. s. spixii* to the other five species (Figure 15). Slowinski et al. [249] proposed that *M. s. spixii* is more closely related to the triad-banded species of the *M. frontalis* group, from the cerrado of central Brazil. While these data do not suggest whether it is closer to *M. corallinus* and *M. paraensis* or to *M. l. lemniscatus*, *M. l. carvalhoi*, and *M. surinamensis*, the latter seems far more likely based upon morphological data and it will be interesting to see whether high-throughput transcriptomes from other species will eventually confirm this supposition.

**Figure 16:**
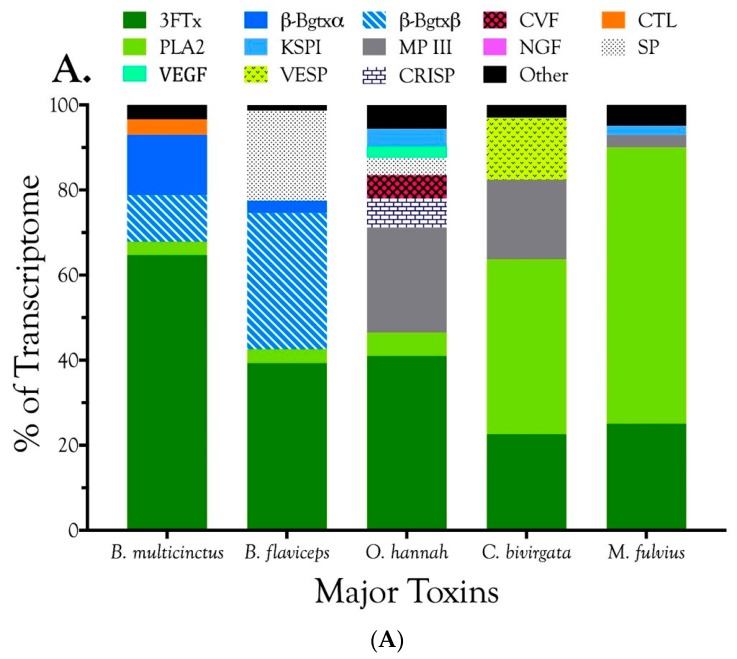

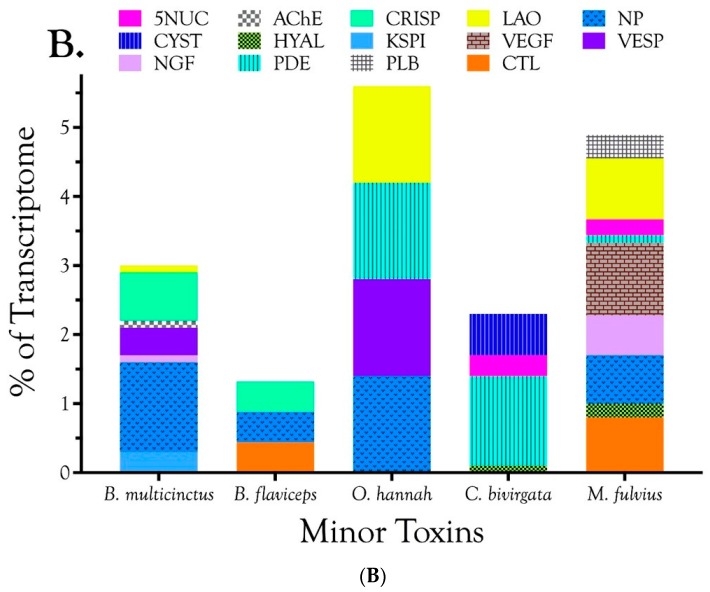
Transcriptomes of Old World elapids most closely related to *Micrurus* show highly divergent venomes in terms of both major (**A**) and minor (**B**) venom constituents. 3FTxs are major components of all of these venoms; however, phospholipase content varies significantly. Phospholipases A_2_ are a much more significant proportion of *Calliophis* and *Micrurus* venoms than of *Bungarus* or *Ophiophagus* venoms, even when β-bungarotoxin is considered. Venoms of *Bungarus multicinctus* and *Bungarus flaviceps* show significant quantities of β-bungarotoxin, although with strongly unequal quantities of the α and β subunits. *Ophiophagus hannah* and *Calliophisbivirgata* venoms possess large quantities of metalloproteases which are absent in kraits and minor components in most coralsnake venoms. Minor constituents are even more diverse.

Micrurine transcriptomes developed during the present study were compared with those of three Old World ophiophagus species, *Bungarus flaviceps*, *Bungarus multicinctus,* and *Calliophis bivirgata* and with the genome of *Ophiophagus hannah* [250,251,252,253]. The former two transcriptomes were developed without the use of next-generation sequencing technology. As a result, they lack resolution with regard to minor venom components. Nonetheless, comparisons were instructive (Figure 16). Contrary to our expectations, venoms of different snake-eating taxa shared few attributes (Figure 16A). In all of these venoms, 3FTxs were major components. PLA_2_s are major components in *M. fulvius* and *Calliophis*, but relatively minor components in the other three Old World species (Figure 16A). Both kraits possess β-bungarotoxin, a krait invention, but the other three lack it. Metalloproteases are significant constituents of *Calliophis* and *Ophiophagus* venoms, but minor in *M. fulvius* and missing in the kraits (Figure 16A). Cytotoxins/cardiotoxins, generally considered a cobra specialty, are absent in *Ophiophagus* venom. The minor toxin classes present an even more varied picture, if that be possible (Figure 16B).

## 3. Conclusions

The present study approximately triples the amount of New World coralsnake venom protein data in the public domain. Despite the fact that all of these taxa eat snakes and amphisbaenians (*M. surinamensis* and *M. l. lemniscatus* also take fish) the six venoms profiled here vary dramatically in composition, indicating that there is more than one way to efficiently kill the same type of prey organism. Coralsnakes experiment continually with venom composition, both qualitatively and quantitatively. Micrurines may well prove to have the most diversified venoms of any snakes, offering a treasure trove of ligands with novel and potentially useful pharmacologies and these six venomes differ dramatically. In each, 2–6 toxin classes account for 91–99% of toxin transcripts. Other toxin families are present in all six venoms at trace levels (<0.005%). In addition to 3FTxs and PLA_2_s, all venoms also contain nociceptive toxin (MitTx) subunit β, phospholipase B, and vascular endothelial growth factors. Minor components (0.1–2.0%) are found in all venoms except that of *M. s. spixii*. The most abundant venom components after 3FTxs and PLA_2_s differ from venom to venom. Numerous novel toxin chemistries include 3FTxs with previously unknown 8- and 10-cysteine arrangements, suggesting new 3D structures and target specificities. 9-cysteine toxins raise the possibility of covalent, homodimeric 3FTxs or heterodimeric toxins with unknown subunit compositions and pharmacologies.

## 4. Materials and Methods

### 4.1. Collection of Snakes and Venom Samples

All specimens except for that of *M. corallinus*, from Rio de Janeiro, Brazil, were captured in the municipality of Altamira, Pará, Brazil (Table S3). Prior to venom extraction, snakes were kept at low temperature (±4 °C) to reduce their physical activity. Individual venom samples were obtained by placing each fang into a microcapillary tube (70 µL). Glands were manually massaged and then the venom was transferred to individual Eppendorf tubes (2.0 mL) and stored in liquid nitrogen until lyophilization.

Given the tremendous geographic range of *M. surinamensis*, venom samples from a second specimen captured at Altamira and from two other specimens collected at Estreito, Maranhão, Brazil, were subjected to proteomic comparisons only in an effort to assess intraspecific variation in this species.

### 4.2. Collection of Tissue Samples

Four specimens of each species from the same locality were used for sample collection. Tissues were collected immediately after administration of a lethal dose of B-Euthanasia (sodium pentobarbital). Samples of visceral organs (heart, liver, kidneys, testes, and skeletal muscle) were extracted, transferred to individual cryotubes (2.0 mL), and immediately frozen in liquid nitrogen. Two aliquots of each tissue sample were also preserved in 100% ethanol until use for DNA amplification and sequencing. Venom glands were extracted under a stereoscopic microscope to clear all attached muscle tissue. Glands were then subdivided into three or four smaller pieces and transferred immediately to individual cryotubes with RNAlater (Qiagen, Venlo, The Netherlands). They were maintained at 4 °C until RNA extraction and analyses. Specimens were collected under permit from the Brazilian Institute of the Environment and Renewable Natural Resources (Instituto Brazileiro do Meio Ambiente e dos Recursos Naturais Renováveis) (IBAMA), 02001.001848/2006-75, under ACCTMB 473/2014 and 647/2015.

### 4.3. Transcriptomics

#### 4.3.1. Removal of Venom Glands

Specimens were anesthetized with sodium pentobarbital injected into the dorsolateral musculature. They were considered fully anesthetized when all three of the following conditions were met: lack of righting reflex, lack of withdrawal reflex when the tail was tapped repeatedly with a finger, lack of response when the venter was stroked with a finger. The supralabial scales and skin were separated from the underlying tissues with a scalpel. Then the venom duct was severed with a fine scissors just behind the maxilla. Using a scissors, the gland and underlying muscle were cut away, placed in a pre-labeled cryotube containing RNAlater^®^ (Sigma^®^, St. Louis, MO, USA), (1 mL for up to 100 mg tissue). Tissue samples were maintained in RNAlater up to three days at 37 °C, or up to one week at 25 °C, and then transferred to a −80 °C freezer until RNA extraction.

#### 4.3.2. Isolation of Total mRNA from Venom Glands and Muscle

RNA was isolated using Trizol reagent (Thermo Fisher Scientific, Waltham, MA, USA). Venom gland samples (50–100 mg) were suspended in 1 mL of Trizol reagent, incubated on ice for 10 min, and then homogenized with a TissueRuptor (Qiagen). Probes used for homogenization had been previously treated with DEPC (Diethylpyrocarbonate—Sigma^®^). For phase separation, 0.2 mL of chloroform were added per mL of Trizol. Samples were shaken vigorously by hand for 15 s and incubated for 15 min on ice. Samples were then centrifuged at 12,000× *g* for 15 min at 4 °C. Each sample was separated into a lower red phenol-chloroform phase, an interphase, and a colorless, upper aqueous phase. Aqueous phases were carefully removed by angling the tubes at 45° and pipetting the aqueous solutions into new tubes. RNA was isolated by adding 0.5 mL of 100% isopropanol to each collected aqueous solution. Samples were vortexed gently, incubated at room temperature for 10 min, and then centrifuged at 12,000× *g* for 10 min at 4 °C. Supernatants were discarded, leaving gel-like or white pellets that contained the RNA. Pellets were washed with 1 mL of 75% ethanol per sample and then centrifuged at 7500× *g* for 5 min at 4 °C. Washes were discarded and pellets were air dried for 5–10 min. Pellets were subsequently suspended in RNase-free water. RNA quantification was carried out with a Thermo Scientific NanoDrop™ 2000 Spectrophotometer (Thermo Fisher Scientific, Waltham, MA, USA), and the 260/280 nm absorbance ratio was used to assess RNA purity. A ratio of ~2.0 was generally accepted for RNA. RNA sample integrity was also evaluated using denaturant agarose gel electrophoresis. Qiagen RNeasy Micro Kits (Qiagen, Venlo, The Netherlands) were used to clean and concentrate RNA samples. Lyophilized samples were shipped from Brazil to OIST Graduate University for further processing.

#### 4.3.3. mRNA Isolation and First and Second Strand cDNA Synthesis

With very minor modifications mRNA purification and cDNA synthesis essentially followed the protocols in Aird et al. [11]. Detailed methods are elaborated in the Supplementary Information and RIN numbers are provided in (Supplementary Table S3).

#### 4.3.4. Library Sequencing

Size selection was performed on amplified libraries using 12.0/12.5% double solid phase reversible immobilization [254] and 300- to 700-bp fragments were selected. Libraries were pooled so that all contained the same amount of cDNA. Pooled libraries were then size-selected by gel extraction on a Minelute column (Qiagen) to achieve an optimal insert size for the Illumina Hiseq PE150 (~500 bp). Size distribution of the library was confirmed using an Agilent 2100 BioAnalyzer (Agilent Technologies, Waldbronn, Germany), and library concentration was determined using qPCR. Pooled and sized libraries were sent to Beijing Genomics Institute (Shenzhen, China) for sequencing.

### 4.4. Proteomics

Protein reduction and carboxyamidomethylation methods and protein cleavage techniques are found in the Supplementary Materials and Methods.

#### 4.4.1. Protein Sequencing by Liquid Chromatography-Mass Spectrometry

All samples were analyzed using a Thermo Scientific Q-Exactive Plus Orbitrap hybrid mass spectrometer (Thermo Fisher Scientific, Waltham, MA, USA). The mass spectrometer was equipped with an HPLC (Dionex Ultimate 3000 nanoRSLC), an autosampler (HTC PAL, CTC Analytics) and a nanoelectrospray ion source. Each sample was injected in a volume of 5 μL, separated on a Zorbax 300SB C_18_ column (0.3 × 150 mm; Agilent, Agilent Technologies, Waldbronn, Germany) at 40 °C. A one-hour gradient was employed (1%B to 32%B in 45 min, 32%B to 45%B in 15 min, with a final wash at 75%B for 5 min and reequilibration at 1%B for 10 min.). Solvent A was 2% acetonitrile in 0.1% formic acid, and solvent B was 98% acetonitrile and 0.1% formic acid. A flow rate of 3.0 μL/min was used for peptide separation. The temperature of the heated capillary was 300 °C, and 1.9 kV spray voltage was applied to all samples. Mass spectrometer settings were: full MS scan range 350 to 1500 *m*/*z*, with a mass resolution of 70,000,30 μs scan time, and AGC set to 1 × e^6^ ions, and fragmentation MS^2^ of the 20 most intense ions.

#### 4.4.2. Protein Identification

Thermo RAW data was directly analyzed on Mascot (version 2.4, Thermo Fisher Scientific, Waltham, MA, USA) and Proteome Discoverer (version 1.4, using Sequest HT, Thermo Fisher Scientific, Waltham, MA, USA). Search parameters for both algorithms were: a maximum of two missed cleavages, with precursor and fragment mass tolerance set to 20 ppm and 0.01 Da, respectively. Carboxyamidomethylation of cysteine was set as a fixed modification, while methionine oxidation, asparagine and glutamine deamidation, and N-terminal acetylation were set as variable modifications for tryptic and chymotryptic digests. Asparagine and glutamine deamidation and formylation of peptide N-termini were set as variable modifications for formic acid digests. Reagents used for sequencing (trypsin, R and K; chymotrypsin, F, L, W, and Y; formic acid, D and M) were specified in each case. For relative quantification, we used the peak areas of the three most abundant, unique peptides per protein. 

A database of our transcript data was constructed using TransDecoder [255], with default parameters and PFAM database lookup. These relaxed settings were chosen to avoid the loss of small, naturally occurring peptides in the venoms. The common Repository of Adventitious Proteins—cRAP [256] was merged with this constructed database for searching. 

#### 4.4.3. Sequence and Structural Analyses

Sequences were aligned and compared using Geneious v 8.1.8 [257]. Signal peptides were predicted using SignalP 4.0 [258]. Three-dimensional molecular models were created using SWISS-MODEL [259], which matches a novel amino acid sequence to homologous sequences for which solved crystal or NMR structures are available. Overhanging N- or C-terminal residues not modeled by SWISS-MODEL were added with UCSF Chimera, with which disulfide bonds were formed and energy minimizations were executed [21]. 

### 4.5. Bioinformatics

#### Transcriptome Assembly and Quantitation

Sequencing was performed by BGI (Shenzhen, China) on a HiSeq 4000 in PE 250 mode, with adaptor filtering as part of the service. Libraries had 48.3 ± 7.9 S.D. million read pairs comprising 14.5 ± 2.4 S.D. Reference transcriptomes were assembled using Trinity (2.1.1) [260] with default parameters after trimming with Trimmomatic, with the SLIDINGWINDOW:4:30 MINLEN:65 parameters and Nextera adaptor sequences [261]. Reads were then re-mapped to the assembly using the RSEM (v1.2.13) pipeline, with bowtie2 as the mapper [262,263], to determine Fragments per kilobase mapped (FPKM).

### 4.6. Phylogenetics

In order to generate a phylogenetic tree, predicted proteins were first grouped using OrthoMCL v2.0.9 [264] with default parameters percentMatchCutoff = 50; evalueExponentCutoff = −5 in the config file. Clusters with fewer than 4 sequences were discarded, yielding 4560 families. For each cluster, only one sequence was retained from each species, and duplicates were removed. Translated proteins (using bioPython v.1.64 [265] were aligned with Muscle v3.8.31 [266]. Finally, a phylogenetic tree was constructed using RAxML v.7.3.5 [267], running 100 bootstrap replicates: raxml -T 24 -m PROTGAMMAWAG -s {input.phy} -n outRaxml -x 12345 -p 12345 -q {input.partitions} -# 100 -f a. Divergence times were acquired via MEGA7 RelTime [268], using a Poisson model: megacc -a reltime_ml_protein.mao -d concat.fasta -t micrurus.nexus -g groups.txt -c Calibration.txt -o timeTree_Calibration. Some parts of the code were written with Snakemake [269] in order to automate it. All code is available on a github repository [270].

## Figures and Tables

**Table 1:** Pharmacology of mammalian muscarinic acetylcholine receptors (mAChRs) compared with actions of mamba muscarinic toxins. mAChRs (blue) comprise five classes (M1-M5) with broad peripheral and central tissue distributions. Ryberg et al. [45] have shown that through its actions on muscarinic receptors, acetylcholine causes vasodilation of rat carotid and submandibular arteries and vasoconstriction of jugular and submandibular veins. The response depends upon the mAChR class to which it binds. Mamba (*Dendroaspis*) muscarinic toxins (red) may act as either agonists or antagonists of mAChRs, but it appears that they agonize vasodilatory mAChRs and antagonize vasoconstrictive receptors. The net result is profound hypotension [46,47]. References for mAChR and ligand pharmacology, and for toxin effects are as follows: M1 [45,48]; M2 [49,50,51,52]; M3 [42,53,54,55]; M4 [45,56]; M5 [57]; m1-toxin/MT7 [33,58,59]; m2-toxin [60]; MT2/MTα/MTβ [35,61]; m4-toxin/MT3/MT2/MT7 [35,59,62]; MTα/MTβ [44].

mAChR Class	Tissue Target	Agent Action	Organism	Effect	Reference	Toxin	Reference	Action	Probable Effect
M1	Rostral ventrolateral medulla	Agonist	Humans	Vasoconstriction, Tachycardia	Medina et al., 1997	m1-Toxin; MT7	Max et al., 1993; Jolkkonen, 1996; Liang et al., 1996	Antagonist	Hypotension
M1	Submandibular & jugular vein endothelium	Agonist	Rats	Vasoconstriction	Ryberg et al., 2008				
M2	Cerebral ventricles	Antagonist	SH rats	Hypotension	Brezenoff et al., 1988	m2-toxin	Carsi et al., 1999	Antagonist	Hypotension
M2	Cerebral ventricles	Agonist	Rats	Hypertension, Tachycardia	Pazos et al., 1986; Kubo, 1998; Ozkutlu et al., 1993				
M2	Cardiac Atrium		Mice	Negative Inotropy	Nishimaru et al., 2000				
M3	Aorta	Agonist	Mice	Vasodilation	Khurana et al, 2004	MT2; MTα; MTβ	Bradley, 2000; Jolkkonen et al., 1995	Agonist	Hypotension
M3	Resistance vessels	Agonist	Mice	Vasodilation	Bruning et al., 1994; Gericke et al., 2011				
M3	Mesenteric vessels	Agonist	Rat	Vasodilation	Hendriks et al., 1992				
M3	Coronary arteries	Agonist	Mice	Vasodilation	Lamping et al., 2004				
M4	Submandibular & jugular vein endothelium	Agonist	Rats	Vasoconstriction	Ryberg et al., 2008	m4-toxin; MT3; MT2; MT7	Jolkkonen et al., 1994; Liang et al., 1996; Bradley, 2000	Antagonist	Hypotension
M4	Spinal Cord	Binding only	Rats	Not determined	Höglund & Baghdoyan, 1997				
M5	Cerebral blood vessels	Agonist	Mice	Vasodilation	Yamada et al., 2001	MTα; MTβ	Jolkkonen et al., 1995	Agonist	Hypotension

**Figure A1:**
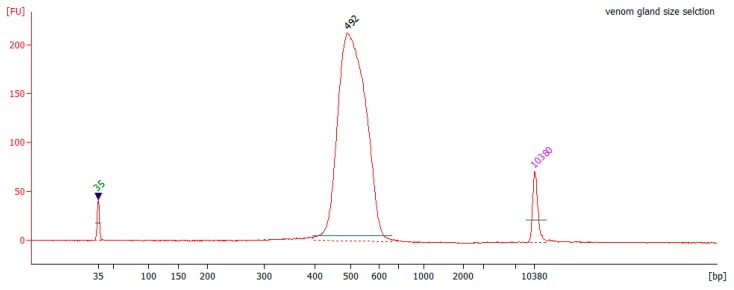
Size selection of pooled cDNA libraries confirmed that they had an average length of ~500 bp.

## Supplementary Materials

The following are available online at www.mdpi.com/2072-6651/9/6/187/s1.

Table S1. Transcriptome sequencing statistics.

Table S2. Venom protein composition for six Brazilian *Micrurus* species.

Table S3. Specimen collection data.

Figure S1. SDS-DTT 4–12% gradient gel (Bis-Tris pH 8.3) confirmed that the abundance of 3FTx in *Micrurus l. carvalhoi* venom suggested by transcriptomics (72.6%) (Table S2) is likely accurate, while the abundance suggested by mass spectrometry (2.3%) (Table 2) cannot possibly be correct. (**A**) The gradient gel itself is shown. Molecular weight markers are shown in the left and right lanes. Venom was loaded in the three center wells: 62.5 µg (left); 125 µg (middle); 187.5 µg (right). Fraction numbers are provided to the right of the most concentrated venom sample. (**B**) Proteins identified in the fractions, after in-gel digestion with trypsin, followed by mass spectrometry. Fraction numbers correspond to those in panel A. 3FTxs were found in Fractions 10–16, where they were significant components. 3FTxs were also found in Fractions 3–6.

Figure S2. *Micrurus* venoms are rich in 3FTxs displaying an astonishing variety of primary structures. Micrurine 3FTxs may possess 8, 9, or 10 Cys residues with different disulfide patterns in each group. Pharmacologies are almost entirely unknown. Some probably target nicotinic acetylcholine receptors of reptilian neuromuscular junctions, but their potential targets in mammals are unknown. Most 3FTx have 21-residue signal peptides, shown to the left of the black vertical line; however, Group 28 is unique in that it has 24-residue signal peptides.

Figure S3. 5′-Nucleotidases dephosphorylate purine mononucleotides so as to release free nucleosides (principally adenosine), which contribute to prey hypotension [46]. All eight *Micrurus* species examined to date have 5′-nucleotidases in their venoms. These are present at levels ranging from ~0–0.05% (Table S2). With 573–588 residues and no signal peptides, micrurine venom 5NUCs are slightly shorter than their crotalid counterparts. They bear two different C-termini, one 14 residues longer than the other. Interestingly, the longer variant also appears in 5NUCs of the pitvipers, *Protobothrops elegans* and *Protobothrops flavoviridis* [11], and *Gloydius brevicaudus* [271]. Micrurine 5NUCS are structurally almost invariant, in addition to their close identity with the crotalid enzymes.

Figure S4. Micrurine acetylcholinesterases (AChEs) are modestly large proteins, containing approximately 585 amino acids. However, they are not expressed at biologically significant levels in *Micrurus* venoms, as they are in some Old World elapid taxa. The primary function of AChE in the venoms of those elapids that produce it, is presumably to starve neuromuscular junctions of transmitter, in concert with postsynaptic antagonists of nicotinic AChRs. Among the *Micrurus* transcriptomes, AChE is most abundant in *M. l. lemniscatus* venom, where AChE transcripts total only 0.05% of the transcriptome (Table S2). Micrurine AChEs are highly variable in primary structure. Some, such as *M. s. spixii* transcript DN97541_c0_g1_i1|m.57626, are predicted to have 29-residue signal peptides, while others, such as *M. tener* AChE (JAS05178.1), are expected to have signal peptides of only 23 residues, with shorter N-termini.

Figure S5. Aminopeptidase A (APA) converts kallidin to bradykinin [82,87], a potent inducer of hypotension. All of the transcriptomes except that of *M. surinamensis* had an APA sequence. The *Micrurus* sequences comprise 958 residues and are largely identical to the APA from *Gloydius brevicaudus* [87]. *Micrurus s. spixii* had the highest APA transcript abundance, but that constituted only 0.09% of the transcriptome (Table S2).

Figure S6. Like APA, Aminopeptidase N (APN) also converts kallidin to bradykinin [82,87]. All of our transcriptomes except for that of *M. paraensis* contained an APN transcript, as did that of *M. fulvius*. All were also incomplete, but were nearly identical to the *M. fulvius* sequence insofar as comparisons could be made. *Micrurus* sequences were also very similar to the *Gloydius brevicaudus* sequence (BAG82599.1; Yuko Ogawa and Ryohei Yanoshita, 2007, unpublished). Among micrurine APNs, that of *M. surinamensis* was the most abundant at 0.01% of its transcriptome (Table S2).

Figure S7. New World elapid C-type lectin-like proteins (CTLs) exhibit tremendous sequence variation, and may comprise as many as 8-10 structural subclasses. Micrurine CTLs may have 6, 7, 8, or 9 Cys residues, and the 7-Cys toxins present two different patterns, raising the possibility of heterodimeric CTLs. However, there have been no pharmacological characterizations of any *Micrurus* CTLs to date. Among our samples, CTLs ranged in abundance from trace quantities in *M. s. spixii* to 1.87% in *M. paraensis* and 3.35% in *M. corallinus*. These significant quantities suggest a role of some importance, at least in the latter two species. Signal peptide sequences are shown to the left of the heavy black vertical line.

Figure S8. Various crotalid CRISPs act as L-type Ca^2+^ channel antagonists of depolarization-induced arterial smooth muscle contraction [97]; thus, they promote vasodilation and hypotension. All six *Micrurus* transcriptomes contained CRISP sequences of 221 residues plus 19-residue signal peptides (left of black line). The sequences from *M. l. carvalhoi*, *M. l. lemniscatus*, and *M. surinamensis* are identical over the first 201 residues, at which point they terminate. We do not know whether they are complete or truncated. The sequences of *M. corallinus* and *M. s. spixii* differ somewhat from the three foregoing, but that of *M. paraensis* is radically different, being more similar to the CRISP from *Ovophis okinavensis* venom [10]. Micrurine CRISPs range from trace levels in most species to 0.24% of the *M. l. lemniscatus* transcriptome (Table S2).

Figure S9. Micrurine CRISP-EGFs appear to comprise three protein subclasses, one of which includes CRISP-EGF transcripts from *Micrurus fulvius* and crotalid venoms and from colubrid tissues. This first subclass has 28-residue signal peptides. The second and third subclasses consist only of micrurine sequences from the present study. The second subclass is most closely related to an uncharacterized protein from the *Protobothrops mucrosquamatus* genome and a CRISP with an EGF domain from the *Thamnophis sirtalis* genome. Both of these proteins exceed 960 amino acids. The third subclass is represented only by two pairs of incomplete sequences, both of which appear related to fibulins. All four of these are expressed at trace levels (<0.0002%); thus, they may be contaminating tissue proteins.

Figure S10. Cystatins have not been reported previously as snake venom constituents and we make no claim that they are. However, they are present in the *Micrurus* venoms examined here, although in only trace quantities (0–0.02% of the transcriptomes). Micrurine cystatins possess 24-residue signal peptides and have their Cys residues in the same positions as mammalian cystatins, with which they share only 45-55% of their amino acids. However, it appears that they probably are secreted proteins.

Figure S11. All six *Micrurus* transcriptomes contained a DPP IV transcript and that of *M. surinamensis* contained two. DPP IVs comprise 752–779 amino acids, but because they are membrane-bound in microsomes [117], they have no signal peptides. One sequence from *M. surinamensis* has a 27-residue insert that is lacking in the other homologs.

Figure S12. Aird et al. [10] postulated that the primary function of galactose-binding lectins (GBLs) may be to upregulate venom synthesis in the venom gland when venom supplies become depleted. However, with the present findings, that view may have to be revised somewhat. While several species in the present study also have typically low GBL titers (0–0.19%)(Table S2), *M. paraensis* (1.93%) and *M. corallinus* (3.35%) have the highest levels reported in any snake venom to date, suggesting that in these two species, GBL hemagglutinating and edematogenic properties [122] may play a significant role in envenomation. Signal peptides are shown to the left of the vertical black line.

Figure S13. Guanylyl cyclase (GC) is believed to serve only the function of cyclizing N-terminal glutamine residues of specific venom proteins to prevent their rapid degradation in the host. For this reason, GC is not a snake venom constituent in the usual sense, although it is usually present in trace quantities. All six coral snake transcriptomes possessed identical 368-residue transcripts with a putative 23-residue signal peptide (left of black vertical line) that comprised from 0–0.02% of their respective transcriptomes. Even the GC transcript of the habu, *Protobothrops flavoviridis*, differed from the micrurine GCs at only 5 residues.

Figure S14. Hyaluronidases are generally minor enzymatic components of snake venoms. They have been recorded to date in 10 *Micrurus* venoms, including all six species that we studied. Micrurine hyaluronidases comprise 465 amino acids and lack signal peptides. In our transcriptomes, hyaluronidases accounted for between ~0% (*M. s. spixii* and *M. l. lemniscatus*) and 0.27% (*M. surinamensis*) of the transcriptomes. Consistent with the finding of Aird et al. (2015) that the most abundant venom proteins evolve most rapidly, hyaluronidases show strikingly little sequence variation. Most *Micrurus* species apparently have only one hyaluronidase transcript, but oddly, *M. l. carvalhoi* had three.

Figure S15. In addition to typical elapid Kunitz serine protease inhibitors (short KSPIs, 59–61 residues), some *Micrurus* venoms contain novel KSPIs (long KSPIs) 232 residues in length. The physiological targets of both classes are unknown. The long KSPIs should not be confused with SERPINs, which are nearly twice again as long (Figure S26). Signal peptides of either 20 or 24 residues are shown to the left of the black vertical line.

Figure S16. Four *Micrurus* species possess long KSPIs (232 residues) that appear to have arisen through gene duplication of a short KSPI, with the duplicated portion being grafted onto the C-terminus of a short KSPI. The long KSPIs have 13 Cys residues, but the disulfide pattern is unknown; however, the second group of six cysteines has the same spacing as the first six (See also Figure S15). In this figure, the C-terminal half of the sequences has been cut and re-aligned with the N-terminal half to illustrate the Cys pattern match.

Figure S17. The flavin-enzyme, L-amino acid oxidase (LAO), is responsible for the bright yellow color of many solenoglyph venoms, in which LAO content can approach 10% [10]. Snake venom L-amino acid oxidase (EC 1.4.3.2) generates H_2_O_2_ from the conversion of L-amino acids to keto acids. It is a more minor constituent of *Micrurus* venoms, ranging from essentially 0% (*M. s. spixii*) to 0.84% (*M. corallinus*). Micrurine LAOs exhibit relatively little sequence variation, except at specific residues, where the same types of amino acid substitutions are encountered across species.

Figure S18. Most *Micrurus* venoms appear to contain only one Type III metalloprotease, comprising 614 residues, including a 20-residue signal peptide (left of the vertical black line). One *M. l. lemniscatus* transcript (lemniscatus DN101949_c32_g1_i4|m.13925) yields a mature protein with a mass of 66,657 D and a predicted pI of 8.37. This protein is most similar to atragin, from the venom of *Naja atra* [137]. The metalloprotease content of the venoms we examined varies from essentially 0% in *M. s. spixii* to 2.1% in *M. corallinus*. The latter suggests a significant function in envenomation. Nonetheless, the pharmacological and physiological effects provoked by micrurine metalloproteases are unknown.

Figure S19. Natriuretic peptide transcripts were found in all six of the *Micrurus* species we investigated. They have also been reported in venom gland transcriptomes of *M. fulvius* and *M. altirostris* as well. Interestingly, two of our *M. l. carvalhoi* transcripts show evidence of gene duplication. One of them contains at least three copies of an NP. The sequence of human CNP is shown across the bottom for comparison. NP transcripts have 25-residue signal peptides (left of the vertical black line), but we cannot draw any conclusions regarding the total length of the transcripts. Solid brown lines separate the repeats.

Figure S20. Micrurine nerve growth factor (NGF) transcripts encode 227 amino acids plus 18-residue signal peptides. They are highly similar sequences, displaying numerous blocks of invariant residues. Most variable positions show specific sets of substitutions, synonymous and non-synonymous, that occur without regard to species. The only strongly divergent sequences include a set of six transcripts from *M. l. lemniscatus*, which vary at numerous positions. Another group of four transcripts from *M. l. lemniscatus* and *M. corallinus* are also significantly different from all others; however, the significance of these variations, if any, is unknown.

Figure S21. All of the *Micrurus* venoms investigated here apparently have a single phosphodiesterase gene, except for *M. surinamensis*, which barring a transcript misassembly, may have two. The transcript in question shows an inserted glycine at position 700, and a glutamate residue at position 698, where all other sequences have leucine. Signal P predicts signal peptides of 18 residues, but they are probably actually 22 residues long, given the unbroken 14-residue run of aliphatic amino acids. PDE sequences are highly conserved, given the similarity of *Micrurus* sequences to *Protobothrops* sequences (Old World crotalids). PDEs are expressed at trace levels in five of the *Micrurus* species and at 0.02% in *M. corallinus*.

Figure S22. Structures of micrurine phospholipases A_2_, illustrating their great structural diversity. Of 244 PLA_2_s with partial or complete structures, 202 are apparently catalytic, having the requisite H48, D49, Y52, and D101 in their active sites. The remaining 42 are apparently non-catalytic. Positions indicated in red across the bottom designate residues involved in Ca^2+^-binding [172]. Positions indicated in blue are catalytic residues. The anticoagulant site [173] and the neurotoxic hydrophobic region [172,174] are indicated by purple and green bars, respectively. Residues proposed by Alape-Girón et al. [175] to be involved in myotoxicity of elapid PLA_2_s are indicated below with black cells. Residues participating in the hydrophobic channel, via which substrate enters the catalytic site are indicated in gray [176]. PLA_2_s from the two North American species, *M. fulvius* and *M. tener*, form a structural cluster that is relatively distinctive from the much more variable South American sequences, which probably also represent much greater pharmacological diversity. SignalP predicted signal peptides of 21 residues; however, elapid PLA_2_s sequenced by Edman degradation commence with residue 28, confirming that the signal peptides are actually 27 residues in length. Numbers of cysteine residues are given for sequences that are complete. Numbers of cysteines in parentheses are for sequences truncated at the N-terminal end, for which the cysteines can be reliably predicted, owing to the relative invariability of the N-termini. Asterisks indicate stop codons.

Figure S23. A phospholipase B (PLB) transcript was found in each of the six *Micrurus* species examined. PLB is believed to contribute to envenomation by hydrolyzing membrane phospholipids [224].

Figure S24. RNAse is essentially absent in all of the transcriptomes, except for that of *M. surinamensis*, where it comprises 0.7% of the transcriptome. Micrurine RNAse transcripts have 141 residues and apparently lack signal peptides. The sequences are largely invariant, showing amino acid substitutions, all non-synonymous, at only 3 positions (V/D60; R/G63; F/S85). Oddly, 3 *M. l. carvalhoi* transcripts and both transcripts from *M. l. lemniscatus* have stop codons at position 118. The functional significance of this truncation is unknown. The loss of the C-terminal 24 residues suggests that that these have become pseudogenes, since the next 17 residues after the stop codon are the same as in full-length RNAse transcripts. However, we cannot exclude the possibility that these apparently dysfunctional enzymes have adopted another function instead.

Figure S25. A single serine protease transcript of 265 residues was found in each of the six *Micrurus* venoms examined in the present study. The signal peptide accounts for the first 18 residues. Except for their signal peptides, these protease sequences align well with the transcripts published from *M. tener* venom. Two serine protease transcripts from the transcriptome of *M. fulvius* do not appear closely related to the other seven. Given the great similarity of the micrurine serine proteases presented here, the *M. fulvius* sequences may represent contaminating blood or tissue serine proteases. The functions of micrurine serine proteases are unknown.

Figure S26. Serpins consist of 501–504 residues plus a 19-residue signal peptide (left of vertical black line). They are approximately 6x longer than KSPIs (Figure S15). In *Micrurus* venom glands, these transcripts are almost invariant, showing amino acid substitutions at only about 24 positions. Serpins constitute 0–0.04% of the transcriptomes examined in this study. Their probable role in envenomation is to contribute to anticoagulation.

Figure S27. Micrurine long VEGFs (VEGF-Cs) are nearly structurally invariant. VEGF-C mRNA transcripts comprise 399 residues, with a signal peptide of uncertain length. SignalP v4.1 predicts 16-residue signal peptides (dashed vertical line), but this would leave a long, hydrophobic N-terminus. While that is possible, a 24-residue signal peptide would seem more reasonable (solid vertical black line). No VEGF-C has been reported from *Micrurus tener* venom, which does have a short VEGF.

Figure S28. Short VEGFs (VEGF-Fs) are conservative in amino acid composition, but display at least three major structural variants. Position 142 may be N or K, or missing altogether in one transcript from *M. corallinus*. Toxins with K142 then have an extremely basic 24-residue insert, with 14 K or R residues from positions 142–162. The majority of VEGF-Fs have a hydrophilic 25-residue C-terminal sequence, commencing with a short hydrophobic segment, YLHLL. However, three toxins terminate with a short hydrophilic sequence, CEKPRR. It is unclear whether sequences 9–13 are truncated or complete sequences. VEGF-Fs were present in all six of our transcriptomes. They have 26-residue signal peptides (vertical black line).

Figure S29. Ohanin, the first known vespryn, which was isolated from the venom of the king cobra (*Ophiophagus hannah*), produces hypolocomotion and hyperalgesia in mice [239]. Its effects in snakes may be similar, but at present, no pharmacological data are available. All six Brazilian *Micrurus* venoms possessed single vespryn sequences, except for the venoms of *M. paraensis* and *M. l. carvalhoi*, in which there were two. *Micrurus* vespryns are nearly identical to ohanin and display limited sequence variation. Micrurine vespryn transcripts comprise 190 residues and lack signal peptides. Vespryns appear at trace levels in most of the venoms examined here, although they comprised 0.22% of the *M. s. spixii* transcriptome and 1.66% in *M. corallinus*.

Figure S30. Micrurine waprin-like sequences are more similar to opisthoglyph colubrid and crotalid waprin-like sequences than to those of Old World elapids. Waprin-like sequences have been reported from all *Micrurus* venoms except that of *M. fulvius*. Colubrid and crotalid waprin-like proteins have 23-residue signal peptides (left of vertical black line). None of our sequences have complete signal peptides and the sequence from *M. tener* is so different at the N-terminus that no definite conclusions can be drawn regarding it length in *Micrurus*. However, it appears that all micrurine waprin-like sequences have an N-terminal Q, as in the colubrid and crotalid sequences.

## Data Accessibility

Transcriptomic sequences have been deposited in the DNA Databank of Japan (DDBJ) under the project ID: PRJDB5628. BioSample IDs are: SAMD00076719-SAMD00076724, and raw data may be accessed under ID: DRA005678 (DRX083388-DRX083393).

## Appendix A

### Appendix A.1. First Strand cDNA Synthesis

RNA was isolated using the Trizol method and was purified with Qiagen RNeasy Micro Kits. One μg of total RNA was used for first strand cDNA synthesis. ERCC ExFold RNA Spike-In Mix (Life Technologies, Carlsbad, CA, USA) was included in the cDNA synthesis reaction according to the manufacturer’s protocol. To the RNA and spike-in mix, 1 μL of 10 μM poly T_START oligo.

(5′-AATTGCAGTGGTATCAACGCAGAGCGGCCGCTTTTTTTTTTTTTTTTTTTTTTTTTTTTTVN) was added. The 10-μL reaction was incubated at 65 °C for 3 min, and then chilled on ice. To this, a 10-μL mixture, containing 4 μL of 5x first strand synthesis buffer (Invitrogen, Carlsbad, CA, USA), 1 μL of 10 mM dNTP (Promega, Madison, WI, USA), 2 μL of 0.1 M DTT (Invitrogen), 1 μL of 12 μM template-switching RNA oligo. (5′-AAGCAGUGGUAUCAACGCAGAGUACAUGGG), 1 μL RNase inhibitor (Qiagen), and 1 μL Superscript II Reverse Transcriptase (Invitrogen) was added to each sample. Reactions were incubated at 42 °C for 60 min and the enzyme was heat-inactivated at 65 °C for 15 min. 80 μL MilliQ water were added to each cDNA reaction.

### Appendix A.2. Second Strand cDNA Synthesis

Second strand cDNA was synthesized with 9 PCR cycles. The 50-μL PCR reaction consisted of 1x Phusion HF buffer (Thermo Scientific, Waltham, MA, USA), 200 μM dNTP (Promega), 0.5 μM START primer (5′-CGCCAGGGTTTTCCCAGTCACGACAATTGCAGTGGTATCAACGCAGA), 0.5 μM TS_long primer (5′-CTTGTAGGTTAAGTGGAGAGCTAACAATTTCACACAGGAAAGCAGTGGTATCAACGC), 0.5 μL of 2 U/μL Phusion DNA polymerase (Thermo Scientific), and 5 μL diluted cDNA. Eight 50-μL PCR reactions were set up for each cDNA sample. PCR was carried out with the following conditions: initial denaturation at 98 °C for 30 s, with 9 cycles of denaturation at 98 °C for 10 s, 68 °C for 6 min, followed by final extension at 72 °C for 10 min. PCR reactions were concentrated with an Amicon Ultra-0.5 column (Millipore, Billerica, MA, USA).

Concentrated PCR products were purified by solid phase reversible immobilization using Dynabeads MyOne Carboxylic Acid (Invitrogen). 18% PEG was used for purification. 1 ng of ds-cDNA was used library preparation and sequencing, using a Nextera XT DNA sample kit (Illumina, San Diego, CA, USA) according to manufacturer’s directions. Twelve cycles of PCR were used for library amplification.

### Appendix A.3. Size Selection of the Pooled cDNA Libraries

Libraries were pooled so that all six contained the same amount of cDNA. Pooled libraries were then size-selected by gel extraction to achieve an optimal insert size for the Illumina Hiseq PE150 (~500 bp). Size distribution of the library was confirmed using an Agilent 2100 BioAnalyzer (Figure A1).

### Appendix A.4. Reduction and Alkylation of Snake Venoms Prior to Enzymatic Digestion

Crude venoms were gently vortexed for several seconds to suspend microsomes. Reduction was accomplished by adding 36 µL 8 M urea, 2 µL venom (125 µg/µL), 2 µL 500 mM DTT in ultrapure water, and 10 µL 500 mM of ammonium bicarbonate to 200 µL PCR tubes. Tubes were incubated at 60 °C for 45 min in an MJ PTC 200 Peltier thermocycler. Then, 4 µL 500 mM sodium iodoacetate were added to each tube and mixed gently. Tubes were incubated at 37 °C for 30 min in the dark. Afterward, an additional 2 µL 500 mM DTT were added to quench the alkylation reaction. Sample desalting was done by pipetting the reaction from the PCR tubes into 10K Nanosep centrifugal filters (Pall Corp., New York, NY, USA) and centrifuging them at 18,407× *g* for 25 min. Samples were rehydrated with 50 µL of 50 mM ammonium bicarbonate and were again centrifuged to further desalt the samples.

### Appendix A.5. Tryptic Hydrolyses

After the second centrifugation, 10 µL 8 M urea were added to the membrane and the membrane surface was wetted with the urea by rotating the tube manually. Then 90 µL 50 mM ammonium bicarbonate were added to the membrane and the filter was vortexed gently to re-suspend desalted proteins. Solutions were pipetted from the filter and placed in clean 200 µL PCR tubes. Finally, 10 µg of trypsin (Pierce 90055) dissolved in 10 µL 1 mM HCl were added to each tube. Tubes were incubated 24 h at 37 °C. Then, samples were pipetted back into their original 10 K Nanosep filters and centrifuged again at 18,407× *g* for 30 min to collect the de-proteinated filtrate which was frozen at −30 °C until analysis by mass spectrometry.

### Appendix A.6. Chymotryptic Hydrolyses

The procedure for chymotrypsin (Pierce 90056) was nearly identical to that for trypsin, except that after wetting the Nanosep filter with 10 µL 8 M urea, 10 µL 50 mM ammonium bicarbonate, 5 µL 200 mM CaCl_2_, and 65 µL ultrapure water were added to the membrane. All subsequent procedures were identical to those for trypsin.

### Appendix A.7. Formic Acid Hydrolyses

Crude venoms were gently vortexed for few seconds. Digestion of proteins was done by mixing 2 µL venom, 4 µL 500 mM DTT in ultrapure water and 94 µL 2% formic acid (Wako 063-04192) in 200 µL PCR tubes. Tubes were incubated for 2 h at 99 °C and then frozen at −30 °C until analysis by mass spectrometry.

### Appendix A.8. Digestion and Extraction of Peptides from SDS PAGE Gels

Gel bands were excised in order of decreasing molecular weight. Each fraction was reduced at 56 °C with 10 mM dithiothreitol in 50 mM ammonium bicarbonate for 10 min, and then alkylated with 55 mM iodoacetamide in 50 mM ammonium bicarbonate for 20 min in the dark. Samples were washed with 50 mM ammonium bicarbonate in 50% acetonitrile, followed by acetonitrile. After washing, each fraction was digested with trypsin overnight at 37 °C. Peptides were extracted from excised gel bands using 5% formic acid and 50% acetonitrile in water. After extraction, peptides were concentrated with a centrifugal vacuum concentrator and then resuspended in 0.1% formic acid in water.
