# Addendum: Aird, S.D. et al. Coralsnake Venomics: Analyses of Venom Gland Transcriptomes and Proteomes of Six Brazilian Taxa. *Toxins* 2017, *9*(6), 187

**DOI:** 10.3390/toxins10050172

**Published:** 2018-04-24

**Authors:** Steven D. Aird, Nelson Jorge da Silva, Lijun Qiu, Alejandro Villar-Briones, Vera Aparecida Saddi, Mariana Pires de Campos Telles, Miguel L. Grau, Alexander S. Mikheyev

**Affiliations:** 1 Division of Faculty Affairs, Okinawa Institute of Science and Technology Graduate University, 1919-1 Tancha, Onna-son, Kunigami-gun 904-0495, Okinawa-ken, Japan; 2 Ecology and Evolution Unit, Okinawa Institute of Science and Technology Graduate University, 1919-1 Tancha, Onna-son, Kunigami-gun 904-0495, Okinawa-ken, Japan; lijun.qiu@oist.jp (L.Q.); miguel.graulopez@oist.jp (M.L.G.); alexander.mikheyev@oist.jp (A.S.M.); 3 Programa de Pós-Graduação em Ciências Ambientais e Saúde, Pontifícia Universidade Católica de Goiás, Goiânia 74605-140, Goiás, Brazil; nelson.jorge.silvajr@gmail.com (N.J.d.S.J.); verasaddi@gmail.com (V.A.S.); tellesmpc@gmail.com (M.P.d.C.T.); 4 Research Support Division, Okinawa Institute of Science and Technology Graduate University, 1919-1 Tancha, Onna-son, Kunigami-gun 904-0495, Okinawa-ken, Japan; avillar@oist.jp; 5 Laboratório de Oncogenética e Radiobiologia da Associação de Combate ao Câncer em Goiás, Universidade Federal de Goiás, Rua 239 No. 52—Setor Universitário, Goiânia 74065-070, Goiás, Brazil; 6 Laboratório de Genética & Biodiversidade, Universidade Federal de Goiás, Goiânia 74690-900, Goiás, Brazil

Following publication of this paper, Dr. Daniel Dashevsky discovered to our chagrin, that the transcriptomic datasets uploaded to the DNA Databank of Japan (DDBJ) contained numerous complete 3FTx sequences that were not included in our paper. A protracted investigation determined that owing to their brevity, protein predictions of many 3FTx sequences and a small number of PLA_2_ sequences were rejected by Transdecoder.

Since all sequences from the study have been in the public domain since the outset, the purpose of this addendum is simply to add the previously unpublished sequences to the paper. These do not alter our previous data analysis, and no additional analysis is included. The Figures S1–S5 herein compare all of the complete 3FTx and PLA_2_ sequences identified in this study with previously published sequences. Duplicate transcripts, fragments, and partial sequences are not included.

New World coralsnake venoms contain an astonishing variety of three-finger toxins (3FTxs), including toxins with novel disulfide bond arrangements (see main text). These toxins include forms with 8, 9, 10, and 11 cysteines. The six Brazilian *Micrurus* taxa investigated here invest more heavily in 3FTxs than in PLA_2_s. 3FTxs were identified using Megablast in Geneious 8.1.9. Multiple 3FTx sequences with signal peptides, representing all cysteine patterns discovered to date, were used as query sequences in an effort to avoid overlooking any complete sequences. Sequences were aligned using MUSCLE, and alignments were then further refined by eye. 3FTx sequences are presented here without signal peptides according to cysteine class, and they are also compared with sequences pre-existing in the literature.

## Supplementary Materials

The following are available online at http://www.mdpi.com/2072-6651/10/5/172/s1, Figure S1: Three disulfide bond patterns are evident among 8-cysteine 3FTxs from the venoms of *Micrurus corallinus*, *M. l. carvalhoi*, *M. l. lemniscatus*, *M. paraensis*, *M. spixii*, and *M. surinamensis*. The first pattern is illustrated by sequences 1–12, and includes the overwhelming majority of 8-Cys toxins. The second, represented by sequences 13–19, occurs in *M. l. carvalhoi*, *M. paraensis*, *M. lemniscatus*, and *M. surinamensis*. These toxins have four cysteines among the N-terminal 18 amino acids, like γ-bungarotoxin (positions 3, 6, 11, and 17), and they lack the two C-terminal cysteines (positions 65 and 70), including one of the usual paired residues. The third pattern is represented by sequences 57–60 and 92. In these toxins, the paired cysteines have become separated, with Cys-66 being displaced two residues C-terminally, such that the three C-terminal cysteines occur in positions 64, 67, and 70, instead of 64, 65, and 70. The functional significance of these patterns is unknown. A tree was constructed using the Neighbor-Joining method with the Jukes-Cantor model and γ-bungarotoxin from *Bungarus multicinctus* venom as an outgroup. Asterisks indicate stop codons, which were paired in some cases; Figure S2: 3FTxs having 9 cysteines, derived from venoms of *Micrurus corallinus*, *M. l. carvalhoi*, *M. l. lemniscatus*, *M. paraensis*, *M. spixii*, and *M. surinamensis*, display two disparate disulfide bond patterns. The first, representing the majority of 9-Cys toxins, has paired second and third cysteines in positions 16–17 (sequences 1–28 and 30–31). In the second group, the cysteine in position 16 is absent and instead a cysteine is present in position 63. Whether the extra cysteine is free in monomeric toxins, or whether these 9-Cys toxins form homodimers, or heterodimers with a non-homologous toxin is unknown. γ-bungarotoxin from *B. multicinctus* venom was used as an outgroup. Asterisks indicate stop codons; Figure S3: Venoms of *Micrurus corallinus*, *M. l. carvalhoi*, *M. l. lemniscatus*, *M. paraensis*, *M. spixii*, and *M. surinamensis* all have 3FTxs with 10 cysteines and our samples of *M. l. carvalhoi* and *M. spixii* also have novel toxins with an eleventh cysteine occurring at either position 32 or 37. The functional significance of these patterns is unknown. A tree was constructed using the Neighbor-Joining method with the Jukes-Cantor model and γ-bungarotoxin from *B. multicinctus* venom as an outgroup. Asterisks indicate stop codons; Figure S4: South American *Micrurus* 3FTx sequences comprise as astonishing variety of structural subtypes, including many that have not been reported from the North American species, *M. fulvius* and *M. tener*. However, some subtypes are common to both groups. Previously published 3FTx sequences were downloaded from the NCBI nr database. γ-bungarotoxin from *B. multicinctus* venom was used as an outgroup. Asterisks indicate stop codons; Figure S5: The *Micrurus* taxa investigated here rely much less heavily on PLA_2_ toxins than do the North American taxa, *M. fulvius* and *M. tener*. Still, various structural subclasses are evident, although differences in function cannot be surmised at present, except for probable non-catalytic PLA_2_s that lack the active site His-58 and/or Asp-59 residues (see main text). Most of the North American sequences cluster separately from the Brazilian sequences. PLA_2_ sequences from Brazilian *Micrurus* species were identified with Megablast, using nine complete PLA_2_s, including signal peptides, as queries. 35 Brazilian *Micrurus* PLA_2_s are aligned here with 169 published sequences, using Geneious software. A tree was constructed using the Neighbor-Joining method with the Jukes-Cantor model, with *Bungarus fasciatus* PLA_2_ BF-32 as an outgroup. Signal peptides are not shown in this alignment. Asterisks indicate stop codons.

