# Supplementary material for: Coralsnake Venomics: Analyses of Venom Gland Transcriptomes and Proteomes of Six Brazilian Taxa
Source: Toxins (Basel). 2017 Jun 8;9(6):187. doi: 10.3390/toxins9060187 (PMC5488037; doi:10.3390/toxins9060187)
Supplement: Supplementary file 1 [file toxins-09-00187-s001.pdf]

Supplementary Materials: Coralsnake Venomics: Analyses of Venom Gland Transcriptomes and Proteomes of Six Brazilian Taxa

Steven D. Aird, Nelson Jorge da Silva, Jr., Lijun Qiu, Alejandro Villar-Briones, Vera Aparecida Saddi, Mariana Pires de Campos Telles, Miquel L. Grau and Alexander S. Mikheyev

Table S1. Transcriptome sequencing statistics.

Statistic	M. l. carvalhoi	Micrurus l. lemniscatus	M. s. spixii	M. corallinus	M. paraensis	M. surinamensis
Number of contigs	134,807	265,717	196,839	182,032	139,609	132,788
Sum of contig length (Mbp)	70	150	109	98	70	73
Maximum contig length (bp)	12,977	42,751	11,326	23,238	11,367	15,233
Mean contig length (bp)	505.122	559.03	542.045	531.407	494.262	540.241
N50 contig length (bp)	1,275	1,511	1,496	1,293	1,191	1,470
# contigs ≥ 1,000 bp	14,261	30,935	22,434	20,124	14,299	15,894
# contigs ≥ 5,000 bp	214	1,215	710	336	128	392
% reads assembled	95.73	87.68	94.53	94.44	96.06	94.72

Table S2. Venom protein composition for six Brazilian *Micrurus* species. Each venom was digested with trypsin and chymotrypsin, and cleaved with formic acid. Resulting peptides were identified and quantified with mass spectrometry. Qualitatively, mass spectrometry generally confirmed the presence of all but the most minor components in the transcriptomes. However, unlike our recent studies of Asian pitviper transcriptomes and proteomes, which showed strong quantitative correlations between transcriptome and proteome, with these *Micrurus* venoms, the correlation was poor. This was in large part due to the fact that owing to the rarity of *Micrurus* and the difficulty of extracting significant quantities of venom from them, glandular mRNA and venom samples were derived from different specimens. Two samples of *M. surinamensis* venom were examined from Belo Monte, Pará and Estreito, Maranhão. These two localities are separated by about 700 km. It is not clear how much to make of quantitative differences between them.

**Toxin Family**	***M. l. carvalhoi* T %**	***M. l. carvalhoi* P %**	***M. corallinus* T %**	***M. corallinus* P %**	***M. l. lemniscatus* T %**	***M. l. lemniscatus* P %**	***M. paraensis* T %**	***M. paraensis* P %**	***M. spixii* T %**	***spixii* P %**	***M. surinamensis* T %**	**Altamira *M. surinamensis* P %**	**Estreito *M. surinamensis* P %**
3FTx	72.59	2.25	20.30	34.83	67.22	34.33	68.56	12.31	62.55	9.48	60.24	90.10	36.84
5NUC		0.00	0.03			0.01	0.05	0.24	0.01	0.03	0.01		
AChE		0.18			0.05	0.32			0.01	0.19			
APA									0.09				
APN		0.01		0.34				0.02		0.00	0.01		0.83
CRISP		0.01	0.01	8.51	0.24								
CRISP-EGF	0.01		0.02		0.04						0.13		
CTL	0.13	2.64	3.35		0.29	2.59	1.87	4.95		7.37	0.17	0.32	1.66
CVF				0.01									
CYST	0.01	0.00			0.02						0.01	0.00	0.11
DPP IV					0.01						0.01		
GBL	0.14		3.35		0.19		1.93				0.17		
GC		0.09		0.00	0.02	0.16		0.04			0.02	0.01	0.27
HYAL	0.18	0.49	0.08	0.46		0.66	0.04	0.26		0.01	0.27	0.17	0.32
KSPI	10.65	12.61	0.16	0.46	7.15	11.69	0.76	3.43	0.13	0.74	1.07	2.55	25.26
LAO	0.10	2.01	0.84	5.42	0.73	3.48	0.15	3.13		4.78	0.05	0.19	0.32
MP P-III	0.22	6.29	2.10	10.38	1.12	4.93	0.34	7.97		6.57	0.14	0.11	1.23
NP	0.41		29.53		0.20		0.82				2.67		
NGF	0.23	1.07	1.12		1.12	1.09	0.26	1.42			7.88		0.17
PDE			0.02	0.08				0.02					
PLA_2	15.29	48.56	37.72	38.54	17.28	19.38	24.91	65.89	36.44	64.04	26.23	6.56	32.98
PLB	0.01	0.14	0.59	0.93	0.13		0.26	0.34	0.18	0.34	0.07	0.01	0.00
RNAse A											0.73		
Serpin					0.04				0.01		0.02		
SP		2.12			0.10	11.43		0.00					

VEGF	0.01	0.02	0.13	0.04	0.31		0.01		0.11		0.01		
VESP		21.51	0.01		1.66	9.32	0.01		0.24	3.73	0.01		
WAPR			0.33		0.45	0.62	0.01		0.01	2.70	0.06		
Total	99.98	100.00	99.69	100.00	98.35	100.00	99.97	100.00	99.76	100.0 0	99.99	100.00	100.00

Following rehydration in RNA Stable, RNA quality of total RNA samples was assessed using a 2100 Bioanalyzer (Agilent Technologies). In some cases, if RIN scores were poor, more than one sample of RNA from the same species was analyzed and the best sample was used. One sample for each of the six species was selected, based on RNA quality scores (Table S1).

Table S3. Specimens and their collection localities.

Species	Transcriptome Specimens	Localities	RIN Scores	Proteome Specimens	Localities
Micrurus carvalhoi	CEPB 9045	Cana Brava, Goiás	8.6	Unknown	No Locality
Micrurus corallinus	CEPB 8875 E	Niterói, Rio de Janeiro	7.4	Instituto Vital Brazil	Niterói, Rio de Janeiro
Micrurus lemniscatus	BME 13807	Altamira, Pará	8.0	No number	Altamira, Pará
Micrurus paraensis	BM 91790	Altamira, Pará	8.4	BME RI 64566	Altamira, Pará
Micrurus spixii	BM 62526	Altamira, Pará	8.7	BME RI 22843	Altamira, Pará
Micrurus surinamensis	BM 83917	Altamira, Pará	8.3	BME-RI-27145	Altamira, Pará
					Estreito, Tocantins

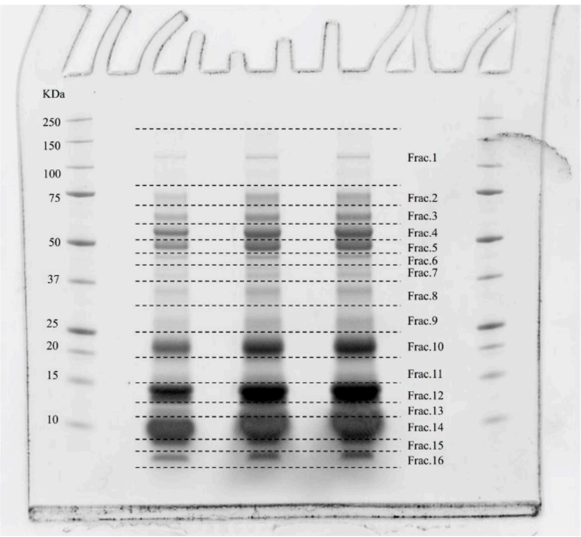

Band #	Proteins Detected
**1**	AChE, APA, APN, HYAL, LAO, PLA_2
**2**	5NUC, AChE, HYAL, ALO, MP-III, PLA_2
**3**	3FTx, 5NUC, AChE, HYAL, ALO, MP-III, PLA_2
**4**	3FTx, 5NUC, AChE, HYAL, KSPI, LAO, MP-III, PLA_2
**5**	3FTx, 5NUC, AChE, GC, HYAL, LAO, MP-III, SP
**6**	3FTx, 5NUC, AChE, GC, HYAL, LAO, MP-III, PLA_2, SP
**7**	AChE, GC, HYAL, LAO, MP-III, PLA_2, SP
**8**	3FTx, AChE, GC, HYAL, LAO, MP-III, NGF, PLA_2, SP
**9**	AChE, CTL, GC, HYAL, KSPI, LAO, MP-III, NGF, PLA_2, SP, VEGF
**10**	3FTx, AChE, CRISP, CTL, CYST, GC, HYAL, KSPI, LAO, MP-III, NGF, PLA_2, SP, VEGF
**11**	3FTx, AChE, CTL, HYAL, KSPI, LAO, MP-III, NGF, PLA_2, VEGF
**12**	3FTx, CTL, CYST, HYAL, KSPI, LAO, MP-III, NGF, PLA_2, VEGF
**13**	3FTx, AChE, CYST, HYAL, KSPI, LAO, MP-III, NGF, PLA_2, SP, VEGF
**14**	3FTx, KSPI, LAO, MP-III, PLA_2, VEGF
**15**	3FTx, HYAL, KSPI, LAO, MP-III, PLA_2, SP
**16**	3FTx, 5NUC, HYAL, KSPI, LAO, MP-III, PLA_2, VEGF

Figure S1. SDS-DTT 4-12% gradient gel (Bis-Tris pH 8.3) confirmed that the abundance of 3FTx in *Micrurus l. carvalhoi* venom suggested by transcriptomics (72.6%) (Table S2) is likely accurate, while the abundance suggested by mass spectrometry (2.3%) (Table 2) cannot possibly be correct. The gradient gel itself is shown. Molecular weight markers are shown in the left and right lanes. Venom was loaded in the three center wells: 62.5 μg (left); 125 μg (middle); 187.5 μg (right). Fraction numbers are provided to the right of the most concentrated venom sample. Proteins identified in the fractions, after in-gel digestion with trypsin, followed by mass spectrometry are identified in tabular form. Fraction numbers correspond to those in the gel photograph. 3FTxs were found in Fractions 10-16, where they were significant components. 3FTxs were also found in Fractions 3-6.

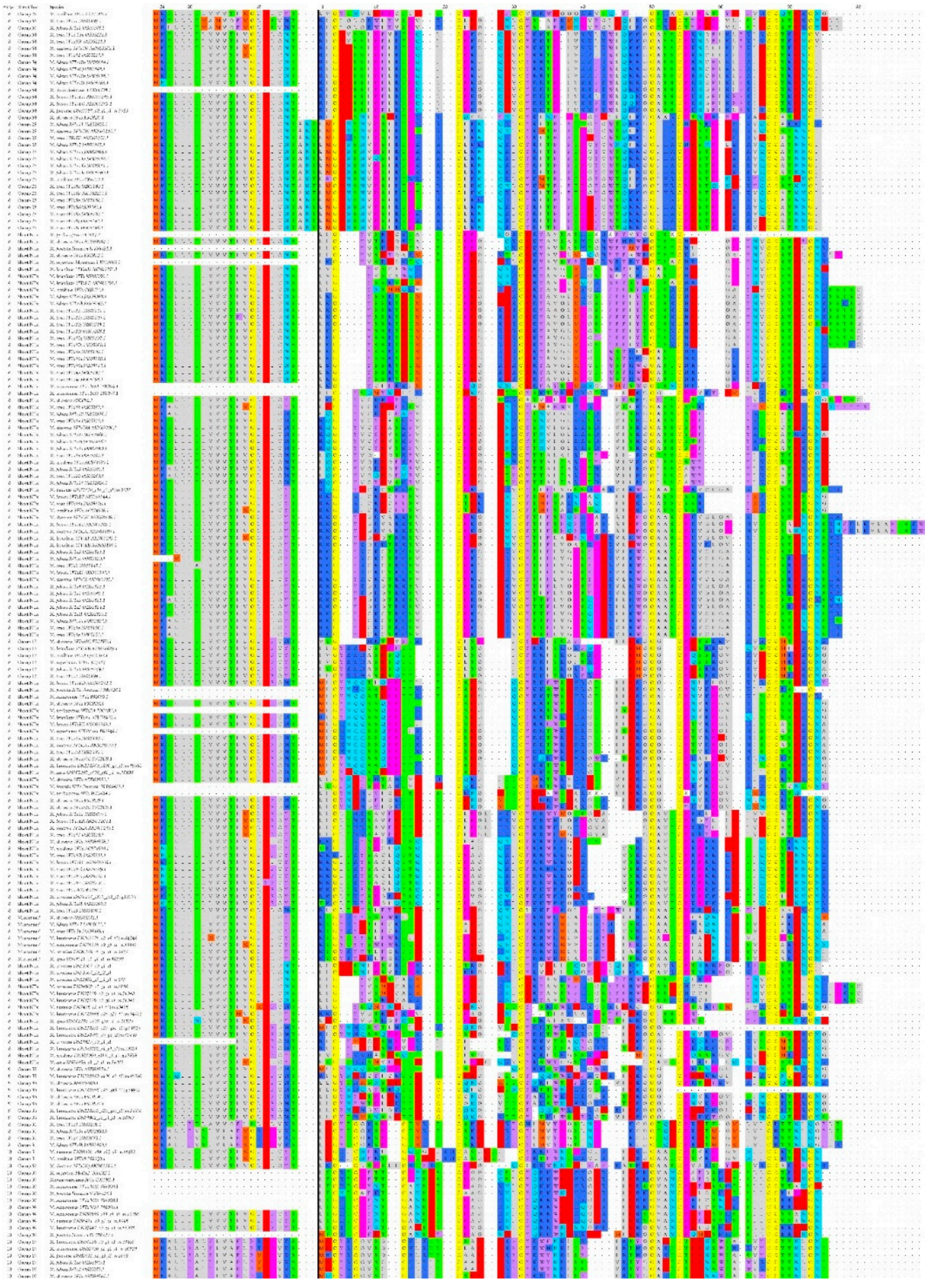

Figure S2. *Micrurus* venoms are rich in 3FTxs displaying an astonishing variety of primary structures. Micrurine 3FTxs may possess 8, 9, or 10 Cys residues with different disulfide patterns in each group. Pharmacologies are almost entirely unknown. Some probably target nicotinic acetylcholine receptors of reptilian neuromuscular junctions, but their potential targets in mammals are unknown. Most 3FTx have 21-residue signal peptides, shown to the left of the black vertical line; however, Group 28 is unique in that it has 24-residue signal peptides.

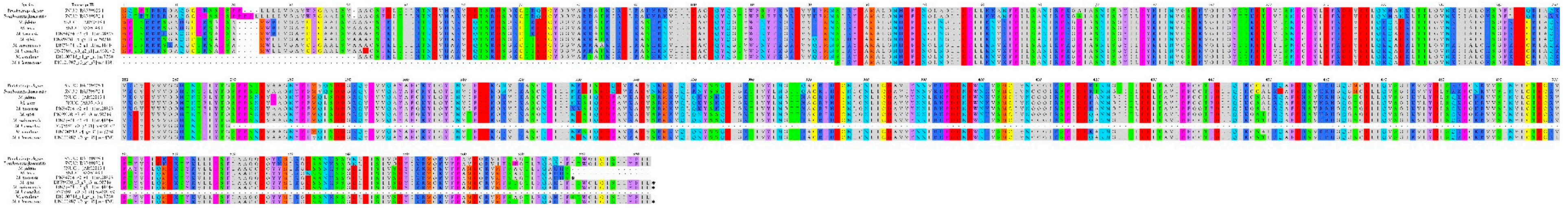

Figure S3. 5′-Nucleotidases dephosphorylate purine mononucleotides so as to release free nucleosides (principally adenosine), which contribute to prey hypotension [46]. All eight *Micrurus* species examined to date have 5′-nucleotidases in their venoms. These are present at levels ranging from ~0-0.05% (Table S2). With 573-588 residues and no signal peptides, micrurine venom 5NUCs are slightly shorter than their crotalid counterparts. They bear two different C-termini, one 14 residues longer than the other. Interestingly, the longer variant also appears in 5NUCs of the pitvipers, *Protobothrops elegans* and *Protobothrops flavoviridis* [11], and *Gloydius brevicaudus [275]*. Micrurine 5NUCS are structurally almost invariant, in addition to their close identity with the crotalid enzymes.

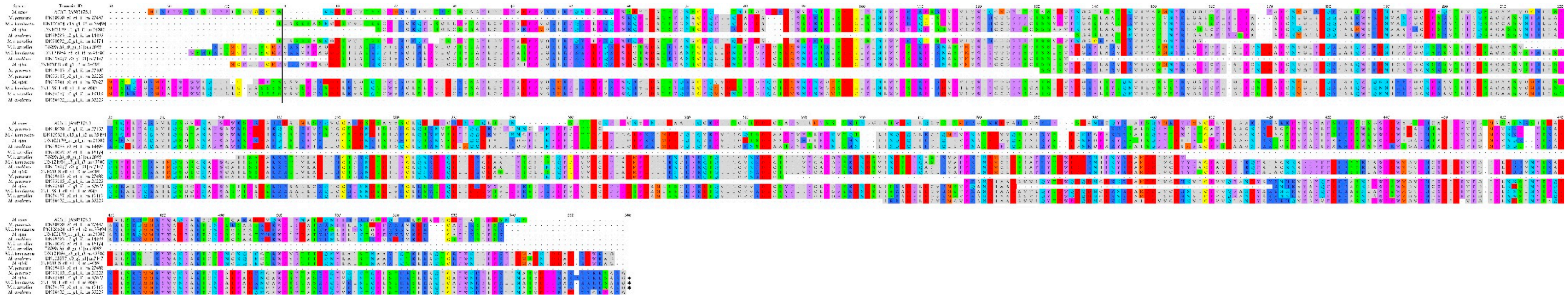

Figure S4. Micrurine acetylcholinesterases (AChEs) are modestly large proteins, containing approximately 585 amino acids. However, they are not expressed at biologically significant levels in *Micrurus* venoms, as they are in some Old World elapid taxa. The primary function of AChE in the venoms of those elapids that produce it, is presumably to starve neuromuscular junctions of transmitter, in concert with postsynaptic antagonists of nicotinic AChRs. Among the *Micrurus* transcriptomes, AChE is most abundant in *M. l. lemniscatus* venom, where AChE transcripts total only 0.05% of the transcriptome (Table S2). Micrurine AChEs are highly variable in primary structure. Some, such as *M. s. spixii* transcript DN97541_c0_g1_i1|m.57626, are predicted to have 29-residue signal peptides, while others, such as *M. tener* AChE (JAS05178.1), are expected to have signal peptides of only 23 residues, with shorter N-termini.

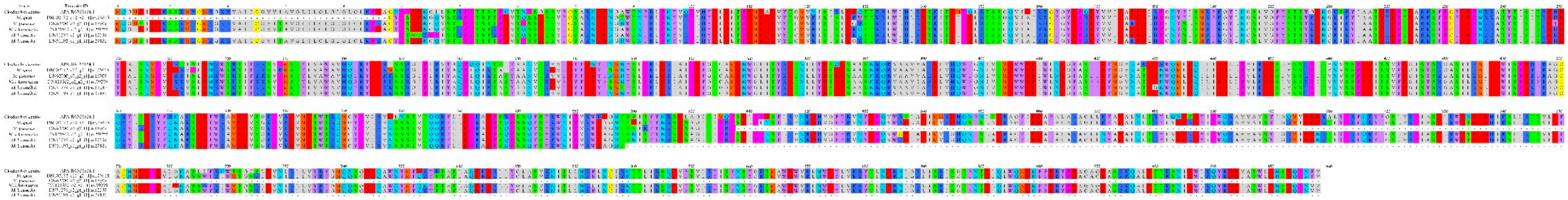

Figure S5. Aminopeptidase A (APA) converts kallidin to bradykinin [82,276], a potent inducer of hypotension. All of the transcriptomes except that of *M. surinamensis* had an APA sequence. The *Micrurus* sequences comprise 958 residues and are largely identical to the APA from *Gloydius brevicaudus* [277]. *Micrurus s. spixii* had the highest APA transcript abundance, but that constituted only 0.09% of the transcriptome (Table S2).

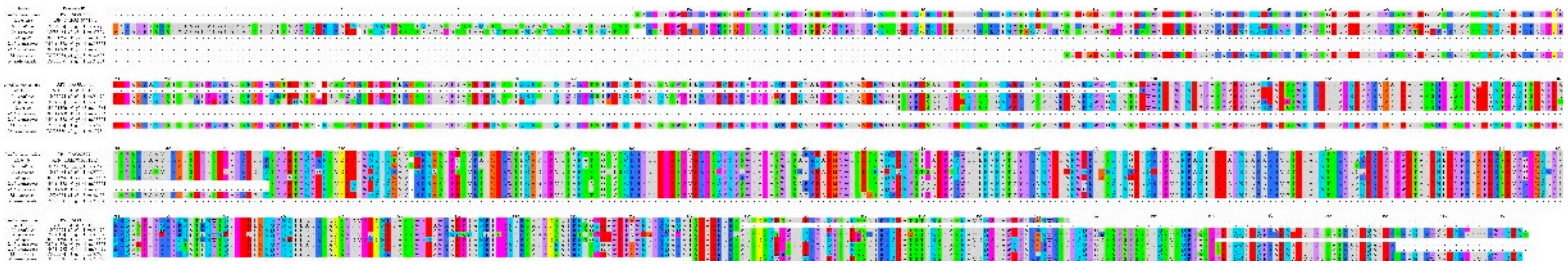

Figure S6. Like APA, Aminopeptidase N (APN) also converts kallidin to bradykinin [82,276]. All of our transcriptomes except for that of *M. paraensis* contained an APN transcript, as did that of *M. fulvius*. All were also incomplete, but were nearly identical to the *M. fulvius* sequence insofar as comparisons could be made. *Micrurus* sequences were also very similar to the *Gloydius brevicaudus* sequence (BAG82599.1; Yuko Ogawa and Ryohei Yanoshita, 2007, unpublished). Among micrurine APNs, that of *M. surinamensis* was the most abundant at 0.01% of its transcriptome (Table S2).

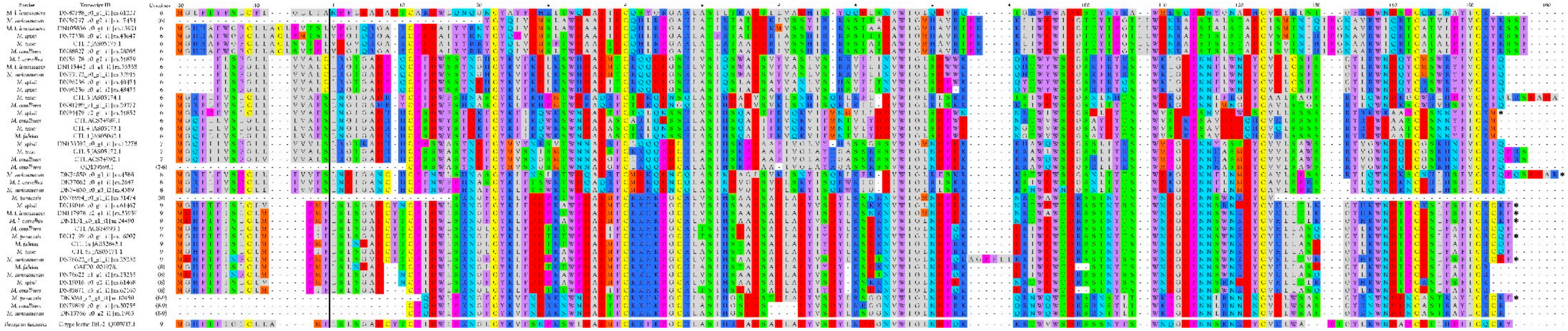

Figure S7. New World elapid C-type lectin-like proteins (CTLs) exhibit tremendous sequence variation, and may comprise as many as 8-10 structural subclasses. Micrurine CTLs may have 6, 7, 8, or 9 Cys residues, and the 7-Cys toxins present two different patterns, raising the possibility of heterodimeric CTLs. However, there have been no pharmacological characterizations of any *Micrurus* CTLs to date. Among our samples, CTLs ranged in abundance from trace quantities in *M. s. spixii* to 1.87% in *M. paraensis* and 3.35% in *M. corallinus*. These significant quantities suggest a role of some importance, at least in the latter two species. Signal peptide sequences are shown to the left of the heavy black vertical line.

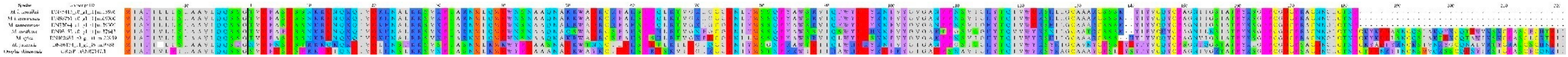

Figure S8. Various crotalid CRISPs act as L-type Ca^{2+} channel antagonists of depolarization-induced arterial smooth muscle contraction [278]; thus, they promote vasodilation and hypotension. All six *Micrurus* transcriptomes contained CRISP sequences of 221 residues plus 19-residue signal peptides (left of black line). The sequences from *M. l. carvalhoi*, *M. l. lemniscatus*, and *M. surinamensis* are identical over the first 201 residues, at which point they terminate. We do not know whether they are complete or truncated. The sequences of *M. corallinus* and *M. s. spixii* differ somewhat from the three foregoing, but that of *M. paraensis* is radically different, being more similar to the CRISP from *Ovophis okinavensis* venom [10]. Micrurine CRISPs range from trace levels in most species to 0.24% of the *M. l. lemniscatus* transcriptome (Table S2).

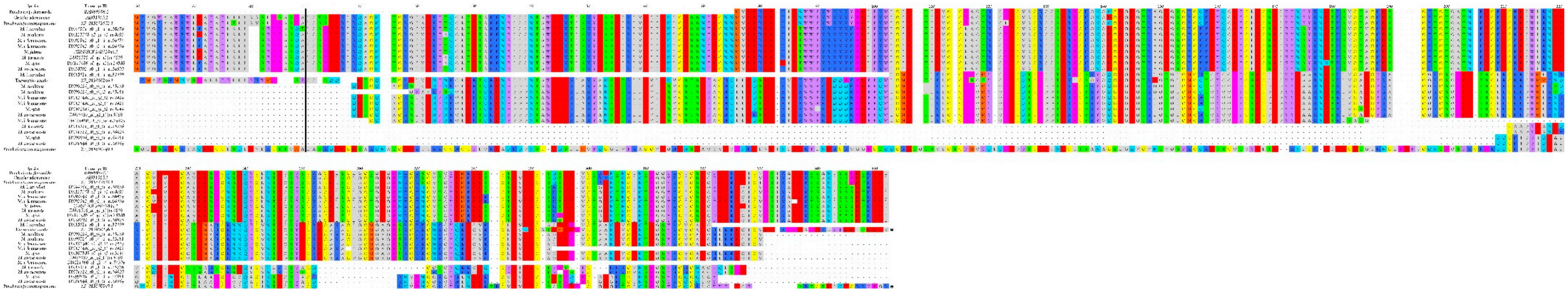

Figure S9. Micrurine CRISP-EGFs appear to comprise three protein subclasses, one of which includes CRISP-EGF transcripts from *Micrurus fulvius* and crotalid venoms and from colubrid tissues. This first subclass has 28-residue signal peptides. The second and third subclasses consist only of micrurine sequences from the present study. The second subclass is most closely related to an uncharacterized protein from the *Protobothrops mucrosquamatus* genome and a CRISP with an EGF domain from the *Thamnophis sirtalis* genome. Both of these proteins exceed 960 amino acids. The third subclass is represented only by two pairs of incomplete sequences, both of which appear related to fibulins. All four of these are expressed at trace levels (<0.0002%); thus, they may be contaminating tissue proteins.

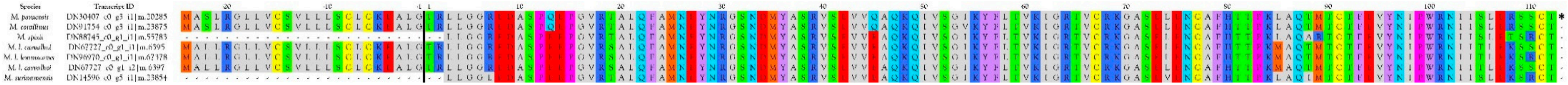

Figure S10. Cystatins have not been reported previously as snake venom constituents and we make no claim that they are. However, they are present in the *Micrurus* venoms examined here, although in only trace quantities (0-0.02% of the transcriptomes). Micrurine cystatins possess 24-residue signal peptides and have their Cys residues in the same positions as mammalian cystatins, with which they share only 45-55% of their amino acids. However, it appears that they probably are secreted proteins.

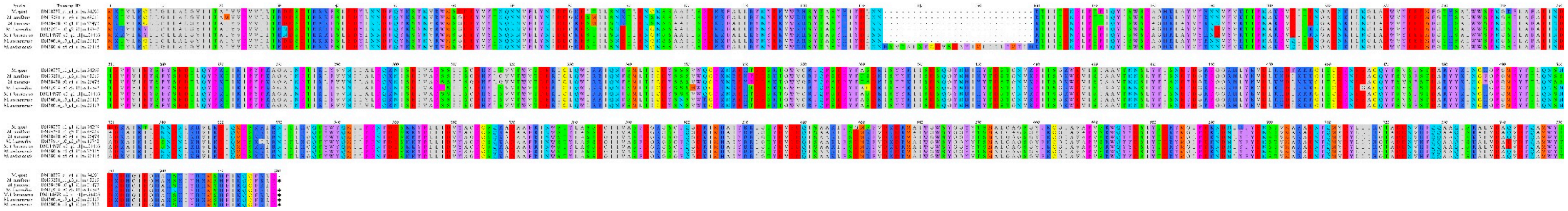

Figure S11. All six *Micrurus* transcriptomes contained a DPP IV transcript and that of *M. surinamensis* contained two. DPP IVs comprise 752-779 amino acids, but because they are membrane bound in microsomes [279], they have no signal peptides. One sequence from *M. surinamensis* has a 27-residue insert that is lacking in the other homologs.

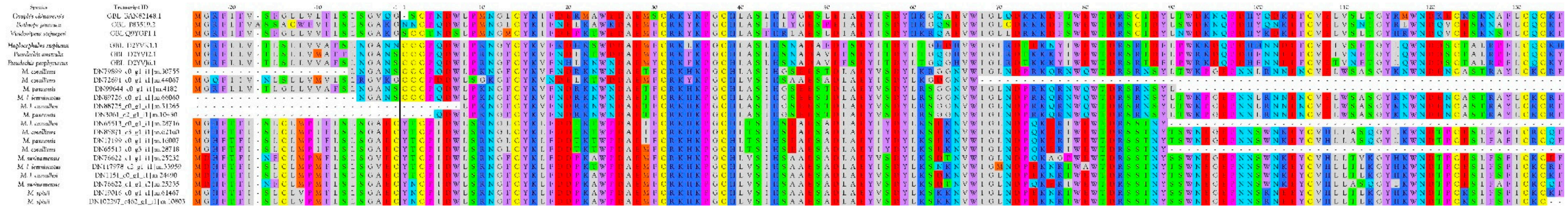

Figure S12. Aird et al. [10] postulated that the primary function of galactose-binding lectins (GBLs) may be to upregulate venom synthesis in the venom gland when venom supplies become depleted. However, with the present findings, that view may have to be revised somewhat. While several species in the present study also have typically low GBL titers (0-0.19%)(Table S2), *M. paraensis* (1.93%) and *M. corallinus* (3.35%) have the highest levels reported in any snake venom to date, suggesting that in these two species, GBL hemagglutinating and edematogenic properties [280] may play a significant role in envenomation. Signal peptides are shown to the left of the vertical black line.

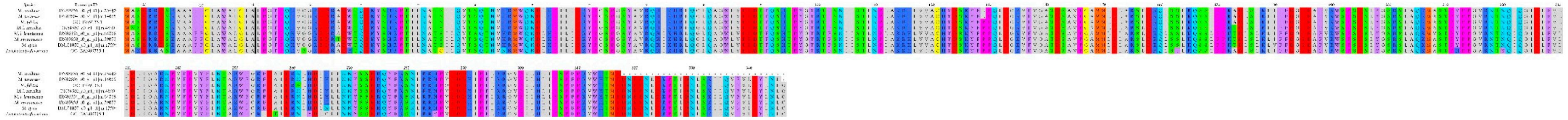

Figure S13. Guanylyl cyclase (GC) is believed to serve only the function of cyclizing N-terminal glutamine residues of specific venom proteins to prevent their rapid degradation in the host. For this reason, GC is not a snake venom constituent in the usual sense, although it is usually present in trace quantities. All six coral snake transcriptomes possessed identical 368-residue transcripts with a putative 23-residue signal peptide (left of black vertical line) that comprised from 0-0.02% of their respective transcriptomes. Even the GC transcript of the habu, *Protobothrops flavoviridis*, differed from the micrurine GCs at only 5 residues.

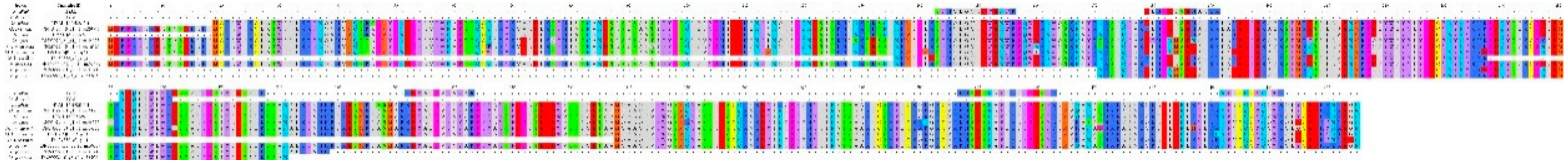

Figure S14. Hyaluronidases are generally minor enzymatic components of snake venoms. They have been recorded to date in 10 *Micrurus* venoms, including all six species that we studied. Micrurine hyaluronidases comprise 465 amino acids and lack signal peptides. In our transcriptomes, hyaluronidases accounted for between ~0% (*M. s. spixii* and *M. l. lemniscatus*) and 0.27% (*M. surinamensis*) of the transcriptomes. Consistent with the finding of Aird et al. (2015) that the most abundant venom proteins evolve most rapidly, hyaluronidases show strikingly little sequence variation. Most *Micrurus* species apparently have only one hyaluronidase transcript, but oddly, *M. l. carvalhoi* had three.

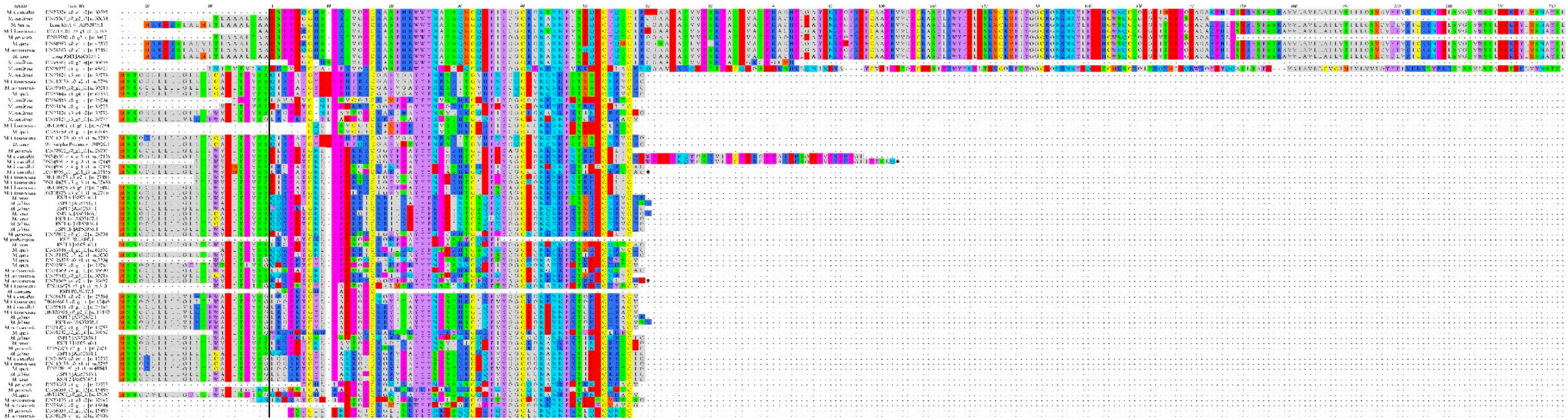

Figure S15. In addition to typical elapid Kunitz serine protease inhibitors (short KSPIs, 59-61 residues), some *Micrurus* venoms contain novel KSPIs (long KSPIs) 232 residues in length. The physiological targets of both classes are unknown. The long KSPIs should not be confused with SERPINs, which are nearly twice again as long (Figure S26). Signal peptides of either 20 or 24 residues are shown to the left of the black vertical line.

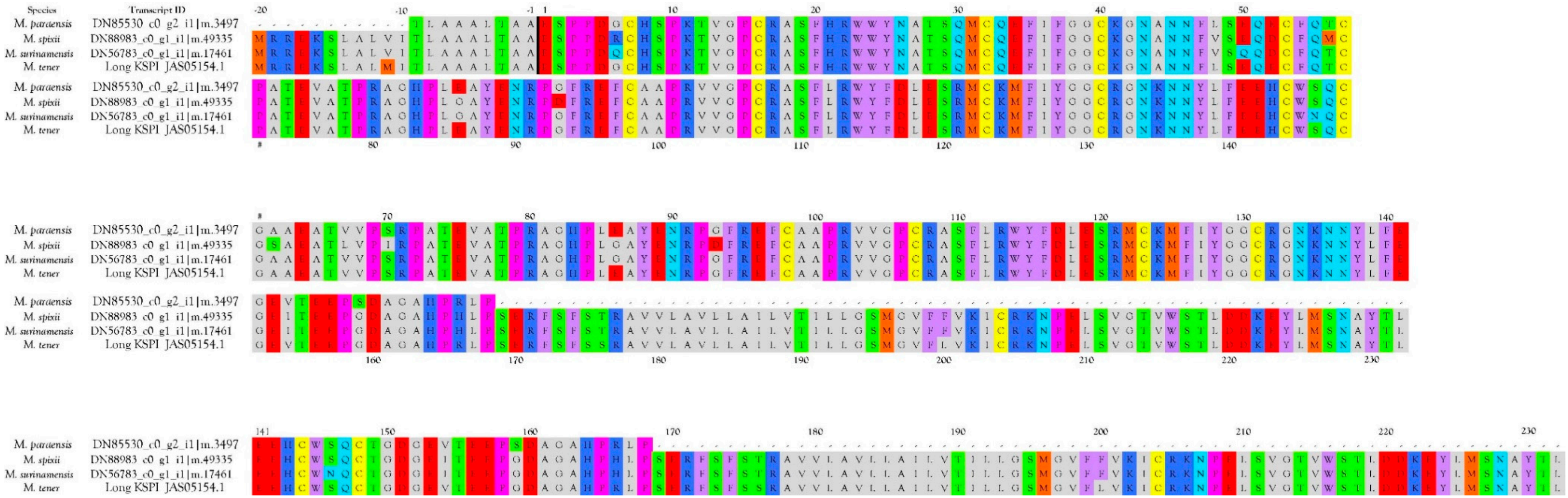

Figure S16. Four *Micrurus* species possess long KSPIs (232 residues) that appear to have arisen through gene duplication of a short KSPI, with the duplicated portion being grafted onto the C-terminus of a short KSPI. The long KSPIs have 13 Cys residues, but the disulfide pattern is unknown; however, the second group of six cysteines has the same spacing as the first six (See also Figure S15). In this figure, the C-terminal half of the sequences has been cut and re-aligned with the N-terminal half to illustrate the Cys pattern match.

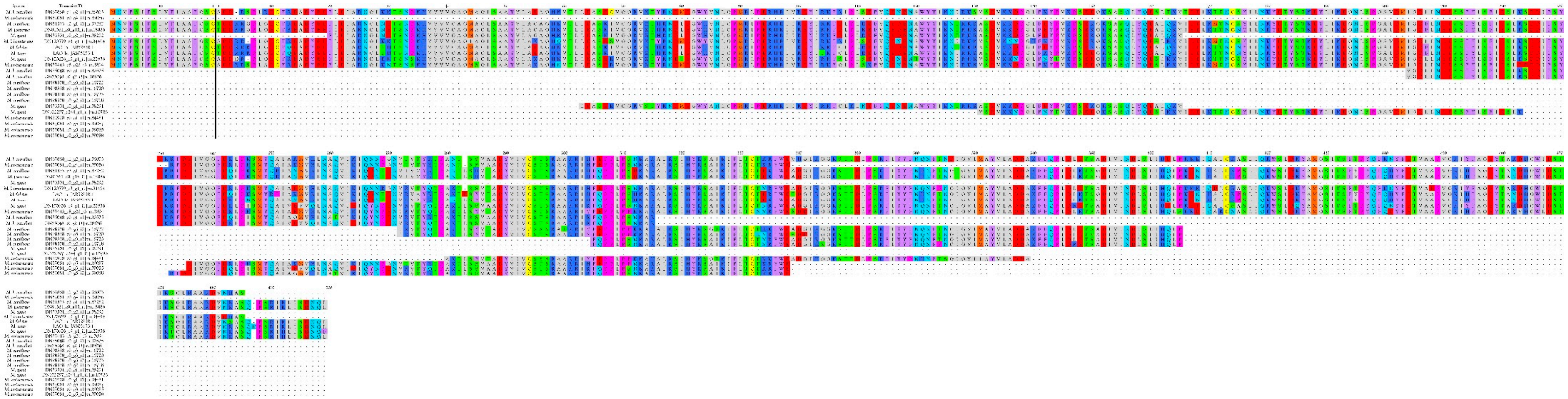

Figure S17. The flavin-enzyme, L-amino acid oxidase (LAO), is responsible for the bright yellow color of many solenoglyph venoms, in which LAO content can approach 10% [10]. Snake venom L-amino acid oxidase (EC 1.4.3.2) generates H_2O_2 from the conversion of L-amino acids to keto acids. It is a more minor constituent of *Micrurus* venoms, ranging from essentially 0% (*M. s. spixii*) to 0.84% (*M. corallinus*). Micrurine LAOs exhibit relatively little sequence variation, except at specific residues, where the same types of amino acid substitutions are encountered across species.

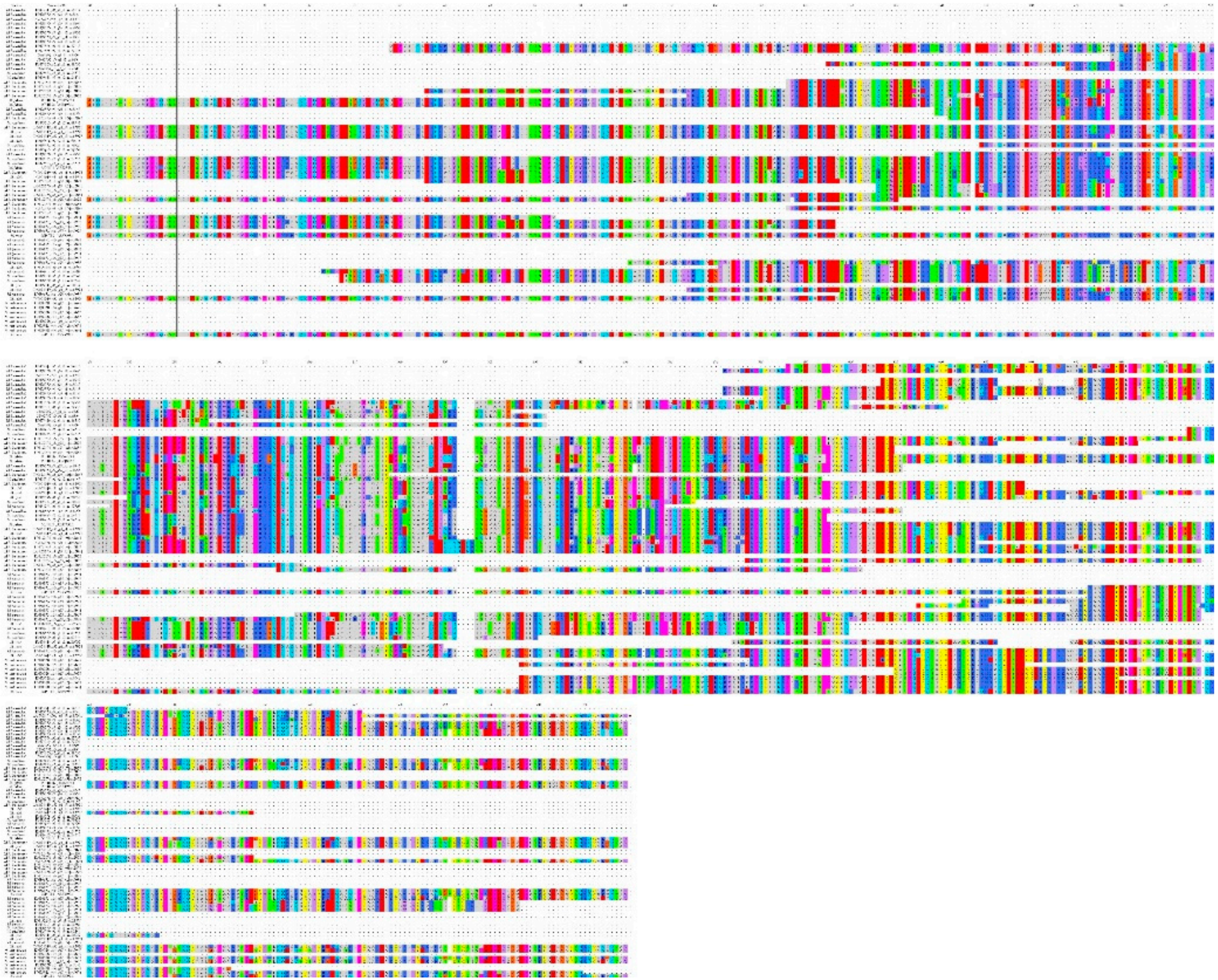

Figure S18. Most *Micrurus* venoms appear to contain only one Type III metalloprotease, comprising 614 residues, including a 20-residue signal peptide (left of the vertical black line). One *M. l. lemniscatus* transcript (lemniscatus DN101949_c32_g1_i4|m.13925) yields a mature protein with a mass of 66,657 D and a predicted pI of 8.37. This protein is most similar to atragin, from the venom of *Naja atra* [281]. The metalloprotease content of the venoms we examined varies from essentially 0% in *M. s. spixii* to 2.1% in *M. corallinus*. The latter suggests a significant function in envenomation. Nonetheless, the pharmacological and physiological effects provoked by micrurine metalloproteases are unknown.

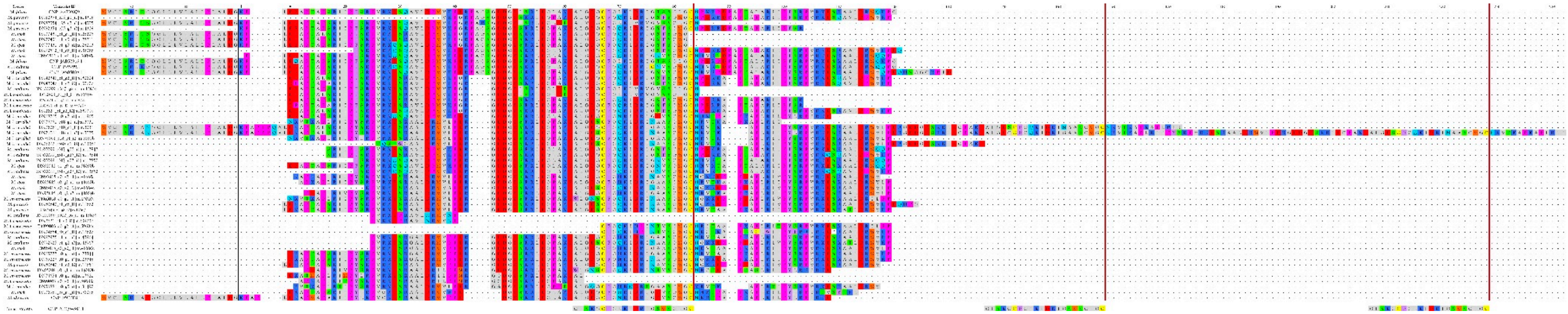

Figure S19. Natriuretic peptide transcripts were found in all six of the *Micrurus* species we investigated. They have also been reported in venom gland transcriptomes of *M. fulvius* and *M. altirostris* as well. Interestingly, two of our *M. l. carvalhoi* transcripts show evidence of gene duplication. One of them contains at least three copies of an NP. The sequence of human CNP is shown across the bottom for comparison. NP transcripts have 25-residue signal peptides (left of the vertical black line), but we cannot draw any conclusions regarding the total length of the transcripts. Solid brown lines separate the repeats.

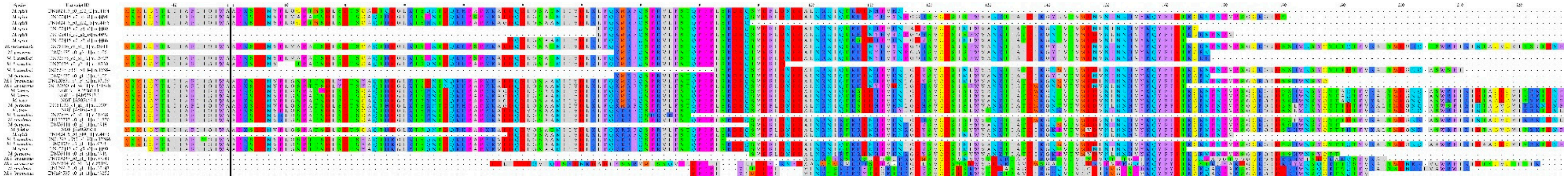

Figure S20. Micrurine nerve growth factor (NGF) transcripts encode 227 amino acids plus 18-residue signal peptides. They are highly similar sequences, displaying numerous blocks of invariant residues. Most variable positions show specific sets of substitutions, synonymous and non-synonymous, that occur without regard to species. The only strongly divergent sequences include a set of six transcripts from *M. l. lemniscatus*, which vary at numerous positions. Another group of four transcripts from *M. l. lemniscatus* and *M. corallinus* are also significantly different from all others; however, the significance of these variations, if any, is unknown.

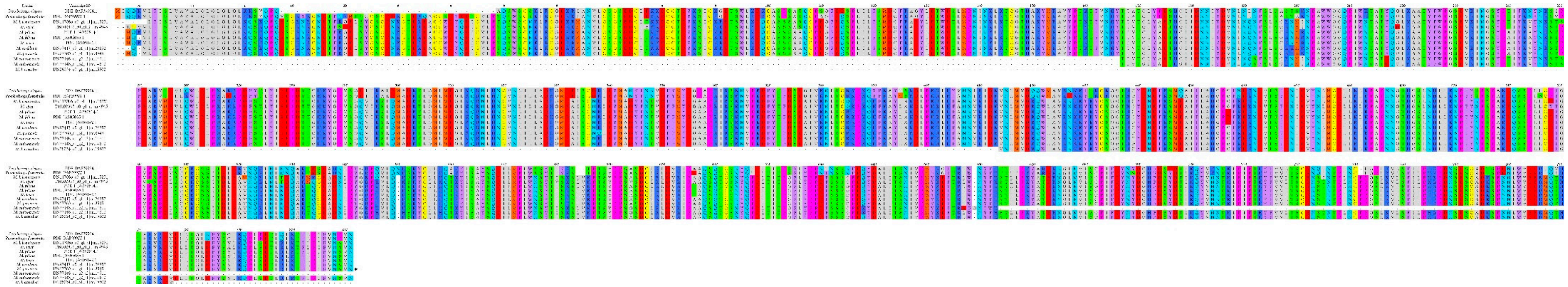

Figure S21. All of the *Micrurus* venoms investigated here apparently have a single phosphodiesterase gene, except for *M. surinamensis*, which barring a transcript misassembly, may have two. The transcript in question shows an inserted glycine at position 700, and a glutamate residue at position 698, where all other sequences have leucine. Signal P predicts signal peptides of 18 residues, but they are probably actually 22 residues long, given the unbroken 14-residue run of aliphatic amino acids. PDE sequences are highly conserved, given the similarity of *Micrurus* sequences to *Protobothrops* sequences (Old World crotalids). PDEs are expressed at trace levels in five of the *Micrurus* species and at 0.02% in *M. corallinus*

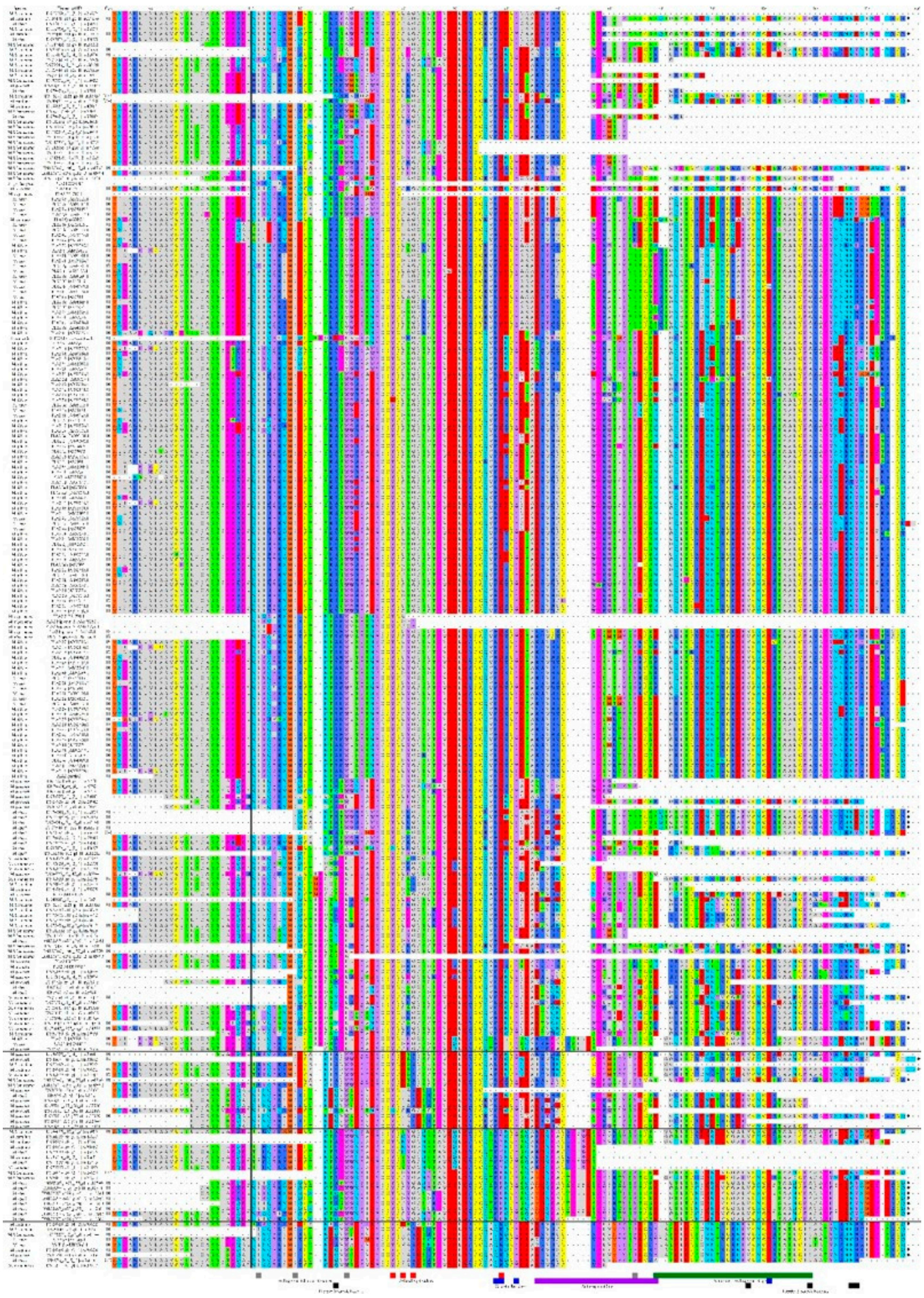

Figure S22. Structures of micrurine phospholipases A_2, illustrating their great structural diversity. Of 244 PLA_2s with partial or complete structures, 202 are apparently catalytic, having the requisite H48, D49, Y52, and D101 in their active sites. The remaining 42 are apparently non-catalytic. Positions indicated in red across the bottom designate residues involved in Ca^{2+}-binding [187]. Positions indicated in blue are catalytic residues. The anticoagulant site [188] and the neurotoxic hydrophobic region [189,190] are indicated by purple and green bars, respectively. Residues proposed by Alape-Girón et al. [187] to be involved in myotoxicity of elapid PLA_2s are indicated below with black cells. Residues participating in the hydrophobic channel, via which substrate enters the catalytic site are indicated in gray [191]. PLA_2s from the two North American species, *M. fulvius* and *M. tener*, form a structural cluster that is relatively distinctive from the much more variable South American sequences, which probably also represent much greater pharmacological diversity. Signal predicted signal peptides of 21 residues; however, elapid PLA_2s sequenced by Edman degradation commence with residue 28, confirming that the signal peptides are actually 27 residues in length. Numbers of

cysteine residues are given for sequences that are complete. Numbers of cysteines in parentheses are for sequences truncated at the N-terminal end, for which the cysteines can be reliably predicted, owing to the relative invariability of the N-termini. Asterisks indicate stop codons

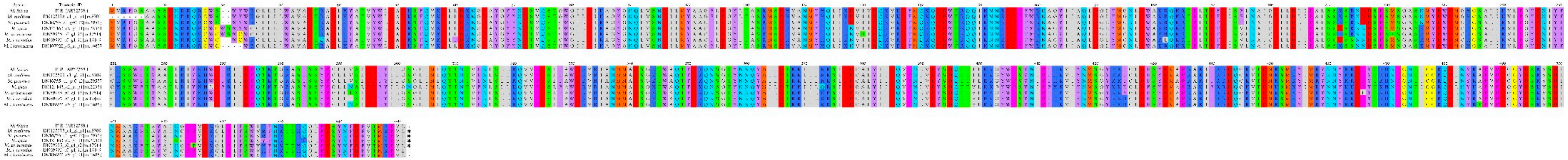

Figure S23. A phospholipase B (PLB) transcript was found in each of the six *Micrurus* species examined. PLB is believed to contribute to envenomation by hydrolyzing membrane phospholipids [282].

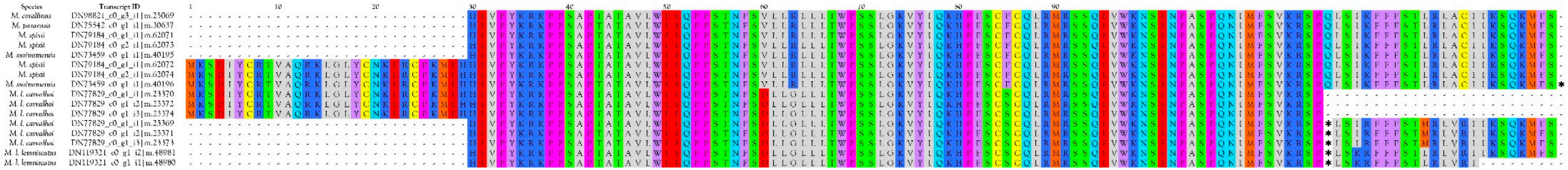

Figure S24. RNAse is essentially absent in all of the transcriptomes, except for that of *M. surinamensis*, where it comprises 0.7% of the transcriptome. Micrurine RNAse transcripts have 141 residues and apparently lack signal peptides. The sequences are largely invariant, showing amino acid substitutions, all non-synonymous, at only 3 positions (V/D60; R/G63; F/S85). Oddly, 3 *M. l. carvalhoi* transcripts and both transcripts from *M. l. lemniscatus* have stop codons at position 118. The functional significance of this truncation is unknown. The loss of the C-terminal 24 residues suggests that that these have become pseudogenes, since the next 17 residues after the stop codon are the same as in full-length RNAse transcripts. However, we cannot exclude the possibility that these apparently dysfunctional enzymes have adopted another function instead.

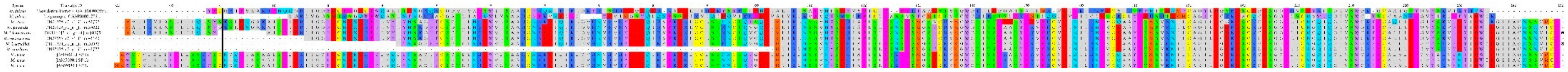

Figure S25. A single serine protease transcript of 265 residues was found in each of the six *Micrurus* venoms examined in the present study. The signal peptide accounts for the first 18 residues. Except for their signal peptides, these protease sequences align well with the transcripts published from *M. tener* venom. Two serine protease transcripts from the transcriptome of *M. fulvius* do not appear closely related to the other seven. Given the great similarity of the micrurine serine proteases presented here, the *M. fulvius* sequences may represent contaminating blood or tissue serine proteases. The functions of micrurine serine proteases are unknown.

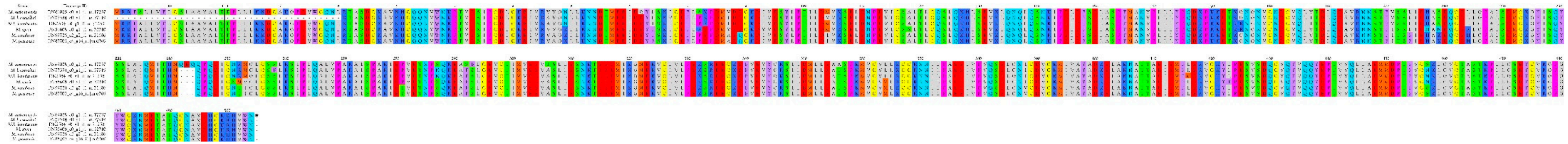

Figure S26. Serpins consist of 501-504 residues plus a 19-residue signal peptide (left of vertical black line). They are approximately 6x longer than KSPIs (Figure S15). In *Micrurus* venom glands, these transcripts are almost invariant, showing amino acid substitutions at only about 24 positions. Serpins constitute 0-0.04% of the transcriptomes examined in this study. Their probable role in envenomation is to contribute to anticoagulation.

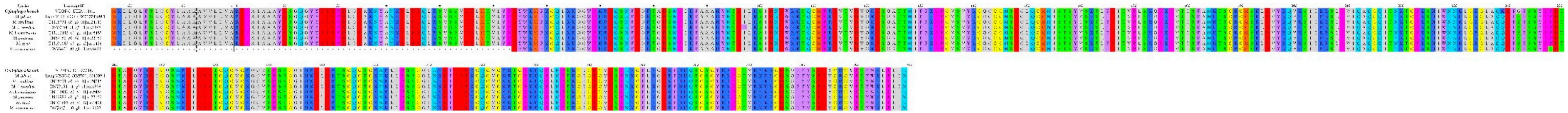

Figure S27. Micrurine long VEGFs (VEGF-Cs) are nearly structurally invariant. VEGF-C mRNA transcripts comprise 399 residues, with a signal peptide of uncertain length. SignalP v4.1 predicts 16-residue signal peptides (dashed vertical line), but this would leave a long, hydrophobic N-terminus. While that is possible, a 24-residue signal peptide would seem more reasonable (solid vertical black line). No VEGF-C has been reported from *Micrurus tener* venom, which does have a short VEGF.

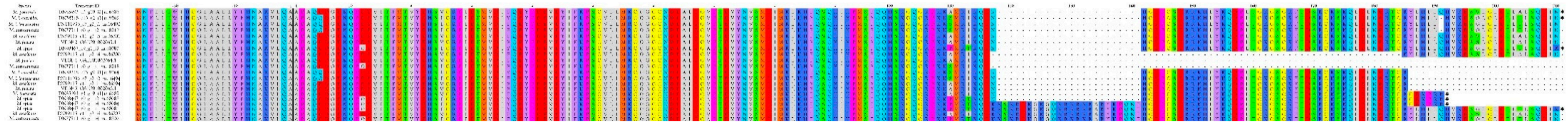

Figure S28. Short VEGFs (VEGF-Fs) are conservative in amino acid composition, but display at least three major structural variants. Position 142 may be N or K, or missing altogether in one transcript from *M. corallinus*. Toxins with K142 then have an extremely basic 24-residue insert, with 14 K or R residues from positions 142-162. The majority of VEGF-Fs have a hydrophilic 25-residue C-terminal sequence, commencing with a short hydrophobic segment, YLHLL. However, three toxins terminate with a short hydrophilic sequence, CEKPRR. It is unclear whether sequences 9-13 are truncated or complete sequences. VEGF-Fs were present in all six of our transcriptomes. They have 26-residue signal peptides (vertical black line).

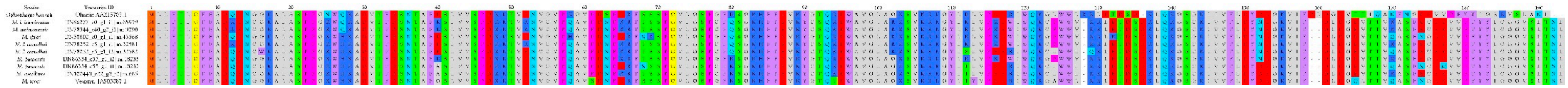

Figure S29. Ohanin, the first known vespryn, which was isolated from the venom of the king cobra (*Ophiophagus hannah*), produces hypolocomotion and hyperalgesia in mice [244]. Its effects in snakes may be similar, but at present, no pharmacological data are available. All six Brazilian *Micrurus* venoms possessed single vespryn sequences, except for the venoms of *M. paraensis* and *M. l. carvalhoi*, in which there were two. *Micrurus* vespryns are nearly identical to ohanin and display limited sequence variation. Micrurine vespryn transcripts comprise 190 residues and lack signal peptides. Vespryns appear at trace levels in most of the venoms examined here, although they comprised 0.22% of the *M. s. spixii* transcriptome and 1.66% in *M. corallinus*.

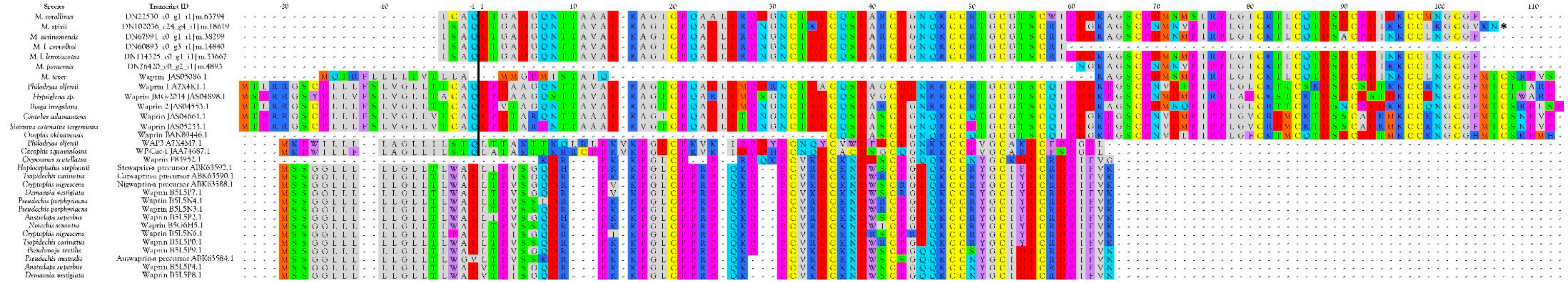

Figure S30. Micrurine waprin-like sequences are more similar to opisthoglyph colubrid and crotalid waprin-like sequences than to those of Old World elapids. Waprin-like sequences have been reported from all *Micrurus* venoms except that of *M. fulvius*. Colubrid and crotalid waprin-like proteins have 23-residue signal peptides (left of vertical black line). None of our sequences have complete signal peptides and the sequence from *M. tener* is so different at the N-terminus that no definite conclusions can be drawn regarding it length in *Micrurus*. However, it appears that all micrurine waprin-like sequences have an N-terminal Q, as in the colubrid and crotalid sequences.
