# Supplementary material for: Addendum: Aird, S.D. et al. Coralsnake Venomics: Analyses of Venom Gland Transcriptomes and Proteomes of Six Brazilian Taxa. Toxins 2017, 9(6), 187
Source: Toxins (Basel). 2018 Apr 24;10(5):172. doi: 10.3390/toxins10050172 (PMC5982091; doi:10.3390/toxins10050172)
Supplement: Supplementary file 1 [file toxins-10-00172-s001.zip › supplementary/Figure S2.pdf]

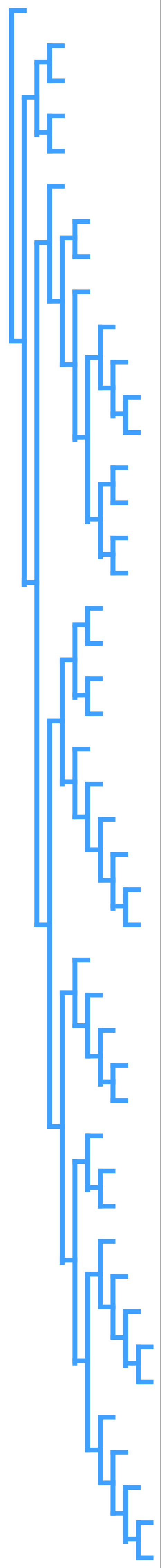

	Taxon	ID #	Cys	1 10 20 30 40 50 60	
	B. multicinctus	CAD01082.1	10	MQCKTCSFYTCPNSETCPDGKNICVKRSWTAVRGDGPKREIRRECAATCPPSKLGLTVFCCTTDNCNH	
1	*surinamensis*	IACN01019456.1	9	ILCYNHQAYTRQTTECCQIGETNCYRTTWNDHR----GTRIERGCG--CHDVKRGIKIKCCSYDKCNG	*
2	*corallinus*	IACJ01051157.1	9	ILCYNHQAYTRQTTECCQIGETNCYRTTWNDHR----GTRIERGCG--CYEVKPGVQMKCCKTDKCND	*
3	*lemniscatus*	IACK01168435.1	9	ILCYNHQAYTRQTTECCQIGETNCYRTTWNDHR----GTRIERGCG--CPYVKPGVKINCCTTDKCNA	*
4	*spixii*	IACM01128650.1	9	ILCYNHQAYTRQTTECCQIGETNCYRTTWNDHR----GTRIERGCG--CPYVKPGVKINCCTTDKCNA	*
5	*paraensis*	IACL01074030.1	9	LLCYTHMSNSAETTECCQNGKTTCYTKIWTDVR----GTVIERGCG--CPDKKYGGKINCCATDKCNS	*
6	*lemniscatus*	DN94862_c6_g3_i1|m.60964	9	LLCYTHMSNSAETTECCQNGKTTCYTKIWSDFR----GTVIERGCG--CPDKKYGGKINCCATDKCNS	*
7	*lemniscatus*	IACK01171799.1	9	LLCYTHMSNSAETTECCQNGKTTCYTKIWSDFR----GTVIERGCG--CPDKKYGGKINCCATDKCNS	*
8	*surinamensis*	IACN01118586.1	9	LLCYTHMSNSAETTECCQNGKTTCYTKIWSDFR----GTVIERGCG--CPDKKYGGKINCCKTDRCNS	*
9	*spixii*	IACM01029744.1	9	LLCYTHMSNSDETTECCQNGMTTCYNKIWSDFR----GTVIERGCG--CPKTEYRVKITCCQTDKCNR	*
10	*paraensis*	IACL01074031.1	9	LLCYTHMSNSDETTECCQNGMTTCYNKIWSDFR----GTVIERGCG--CPKTEYRVKITCCQTDKCNR	*
11	*surinamensis*	IACN01118591.1	9	LLCYTHMSNSAETTECCQNGMTTCYNKIWSDFR----GTVIERGCG--CPKTEYRVKITCCQTDKCNR	*
12	*carvalhoi*	IACI01013364.1	9	LLCYTHMSNSDETTECCQNGMTTCYNKIWSDFR----GTVIERGCG--CPKTEYRVKITCCQTDKCNR	*
13	*carvalhoi*	IACI01013363.1	9	LLCYTHMSNSDETTECCQNGKTTCYTKIWSDFR----GTVIERGCG--CPDKKYGGKINCCQTDKCNS	*
14	*lemniscatus*	DN94862_c6_g1_i1|m.60963	9	LLCYTHMSNSAETTECCQNGKTTCYTKIWSDFR----GTVIERGCG--CPDKKYGGKINCCQTDKCNS	*
15	*lemniscatus*	IACK01171797.1	9	LLCYTHMSNSAETTECCQNGKTTCYTKIWSDFR----GTVIERGCG--CPDKKYGGKINCCQTDKCNS	*
16	*paraensis*	IACL01074029.1	9	LLCYTHMSNSAETTECCQNGKTTCYTKIWSDFR----GTVIERGCG--CPDKKYGGKINCCQTDKCNS	*
17	*paraensis*	IACL01031944.1	9	-LCYNHMSSRPETTECCENGETTCYNKSWNS------GRIIERGCG--CPNVKPGIKLNCCQTDRCNG	*
18	*paraensis*	IACL01031940.1	9	-LCYNHMSSRPETTECCENGETTCYNKSWNS------GRIIERGCG--CPNVKPGIKLNCCQTDRCNG	*
19	*paraensis*	IACL01031946.1	9	-LCYNHMSSRPETTECCENGETTCYNKSWNS------GRIIERGCG--CPNVKPGIKLNCCQTDRCNS	*
20	*surinamensis*	IACN01111607.1	9	LLCYNHMSSRPETTECCENGETTCYNKSWNS------GRIIERGCG--CPNVKPGIKLNCCQTDRCNS	*
21	*surinamensis*	IACN01070775.1	9	LLCYNHMSSRPETTECCENGMTTCYEKSWRETRSSFSGTIIDRGCG--CPNVKPGTKLNCCKTDRCNG	*
22	*corallinus*	IACJ01138868.1	9	LICYNDHGYTGKTTECCENGMTTCYEKSWRETRSSFSGTIIDRGCG--CPNVKPGIKLNCCQTDRCNS	*
23	*spixii*	IACM01029760.1	9	LICYNDHGYTGKTTECCENGMTTCYEKSWRETRSSFSGTIIDRGCG--CPNVKPGTKLNCCKTDRCNG	*
24	*paraensis*	DN109051_c0_g1_i1|m.191	9	LICYNDHGYTGKTTECCENGMTTCYEKSWRETRSSFSGTIIDRGCG--CPNVKPGTKLNCCKTDRCNG	*
25	*corallinus*	IACJ01138867.1	9	LICYNDHGYTGKTTECCENGMTTCYEKSWRETRSSFSGTIIDRGCG--CPNVKPGTKLNCCKTDRCNG	*
26	*lemniscatus*	DN109162_c2_g1_i3|m.11006	9	LICYNDHGYTGKTTECCENGMTTCYEKSWRETRSSFSGTIIDRGCG--CPNVKPGTKLNCCKTDRCNG	*
27	*lemniscatus*	IACK01096299.1	9	LLCYTHMSNSAETTECCQNGLTTCFQQSWRDIR----GDRIERGCG--CPDEKKGIELKCCKTDRCNG	*
28	*corallinus*	IACJ01062274.1	9	LLCFNDHGYIGKTTECCQNGLTTCFQQSWRDIR----GDRIERGCG--CPKVKPGIQINCCTTEKCNG	*
29	*lemniscatus*	DN120340_c190_g2_i7|m.46960	9	RICYNDHGYIGKTTETCEDGITICYQQSWRDFR----GDRIERGCG--CPDEKKGIELKCCNCDKCNG	*
30	*corallinus*	IACJ01062275.1	9	LLCFNDHGYIGKTTECCQNGLTTCFQQSWRDIR----GDRIERGCG--CPDEKKGIELKCCKTDRCNG	*
31	*lemniscatus*	IACK01096298.1	9	LLCFNDHGYIGKTTECCQNGLTTCFQQSWRDIR----GDRIERGCG--CPDEKKGIELKCCKTDRCNG	*
32	*spixii*	IACM01029696.1	9	LICYNYWTPLDKTTESCGNGVTTCFAKSWRDHI----GLRTDRGCG--CPKTNPGIKLNCCSCNRCNG	*
33	*paraensis*	IACL01031941.1	9	MICYNQQSSQPPTTTTCSEG--QCYKKSWRDLH----GWRTDRGCG--CPKTNPGIKLNCCSCNRCNG	*
34	*surinamensis*	IACN01117435.1	9	MICYNQQSSQPPTTTTCSEG--QCYKKSWRDLH----GWRTDRGCG--CPKTNPGIKLNCCSCNRCNG	*
35	*surinamensis*	IACN01099285.1	9	IICYNDWTYHVKNAVSCENGITKCYKKFWFENR----GARYEKGCG--CPN-----NAKCCSCDKCNG	*
36	*corallinus*	IACJ01147547.1	9	IICYNDWTYHVKNAVSCENGITHCYKASSYGHR----GLNLKRGCG--CPD-----RAKCCLCDKCNV	*
37	*surinamensis*	IACN01099288.1	9	IICYNDWTYHVKNAVSCENGITHCYKASSYGHR----GLNLKRGCG--CPD-----RAKCCLCDKCNV	*
38	*paraensis*	IACL01042493.1	9	IICYNDWTYHVKNAVSCENGITHCYKASSYGHR----GLNLKRGCG--CPD-----RAKCCLCDKCNV	*
39	*spixii*	IACM01083762.1	9	IICYNDWTYHVKNAVSCENGITHCYKASSYGHR----GLNLKRGCG--CPD-----RAKCCLCDKCNV	*
40	*surinamensis*	IACN01099286.1	9	MICYSETPFTHKTTVSCENGITKCYKKFWFENR----GARYEKGCG--CPN-----NAKCCSCDKCNG	*
41	*spixii*	IACM01086450.1	9	MICYSETPFTHKTTVSCENGITKCYKKFWFENR----GARYEKGCG--CPN-----NAKCCSCDKCNG	*
42	*paraensis*	IACL01081214.1	9	MICYSETPFTHKTTVSCENGITKCYKKFWFENR----GARYEKGCG--CPN-----NAKCCSCDKCNG	*
43	*corallinus*	IACJ01022054.1	9	MICYSETPFTHKTTVSCENGITKCYKKFWFENR----GARYEKGCG--CPN-----NAKCCSCDKCNG	*
44	*lemniscatus*	IACK01212631.1	9	MICYSETPFTHKTTVSCENGITKCYKKFWFENR----GARYEKGCG--CPN-----NAKCCSCDKCNG	*
