# Supplementary material for: Addendum: Aird, S.D. et al. Coralsnake Venomics: Analyses of Venom Gland Transcriptomes and Proteomes of Six Brazilian Taxa. Toxins 2017, 9(6), 187
Source: Toxins (Basel). 2018 Apr 24;10(5):172. doi: 10.3390/toxins10050172 (PMC5982091; doi:10.3390/toxins10050172)
Supplement: Supplementary file 1 [file toxins-10-00172-s001.zip › supplementary/Figure S3.pdf]

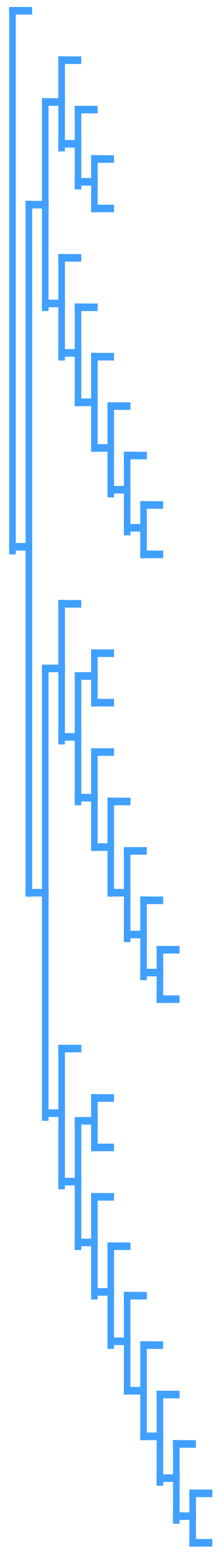

Seq.	Taxon	ID #	Cys	1 10 20 30 40 50 60 70
	B. multicinctus	CAD01082.1	10	MQCKTCSFYTCPNSET--CPDGKNICVKRSWTAVRGDGPKREIRRECAATCPP-SKLGLTVFCCTTDNCNH
1	*corallinus*	IACJ01056796.1	10	LTCKTCPFNTCADSET--CPVGKNICYQRKWNEHSG-----KKIERGCVANCPELGSHDTFLLCCRRDNCN*
2	*corallinus*	IACJ01056795.1	10	LTCKTCPFNTCADSET--CPVGKNICYQRKWNENNG-----KKIERGCVANCPELGSHDTSLLCCRRDDCN*
3	*carvalhoi*	IACI01112741.1	10	LTCKTCPFNTCADSET--CPVGKNICYQRKWNENNG-----KKIERRCVANCPELGSHDTSLLCCRRDDCN*
4	*lemniscatus*	DN97447_c0_g1_i1|m.63038	10	LTCKTCPFNTCADSET--CPVGKNICYQRKWNENNG-----KKIERGCVANCPELGSHDTSLLCCRRDDCN*
5	*paraensis*	DN86421_c0_g1_i1|m.1945	10	LTCHTCPYNTCANSET--CPAGKNICYQKKWEEHQG-----ERIERSCVANCPEFESSHSSLLCCTTANCN*
6	*lemniscatus*	DN169781_c2_g1_i1|m.39582	10	LTCHTCPYNTCANSET--CPAGKNICYQKKWEEHQG-----ERIERSCVANCPEFESSHSSLLCCTTANCN*
7	*carvalhoi*	IACI01044515.1	10	LTCHTCPYNTCANSET--CPAGKNICYQKKWEEHQG-----ERIERSCVANCPEFESSHSSLLCCTTANCN*
8	*carvalhoi*	IACI01115070.1	10	LTCHTCPYNTCANSET--CPAGKNICYQKKWEEHQG-----ERIERSCVANCPEFESSHSSLLCCTTANCN*
9	*carvalhoi*	IACI01064819.1	10	LTCHTCPYNTCANSET--CPAGKNICYKKKWEEHQG-----ERIERSCVANCPEFESSHSSLLCCTTANCN*
10	*corallinus*	IACJ01043429.1:	10	LTCHTCPYNTCANSET--CPAGKNICYQKKWEEHQG-----ERIERSCVANCPEFESSHSSLLCCTTANCN*
11	*surinamensis*	DN82939_c15_g1_i1|m.13986	10	LTCHTCPYNTCANSET--CPAGKNICYQKKWEEHQG-----ERIERSCVANCPEFESSHSSLLCCTTANCN*
12	*surinamensis*	IACN01100674.1	10	LECKICNFKICQTGELRHCASGETICYKTFWNTHRG-----LRIDRGCAATCPIAEN-HDSVECCAKDNCNN*
13	*corallinus*	IACJ01101103.1	10	LECKICNFKTCPTDELRRCASGETICYKTFWNTHRG-----LRIDRGCAATCPTVKPG-VNIICCKTDNCN*
14	*lemniscatus*	DN31338_c0_g4_i1|m.63664	10	LECKICNFKTCPTDELRRCASGETICYKTFWNTHRG-----LRIDRGCAATCPTVKPG-VNIICCKTDNCN*
15	*carvalhoi*	DN3636_c0_g1_i1|m.28697	10	LECKICNFKTCPTDELRRCASGETICYKKFWNTPHG-----LRIDRGCAATCPTVKPG-VNIICCKTDNCN*
16	*carvalhoi*	DN3636_c0_g2_i1|m.28698	10	LECKICNFKICQTGELRHCASGETICYKKFWNTPHG-----LRIDRGCAATCPTVKPG-VNIICCKTDNCN*
17	*paraensis*	IACL01053498.1	10	LECKICNFKICQTGELRHCASGETICYKKFWNTPHG-----LRIDRGCAATCPTVKPG-VNIICCKTDNCN*
18	*paraensis*	IACL01053506.1	10	LECKICNFKICQTGELRHCASGETICYKKFWNTPHG-----LRIDRGCAATCPTVKPG-VNIICCKTDNCN*
19	*lemniscatus*	DN31338_c0_g2_i1|m.63663	10	LECKICNFKICQTGELRHCASGETICYKKFWNTPHG-----LRIDRGCAATCPTVKPG-VNIICCKTDNCN*
20	*paraensis*	DN85120_c84_g37_i5|m.16452	10	LECKICNFKICQTGELRHCASGETICYKKFWNTPHG-----LRIDRGCAATCPTVKPG-VNIICCKTDNCN*
21	*lemniscatus*	DN60256_c0_g1_i1|m.69455	10	MECYRCGVSGCHLKTT--CSADEKFCY-KWFNKITR-----IRT-YGCAETCTEENSWTASVYCCTTNLCNT*
22	*paraensis*	DN85432_c2_g3_i1|m.2648	10	MECYRCGVSGCHLRIT--CSADEKFCY-KWFNKITR-----IRT-YGCAETCTEENSWTASVYCCTTNLCNT*
23	*spixii*	IACM01149051.1	10	MDCHRCGVSGCHLKTT--CSADEKFCY-KWFNKITR-----IRT-YGCAETCTEENSWTARVYCCTTNLCNT*
24	*spixii*	DN79827_c1_g8_i1|m.37076	10	MKCYKCGVSGCHLKIT--CSEDEKFCY-KWYNKISG-----IRT-HGCAKTCTEENSWTASVYCCTTNLCNT*
25	*paraensis*	IACL01013322.1	10	MECYRCGVSGCHLRIT--CSADEKFCY-KWYNKISG-----IRT-HGCAKTCTEENSWTASVYCCTTNLCNT*
26	*paraensis*	DN85432_c2_g3_i2|m.2649	10	MKCYRCGVSGCHLKTT--CAADEKFCY-KWYNKISF-----IRT-HGCAKTCTEENSWTASVYCCTTNLCNT*
27	*corallinus*	DN91848_c0_g2_i1|m.20031	10	MDCHRCGVSGCHLRIT--CSADEKFCY-KWYNKIIG-----IET-RGCAKTCTEENSWTASVYCCTTNLCNT*
28	*paraensis*	DN85432_c2_g3_i3|m.2650	10	MKCYRCGVSGCHLKTT--CAADEKFCY-KWYNKIIG-----IET-RGCAKTCTEENSWTASVYCCTTNLCNT*
29	*paraensis*	IACL01013323.1	10	MDCHRCGVSGCHLRIT--CSADEKFCY-KWYNKIIG-----IET-RGCAKTCTEENSWTASVYCCTTNLCNT*
30	*carvalhoi*	IACI01044553.1	10	MDCHRCGVSGCHLRIT--CSADEKFCY-KWYNKIIG-----IET-RGCAKTCTEENSWTARVYCCTTNLCNT*
31	*spixii*	IACM01149050.1	10	MDCHRCGVSGCHLRIT--CSADEKFCY-KWYNKIIG-----IET-RGCAKTCTEENSWTARVYCCTTNLCNT*

1	*carvalhoi*	IACI01064820.1	11	LECKICNFKTCPTDELRRCASGETICY-KKFCNTPHG---LRIDRGCAATCPTVKPGV-NIICCKTDNCN*
2	*spixii*	DN79827_c1_g17_i1|m.37079	11	MKCYKCGVSGCHLK--ITCSEDEKFCY-KWY--NKIC---IRT-HGCAKTCTEENSWTASVYCCTTNLCNT*
