# Supplementary material for: Addendum: Aird, S.D. et al. Coralsnake Venomics: Analyses of Venom Gland Transcriptomes and Proteomes of Six Brazilian Taxa. Toxins 2017, 9(6), 187
Source: Toxins (Basel). 2018 Apr 24;10(5):172. doi: 10.3390/toxins10050172 (PMC5982091; doi:10.3390/toxins10050172)
Supplement: Supplementary file 1 [file toxins-10-00172-s001.zip › supplementary/Figure S5.pdf]

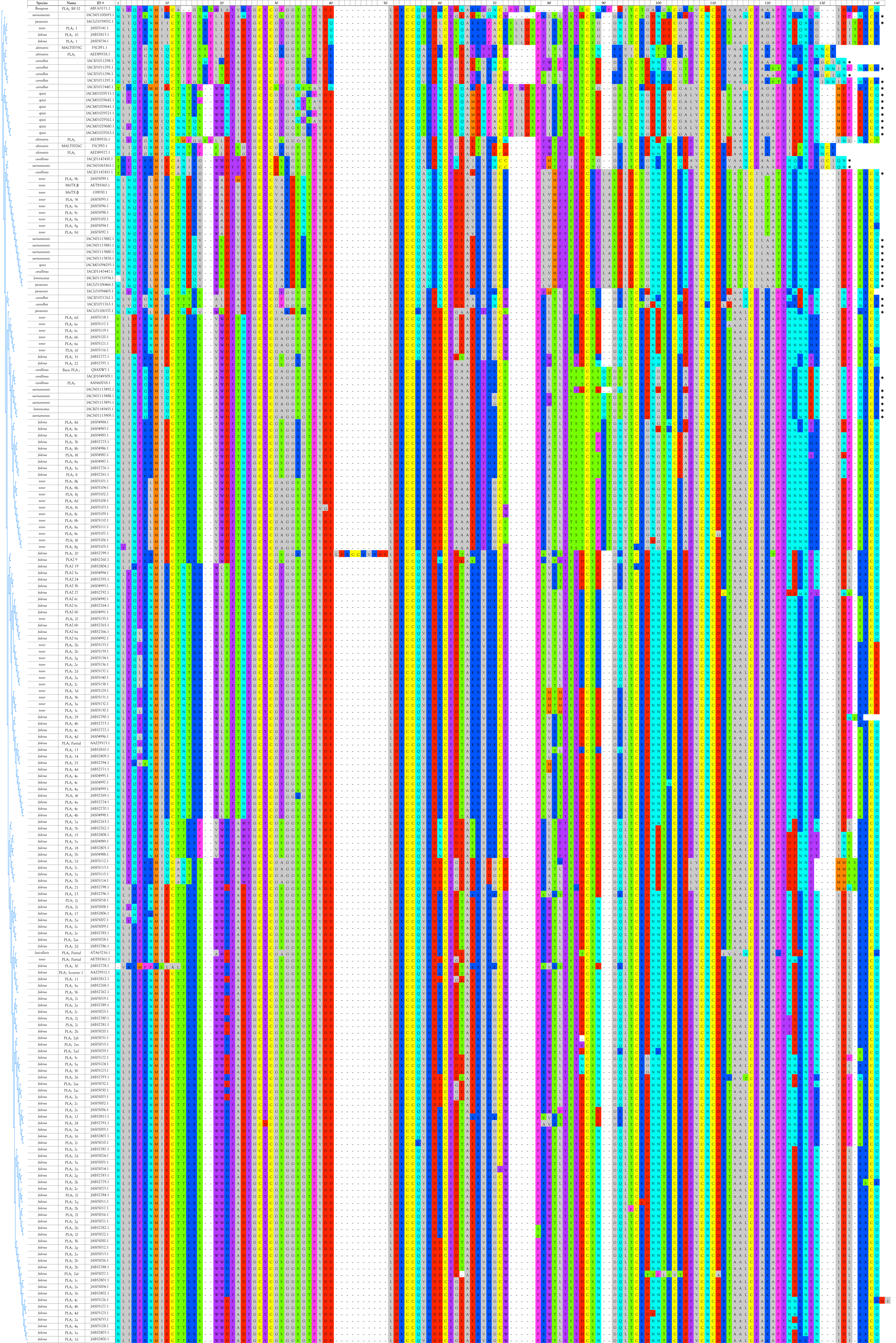
Species
Name
ID #
