# Supplementary material for: Addendum: Aird, S.D. et al. Coralsnake Venomics: Analyses of Venom Gland Transcriptomes and Proteomes of Six Brazilian Taxa. Toxins 2017, 9(6), 187
Source: Toxins (Basel). 2018 Apr 24;10(5):172. doi: 10.3390/toxins10050172 (PMC5982091; doi:10.3390/toxins10050172)
Supplement: Supplementary file 1 [file toxins-10-00172-s001.zip › supplementary/figure captions.docx]

**Figure S1.** Three disulfide bond patterns are evident among 8-cysteine 3FTxs from the venoms of *Micrurus corallinus*, *M. l. carvalhoi*, *M. l. lemniscatus*, *M. paraensis*, *M. spixii*, and *M. surinamensis*. The first pattern is illustrated by sequences 1-12, and includes the overwhelming majority of 8-Cys toxins. The second, represented by sequences 13-19, occurs in *M. l. carvalhoi*, *M. paraensis*, *M. lemniscatus*, and *M. surinamensis*. These toxins have four cysteines among the N-terminal 18 amino acids, like γ-bungarotoxin (positions 3, 6, 11, and 17), and they lack the two C-terminal cysteines (positions 65 and 70), including one of the usual paired residues. The third pattern is represented by sequences 57-60 and 92. In these toxins, the paired cysteines have become separated, with Cys-66 being displaced two residues C-terminally, such that the three C-terminal cysteines occur in positions 64, 67, and 70, instead of 64, 65, and 70. The functional significance of these patterns is unknown. A tree was constructed using the Neighbor-Joining method with the Jukes-Cantor model and γ-bungarotoxin from *Bungarus multicinctus* venom as an outgroup. Asterisks indicate stop codons, which were paired in some cases.

**Figure S2.** 3FTxs having 9 cysteines, derived from venoms of *Micrurus corallinus*, *M. l. carvalhoi*, *M. l. lemniscatus*, *M. paraensis*, *M. spixii*, and *M. surinamensis*, display two disparate disulfide bond patterns. The first, representing the majority of 9-Cys toxins, has paired second and third cysteines in positions 16-17 (sequences 1-28 and 30-31). In the second group, the cysteine in position 16 is absent and instead a cysteine is present in position 63. Whether the extra cysteine is free in monomeric toxins, or whether these 9-Cys toxins form homo- or heterodimers with a non-homologous toxin is unknown. γ-bungarotoxin from *B. multicinctus* venom was used as an outgroup. Asterisks indicate stop codons.

**Figure S3.** Venoms of *Micrurus corallinus*, *M. l. carvalhoi*, *M. l. lemniscatus*, *M. paraensis*, *M. spixii*, and *M. surinamensis* all have 3FTxs with 10 cysteines and our samples of *M. l. carvalhoi* and *M. spixii* also have novel toxins with an eleventh cysteine occurring at either position 32 or 37. The functional significance of these patterns is unknown. A tree was constructed using the Neighbor-Joining method with the Jukes-Cantor model and γ-bungarotoxin from *B. multicinctus* venom as an outgroup. Asterisks indicate stop codons.

**Figure S4.** South American *Micrurus* 3FTx sequences comprise as astonishing variety of structural subtypes, including many that have not been reported from the North American species, *M. fulvius* and *M. tener*. However, some subtypes are common to both groups. Previously published 3FTx sequences were downloaded from the NCBI nr database. γ-bungarotoxin from *B. multicinctus* venom was used as an outgroup. Asterisks indicate stop codons.

**Figure S5.** The *Micrurus* taxa investigated here rely much less heavily on PLA_2_ toxins than do the North American taxa, *M. fulvius* and *M. tener*. Still, various structural subclasses are evident, although differences in function cannot be surmised at present, except for probable non-catalytic PLA_2_s that lack the active site His-58 and/or Asp-59 residues (see main text). Most of the North American sequences cluster separately from the Brazilian sequences. PLA_2_ sequences from Brazilian *Micrurus* species were identified with Megablast, using nine complete PLA_2_s, including signal peptides, as queries. 35 Brazilian *Micrurus* PLA_2_s are aligned here with 169 published sequences, using Geneious software. A tree was constructed using the Neighbor-Joining method with the Jukes-Cantor model, with *Bungarus fasciatus* PLA_2_ BF-32 as an outgroup. Signal peptides are not shown in this alignment. Asterisks indicate stop codons.
